# Predicting RP-LC retention indices of structurally unknown chemicals from mass spectrometry data

**DOI:** 10.1186/s13321-023-00699-8

**Published:** 2023-02-24

**Authors:** Jim Boelrijk, Denice van Herwerden, Bernd Ensing, Patrick Forré, Saer Samanipour

**Affiliations:** 1 AI4Science Lab, University of Amsterdam, Amsterdam, The Netherlands; 2 Institute for Informatics, University of Amsterdam, Amsterdam, The Netherlands; 3 Van’t Hoff Institute for Molecular Sciences (HIMS), University of Amsterdam, Amsterdam, The Netherlands; 4 Computational Chemistry Group, Van’t Hoff Institute for Molecular Sciences (HIMS), Amsterdam, The Netherlands; 5 UvA Data Science Center, University of Amsterdam, Amsterdam, The Netherlands; 6 Queensland Alliance for Environmental Health Sciences (QAEHS), The University of Queensland, Woolloongabba, Australia

**Keywords:** Non-target analysis, Retention indices, HRMS, Machine learning

## Abstract

Non-target analysis combined with liquid chromatography high resolution mass spectrometry is considered one of the most comprehensive strategies for the detection and identification of known and unknown chemicals in complex samples. However, many compounds remain unidentified due to data complexity and limited number structures in chemical databases. In this work, we have developed and validated a novel machine learning algorithm to predict the retention index (r$_i$) values for structurally (un)known chemicals based on their measured fragmentation pattern. The developed model, for the first time, enabled the predication of r$_i$ values without the need for the exact structure of the chemicals, with an $R^2$ of 0.91 and 0.77 and root mean squared error (RMSE) of 47 and 67 r$_i$ units for the NORMAN ($n=3131$) and amide ($n=604$) test sets, respectively. This fragment based model showed comparable accuracy in r$_i$ prediction compared to conventional descriptor-based models that rely on known chemical structure, which obtained an $R^2$ of 0.85 with an RMSE of 67.

**Supplementary Information:**

The online version contains supplementary material available at 10.1186/s13321-023-00699-8.

## Introduction

The human and environmental exposome contains a multitude of chemicals consisting of natural ones, man-made chemicals, and their transformation products, including their metabolites [1, 2]. These chemicals cover a wide range of molecular weights, functional groups or compound classes, physiochemical properties, and biological activities (i.e. toxicity) [2, 3]. Most of the chemicals in the human exposome are structurally unknown and therefore there is little known about their occurrence, fate, and potential health impact [4–9].

Non-target analysis (NTA) combined with high-resolution mass spectrometry (HRMS) is considered one of the most comprehensive strategies for the detection and identification of the unknown chemicals of emerging concern (CECs) in complex biological and environmental samples [2, 4, 8, 10–15]. The NTA experiments are reliant on generic experimental conditions as they aim to cover as wide a portion of the sample chemical space as possible [4, 8, 16, 17]. Moreover, the NTA experiments have the ultimate goal of confident identification (i.e. structural elucidation) of all the chemical constituents within the covered chemical space of the sample. This implies that the NTA experiments tend to generate a large number (e.g. thousands) of high-resolution mass spectra per sample to be structurally elucidated [8, 12, 14, 17–20].

In the past decade, a lot of efforts have been put into the generation of digital open-source/access data processing tools to tackle the complex data generated from the NTA assays [11, 12, 21–28]. These digital tools provide the means to perform a complete NTA workflow from feature detection [25, 29–31] to componentization [21, 31, 32] and identification/annotation [28, 33–36]. These tools, even though powerful, have shown to be highly sensitive toward the data quality and the parameters used during the processing [9, 37–40], particularly when dealing with complex samples [24, 41–43]. In addition, the inherent variability in the data caused by the experimental conditions used during the analysis significantly increases the difficulties associated with confidence assessment of the generated structures for the features in the chromatograms [8, 44–46]. Finally, a large number of the generated chromatographic features remain unidentified, due to data complexity, limited spectral databases [47], and limited structures in chemical databases (e.g. PubChem [48] and/or CompTox [49]), even though their accurate mass spectral information has been collected during the analysis.

To mitigate the issues related to the identification of known and unknown unknowns, the addition of retention times and retention indices (r$_{i}$), as an additional source of information, have been previously tested [50–58]. For r$_{i}$ measurements, a series of calibrants (i.e. chemicals with known retention behavior) are necessary. The simultaneous analysis of the samples and the r$_{i}$ calibrants under specified conditions has enabled the measurement of r$_{i}$ values of structurally unknown chemicals [59, 60]. These measured r$_{i}$ values are then compared to the r$_{i}$ databases of structurally known chemicals to further increase the associated confidence in the generated identifications [59–61]. Additionally, recent studies have highlighted the use of quantitative structure retention relationship (QSRR) models to predict and populate the r$_{i}$ databases, employing molecular descriptors [59–62]. The QSRR methods for the prediction of r$_{i}$ values of structurally known chemicals have been a complementary strategy to the experimentally defined r$_{i}$ values. However, these approaches have some major limitations namely: they require the chemical structure to be known; and for unknown chemicals are applicable only under well-defined chromatographic conditions (for example a specific organic modifier); and, the experimental r$_{i}$ calibrant information associated with each NTA experiment. The combination of the measured r$_{i}$ via calibrant chemicals and the predicted values via databases have been utilized for reducing the number of potential candidates and thus increasing the confidence levels associated with tentatively identified features [59–61]. However, for this workflow to be effective, the calibrants and the samples must be measured using the exact same experimental conditions. This implies that any changes in the experimental conditions (e.g. gradient, organic modifier, column temperature) will warrant additional measurements of the calibrants via the new methods. Moreover, it should be noted that for most NTA studies, already published, the r$_{i}$ calibrants are not injected and thus their retention times missing [4]. This limitation also hinders the alignment of chromatograms acquired under different experimental conditions (i.e. either different labs or experimental setups), ultimately slowing down the process of detection of chemicals of emerging concern [26]. Such aforementioned limitations, greatly limits the applicability of r$_{i}$ values for unraveling the human and environmental exposome via NTA assays.

Here we have developed and validated a novel machine learning algorithm to predict the r$_{i}$ values for structurally unknown chemicals based on their measured fragmentation pattern. The developed models, for the first time, enable the prediction of r$_{i}$ values without the need of the exact structure of the chemicals. For the model development, we selected the alkylamides homologous series as the r$_{i}$ scale, based on their range of applications in the NTA metabolomics studies [59, 61]. The r$_{i}$ values for structurally known chemicals were predicted using both descriptors as well as the fragmentation patterns translated into cumulative neutral losses (CNL). The CNL values were obtained by calculating the difference between the precursor mass and individual fragments within the high-resolution mass spectrometry (HRMS) spectra assuming complete independence among fragments. The CNL based model was validated employing both experimental r$_{i}$ values and descriptor-based predicted r$_{i}$ values. Finally, the validated CNL-based model showed comparable accuracy in r$_{i}$ prediction to conventional descriptor-based models, relying only on the measured HRMS spectra (i.e. no information about the chemical structures).

## Methods

### Data

For the model development, validation, and testing we employed two different datasets namely: a set of experimental r$_{i}$ values - referred to as amide dataset - based on the alkylamides homologous series, consisting out of 1488 chemicals [59]; and 26489 chemicals from the NORMAN SusDat database [26]. The alkylamides homologous series is one of the most commonly used r$_{i}$ scale for C18 reversed phase liquid chromatography (RP-LC) due to their ease of measurement with RP-LC-HRMS and their applications in the metabolomics field [59, 61]. The amide dataset consisted of measured r$_{i}$ values for 1488 chemicals with more than 40 different functional groups from amine, aniline, pyridine, pyrrole, ether, ester, ketone, alcohol, carboxylic acid, phenol to amide and molecular weight range between 79 Da and 609 Da. These r$_{i}$ values were measured using a Zorbax SBC18 column and the combination of water and acetonitrile as the mobile phase. More details on these measurements are provided elsewhere (Hall et al 2016 [59]).The NORMAN dataset, on the other hand, was selected based on the high level of curation and availability of experimental HRMS spectra through NORMAN MassBank [63].

**Fig. 1:**
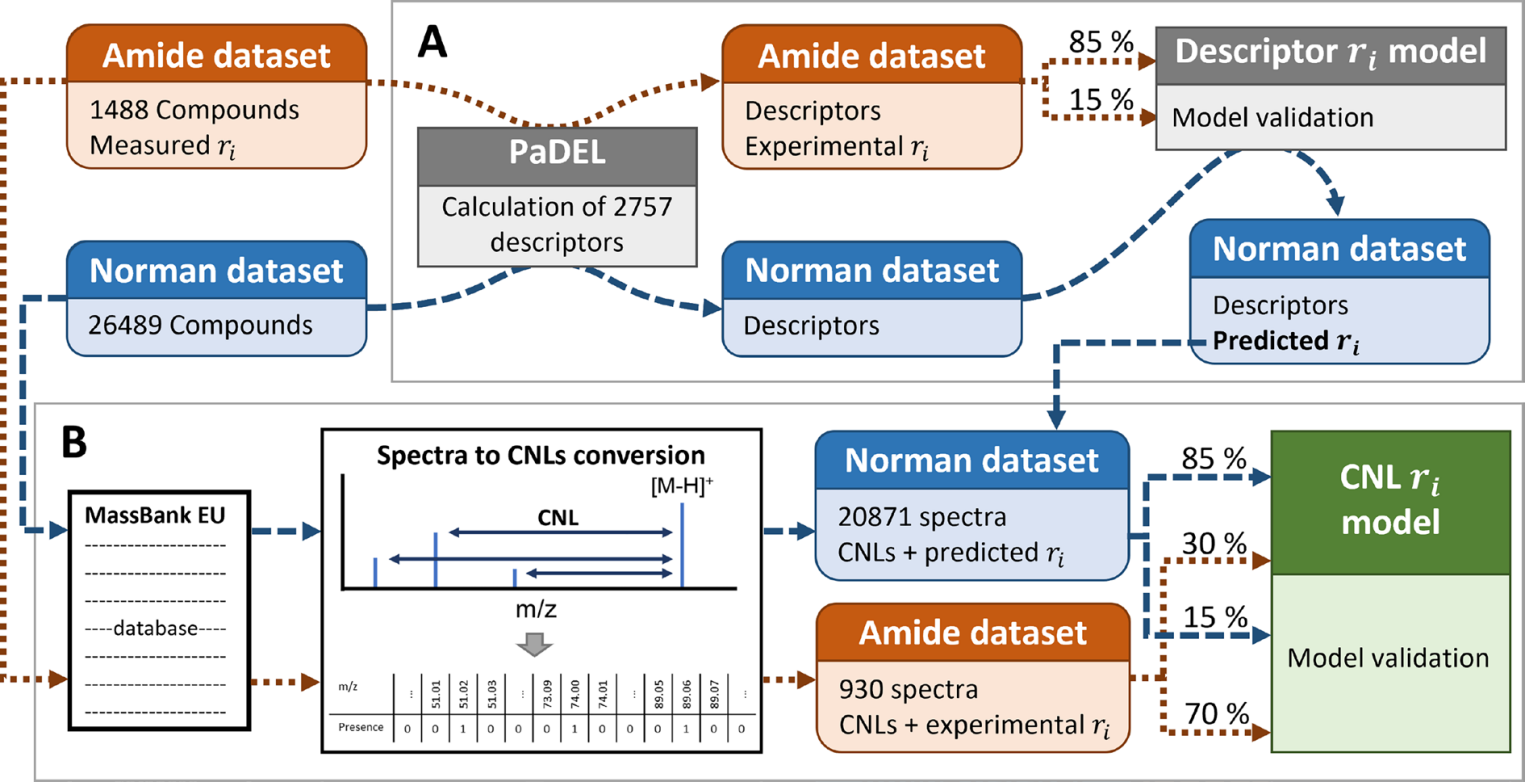
Workflow for setting up the models for predicting r$_i$ values. **A** shows the construction of the descriptor model for predicting the NORMAN r$_i$ values, whereas, **B** shows the conversion of spectra to CNL values and the construction of the CNL model

We calculated 2757 1D, 2D, 3D, and PubChem fingerprints for both datasets using the PaDEL software package (Fig. 1) [64]. The curated amide dataset descriptors were employed for the development and validation of an r$_{i}$ prediction model. This was done due to the fact that only 133 unique chemicals out of 1488 had their experimental HRMS spectra available in public spectral databases. This descriptor-based model then was utilized to predict the r$_{i}$ values for the NORMAN dataset, which contained 3217 unique chemicals with experimental HRMS spectra (around 20871 measured spectra). We combined 30$\%$ of the amide dataset with experimental r$_{i}$ values and HRMS spectra with 85$\%$ of the NORMAN dataset with predicted r$_{i}$ values (descriptor based model) and experimental HRMS spectra. This combination enabled us to minimize the impact of the first model (i.e. descriptor based model) on the CNL based model, while enabling an adequate validation of the model. Finally, the remaining 70$\%$ of the amide dataset with experimental r$_{i}$ values and HRMS spectra were employed to further test the performance the CNL based model. This workflow is schematically shown in Fig. 1.

Additionally, we further compared the chemical spaces covered by our training set as well as the NORMAN dataset. We performed a principal component analysis using the curated descriptors for both datasets. More details of the PCA are provided in Section S2 of the Additional file 1. The scores plot of the first two PCs indicates that our training set provides an adequate coverage of the NORMAN dataset (see Additional fie 1: Figs. S6 and S7).

#### Descriptor datasets

*Descriptor generation:* The PaDEL software was employed for the calculation of 2757 1D, 2D, 3D, and PubChem fingerprints for the amide and NORMAN datasets [64]. The predicted descriptors were saved as CSV files for the amide and NORMAN datasets, respectively and can be found on FigShare (see Sect. "Potentials and limitations"). When performing the 3D descriptor calculations, PaDEL needed to optimize the chemical structures, which in some cases resulted in convergence issues, and thus a failure in descriptor calculation. The descriptor calculations converged for 1289 out of 1488 unique chemicals in the amide dataset and 23012 out of 26489 unique chemicals from the the NORMAN dataset.

*Descriptor curation:* We also performed a descriptor curation to assure that only the relevant and stable descriptors were included in the models. To assess the stability of the descriptors, we performed these calculations in triplicates for the amide dataset. Next, the descriptors were scaled based on the minimum and maximum value of each descriptor in order to compare them at the same scale. Then we calculated the variance of each descriptor and kept only the descriptors that had a variance lower than 0.01. This resulted in 2363 final stable descriptors to be used for the models. As for the NORMAN dataset, the descriptors were calculated only once, due to the large number of chemicals. It was assumed that the descriptors that were deemed stable in the amide dataset, had a high probability of being stable also for the NORMAN dataset.

#### The retention index values (r$_{i}$)

**Fig. 2:**
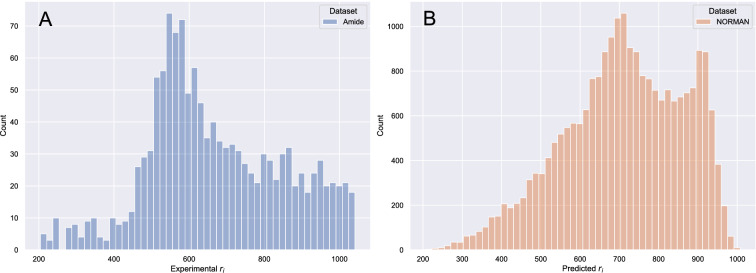
Distribution of r$_i$ values for the descriptor amide dataset (**A**) and of predicted r$_i$ values for the descriptor NORMAN dataset (**B**)

While for the compounds in the amide dataset r$_{i}$ values were determined experimentally, this was not the case for the compounds in the NORMAN dataset. Therefore, r$_{i}$ values for the NORMAN dataset were predicted using the descriptor based model (see Sect. "Modeling"). The prediction of the r$_{i}$ values of the NORMAN dataset was performed only for the chemicals that were within the applicability domain (AD) of the descriptor based model (see Sect. "Applicability domain"). The AD filtering of the NORMAN dataset resulted in reliably predicted r$_{i}$ values for 14567 unique chemicals chemicals. The distribution of the r$_{i}$ values used in this study can be seen in Fig. 2.

#### CNL dataset

**Fig. 3:**
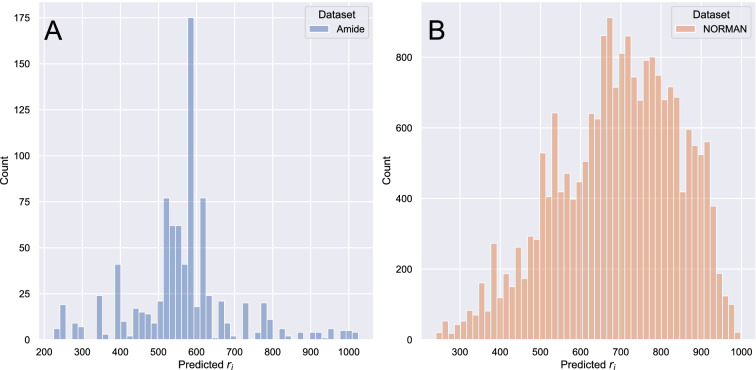
Distribution of r$_i$ values for the CNL amide dataset (**A**) and of the predicted r$_i$ values for the CNL NORMAN dataset (**B**)

For the CNL dataset, electrospray ionization (ESI) high resolution (i.e. $\ge$ 5000) spectra were obtained from MassBank EU [65] for both the amide and NORMAN compounds. For each of these chemicals, all corresponding experimental spectra were obtained based on the InChiKeys [66] and SMILES [67]. For the amide dataset, a total of 862 mass spectra were found for 133 unique compounds. Whereas for the NORMAN dataset, a total of unique 23871 mass spectra were found for 3217 unique chemicals. These compounds were cross-referenced with the NORMAN descriptor dataset, from which it was concluded that only for 2734 unique compounds, reliable r$_i$ values could be predicted, resulting in a dataset of 20871 entries. The distribution of r$_i$ values for the amide and NORMAN datasets can be seen in Fig. 3A, B, respectively. The retrieved HRMS spectra were generated by different labs, instruments, and under different experimental conditions. In order for our model to be able to handle the variance in the spectra coming from these different experimental settings, we kept the redundant spectra of the same compound as separate entries. For example, for caffeine there were around 50 measured spectra with different instruments, collision energies and instrumental setups (e.g. source geometry and temperature). However, we were not able to see a direct relationship between different experimental parameters and the number, m/z value, and the relative intensity of the generated fragments. For example, the spectrum generated with orbitrap at 10 eV (AU276601) resulted in 2 fragments while the spectrum via a QToF instrument at 30 eV resulted also in 2 fragments (KW107903). For our model to be robust enough to handle such instrument related variance, we kept all those spectra as separate entries in our model. This strategy enabled us to incorporate the instrument variability into our models, without compromising the model accuracy.

For both the amide and NORMAN datasets, the CNLs were calculated for each individual spectrum by subtracting the fragment masses from the precursor ion mass. For each spectrum, the CNL values were converted to a bit vector, corresponding to CNLs masses from 0 to 1000 Da with a step size of 0.01 Da (i.e. ± 5 mDa mass tolerance), where a 1 represented the presence and a 0 indicated the absence of a CNL within a spectrum. Additionally, the CNL values larger than the precursor ion were encoded as -1 in the bit vector. This was to make sure that the model can distinguish between absent and impossible CNLs (i.e. fragments with m/z values larger than precursor m/z). The precursor ion mass was added as an additional continuous feature to the dataset. The combination of the monoisotopic mass and the CNLs enabled us to incorporate the information provided by the individual fragments and NLs into our model while containing the number of model variables to minimum. For example in case of CNL of 18 Da (i.e. the loss of water), the number of variables were reduced $\approx$ 3000 variables (i.e. individual fragments) to one single variable without any loss of information.

### Modeling

For modeling, a gradient boosting regression model was implemented in Python 3.7.11 using CatBoost (v 0.3) [68]. CatBoost is a state-of-the-art approach for gradient boosting on decision trees for big data [69]. The main idea of gradient boosting is to consecutively combine many decision trees (weak learners) to create a strong competitive model. Since the decision trees are fitted consecutively, the fitted trees will learn from the mistakes of former trees to reduce errors. The process of adding new trees to existing ones is continued until the selected loss function is no longer minimized or the maximum tree depth is obtained.

*Descriptor based model:* To train the descriptor based model, the amide dataset was split into a training set (85%, n=1102) and a test set (15%, n=195). The descriptor based model had calculated and curated descriptors of the amide dataset as the input variables, while having the experimental r$_{i}$ values as the output variable (see Fig. 2). To reflect the skewed nature of the r$_{i}$ values, the data splitting was performed utilizing stratified sampling. This ensured that both the training set and test set had a good representation of the population. The stratified sampling was done using three classes, from 200–440, 440–700, and 700–1041 r$_{i}$ units.

The model training and optimization was performed using the root mean square error (RMSE) loss function for a total of 450 iterations (a tree is constructed every iteration) with a learning rate of 0.03. The tree depth was set to 8 with a maximum number of 256 leaves. The minimum data in a leaf was set to 1. To prevent overfitting the coefficient of the L2 regularization term was set to 10, which showed to provide the needed balance between the model accuracy and robustness. In addition, the training was stopped if the error on the validation set did not decrease for more than 5 iterations. For the remaining parameters, the default values were used. Additional information regarding the hyperparameter selection can be found in Additional file 1: Section S3. We used 5-fold cross-validation to tune model hyper-parameters on the training set. Accordingly, the training set was split into five equally sized parts, which were used to construct 5 different training and validation splits. Additionally, from the optimized model, we extracted the 40 features resulting in highest levels of variance explained in the training set. The distributions of these features are shown for both the amide and NORMAN dataset in Additional file 1: Section S4.1. The final model was then refitted using these 40 features and optimized model hyper-parameters, which consisted out of 448 trees, on the whole training set.

To further assess the model performance, we took advantage of the test set that was unknown to the model during the training step.

*CNL model:* A CatBoost regression model was built utilizing the CNL datasets as input in order to predict r$_{i}$ values. The model was trained using the RMSE loss function for a total of 5000 iterations with a learning rate of 0.077. The tree depth was set to 6 with a maximum number of 64 leaves, with a minimum number of data points of 1 per leaf. The coefficient of the L2 regularization term was set to 3, and the training was stopped if the error on the validation set did not decrease for more than 5 iterations, to prevent overfitting. Again, the default values were used for the remaining parameters. Additional information regarding the hyperparameter selection can be found in Additional file 1: Section S3. The model was trained on a combination of the amide and NORMAN datasets. The NORMAN dataset was split into a training set (85$\%$, n=17740) and a test set (15$\%$, n=3131). And the amide dataset was split into a training set (30$\%$, n=258) and a test set (70$\%$, n=604), resulting in a total training set of 17998 entries, consisting of unique spectra. It should be noted that only a small fraction of the amide dataset was used for training, enabling the final testing of the model with experimental r$_{i}$ values that were not included in the training set. The model was then trained and optimized using 5-fold cross-validation on the training set (as in the paragraph above). This resulted in a final model with 5000 trees and 4220 used features out of the 100,000 CNLs. Distributions of the 50 most important CNLs are shown in Additional file 1: Section S4.2 for both the amide and NORMAN dataset. Both test sets (i.e. the test set for the data splitting and the amide withheld dataset) were used as external test sets after the training process to reliably assess model performance.

### Applicability domain (AD)

In order to assess whether a new entry is represented by the training set, we employed the applicability domain (AD) calculations. The AD was determined using the leverage [70] ($h_{ii}$), which is defined as follows:

1 $$\begin{aligned} h_{ii} = \varvec{x}_{i}^{\top }\left( \textbf{X}^{\top } \textbf{X}\right) ^{-1} \varvec{x}_{i} \end{aligned}$$

Where $\textbf{X}$ is a matrix of descriptors for compounds from the (e.g. amide) training set, and $\varvec{x}_i$ is a vector of molecular descriptors for a compound *i*. The leverage score can be viewed as the weighted distance between $\varvec{x}_i$ and the mean of $\textbf{X}$, which therefore provides a measure of the applicability domain of the model. The acceptable threshold leverage value was determined as the 95% confidence interval using the distribution of leverages generated using a leave-one-out approach on the training set (this was $\approx$ 0.131). This threshold was used for assessing whether a chemical was well covered by the training set. The same approach was employed for the AD assessment of the CNL model. All distributions of the training and test sets can be found in Additional file 1: Section S1. It should be noted that this AD assessment only takes into account the variables used in the final model, which could be inadequate for future entries.

### Calculations

All calculations were performed using a personal computer with an AMD Ryzen Threadripper 3970X CPU and 256GB of RAM operating on Windows 10 Pro. All the data processing and statistical analysis were performed using Python 3.7.11 and using Julia language 1.6.0 For descriptor calculations a Python 3 wrapper of PaDEL software called padelpy (https://github.com/ecrl/padelpy) was employed.

## Results and discussion

### Descriptor based model

The first model developed in this study was a QSRR model of the 2363 curated descriptors for 1289 unique chemicals and their experimental r$_{i}$ values. This model was then optimized and validated with an external test set. Next the validated model was employed to predict the r$_{i}$ values for the NORMAN dataset, providing a large enough training set for the CNL based model.

#### Descriptor based model performance

**Fig. 4:**
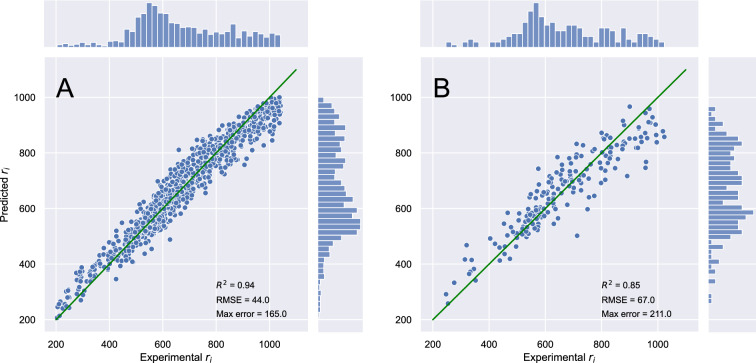
Parity plot of the descriptor model predictions and the experimental r$_i$ values for the training set (n=1102) (**A**) and the external test set (n=195) (**B**) with the coefficient of determination ($R^2$), root mean squared error (RMSE) and maximum error. In addition, marginal distributions of the experimental and predicted r$_i$ are shown

The optimized descriptor based model was able to successfully and accurately predict r$_{i}$ values with a standard error of 4.9– − 7.5%, for the training and test sets, respectively. The quality of the data fit is shown in Fig. 4. To assess the performance of the model, both the coefficient of determination ($R^2$), the root mean squared error (RMSE), and the maximum error were evaluated. The model showed regression statistics with $R^2 = 0.94$ for the training set and $R^2 = 0.85$ for the test set. The RMSE of the CatBoost model was 44 r$_{i}$ units for the training set and 67 r$_{i}$ units for the test set, which roughly is 4.9$-$7.5% of standard error. Interestingly, based on the distribution of residuals, the model seems to be consistently overestimating for low (200–400) r$_{i}$ values and underestimating for high (900–100) r$_{i}$ values. The worst prediction was off by 165 r$_{i}$ units for the training set data and 211 r$_{i}$ units for the test set.

#### Interpretation of selected descriptors

When looking into the 7 most important descriptors selected by the final model (Shown in Additional file 1: Section S5 of the Additional file), consistency in the information behind the descriptors can be found. All 7 most important descriptors are 2D descriptors and are directly or indirectly related to the charge of the molecule, which is a highly important factor for the separation with reversed-phase chromatography (RP-LC) [50]. The first three descriptors are describing a variant of LogP (i.e., partition coefficient), namely, the XlogP, Mannhold LogP, and the Crippen LogP. The next important descriptors are the number of basic groups (nBase), the Lipoaffinity index, and the centered moreau-broto autocorrelation of lag 3 weighted by mass (ATSC3m), which contains information on the topological structure. These descriptors all represent the chemical interactions with the stationary and mobile phases. Lastly, the 7th most important variable is the BCUTw-1 h [71], which corresponds to the lowest eigenvalue obtained from information based on the atomic charge, polarizability, and hydrogen-bond donor and acceptor capabilities. Overall, it is logical that descriptors containing information on the charge of the molecules have the highest contributions to the prediction of RPLC r$_{i}$ values.

### CNL based model

Using the descriptor based model we predicted the r$_{i}$ values of 2734 unique chemicals part of the NORMAN dataset, that were within the AD of our model. These 2734 chemicals resulted in 20871 HRMS entries from the MassBank EU and were combined with 258 entries belonging to the amide dataset. The generated CNL matrix and the vector of r$_{i}$ values were utilized to build a model, which was able to predict the r$_{i}$ values only based on the HRMS spectra. Additionally, 604 entries from the amide dataset were used for further testing of the final model. These 604 entries had both the CNL matrix and r$_{i}$ values experimentally determined and were completely unknown to our model.

#### CNL based model performance

The final model showed correlation statistics with an $R^2 = 0.96$ for the training set and $R^2 = 0.91$ for the test set. On the other hand for the withheld 604 entries from the amide dataset, an $R^2=0.77$ was produced by our model. The worst prediction error was for the additional amide dataset and was of 283 r$_{i}$. On the other hand, the RMSE for the model was 30 r$_{i}$ and 47 r$_{i}$ units for the training and test sets, respectively while resulting in 67 r$_{i}$ units for the additional amide test set. This further indicates that our model is able to accurately predict the r$_{i}$ values based on the HRMS spectra.

**Fig. 5:**
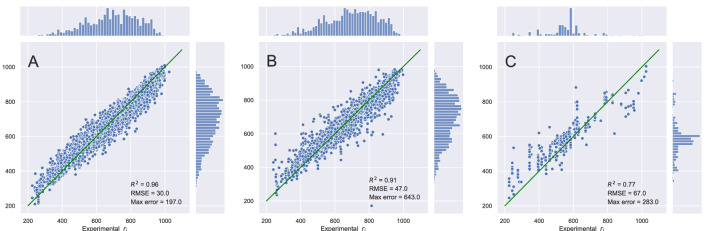
Parity plot of the CNL model predictions and the experimental r$_i$ values for the training set (n=17998) (**A**) the external NORMAN test set (n=3131) (**B**) and the external amide test set (n=604) (**C**) with the coefficient of determination ($R^2$), root mean squared error (RMSE) and maximum error. In addition, marginal distributions of the experimental and predicted r$_i$ are shown

When comparing the performance of the model on the different test sets, it is evident that the performance is lower for the additional amide test set compared to the conventional test set. This is not surprising, as the training set is mostly comprised of the NORMAN dataset (17740 out of 17998 entries), for which the r$_{i}$ values were predicted using the descriptor based model resulting in error propagation and thus lower accuracy. It is therefore rather impressive that the model still is able to explain more than 77 % of the variance of the additional amide test set. This is a strong confirmation that CNLs can effectively be used to predict r$_{i}$ values, indicating that an increase in experimentally determined retention indices and HRMS spectra could further improve the model accuracy. Another point of attention is the number of features that have been used by the model to make its predictions. As we are dealing with a setting in which we have more features ( 100 000) than measurements (17998 r$_{i}$ values), care needs to be taken in model construction that the model uses fewer features than there are data points to avoid a potential under-determination issue. This was addressed using L2 leaf regularization and early stopping as described in Sect. "Methods". Yet for the generalization of the model, it is important for the model to incorporate many features (read different CNLs) as a molecule can potentially fragment into different fragments and the model should take these into account. Also, measurement noise may change CNL masses slightly (and therefore feature values), which should also be able to be modeled. Again we emphasize that training on larger data sets could naturally capture this. We feel that our final model trained on the dataset at hand, utilizing 4220 features, is balancing the aforementioned trade-off well (Fig. 5).

#### Interpretation of selected CNL features

More information on the selected CNL features are shown in Additional file 1: Section S6 of the Additional file. When investigating the most important features of the CNL based model, a few consistent structures were found for specific CNLs by checking the annotated spectra on MassBank EU. Among those, the 3 most important CNLs for which similarity was found, will be discussed as examples. However, it should be noted that for the larger CNLs it is generally more difficult to build such relationships between the r$_{i}$ values and the CNL values, due to an exponential increasing number of possible structures.

One of the most important CNLs had a mass of 155.00 Da, which was present in 365 spectra, corresponding to 30 unique chemical constituents. A majority of these chemicals contained the structure C2(=CC=C(C=C2)N)[S](=O)=O, which is a common structural feature observed in several antibacterial chemicals. Two other examples are the CNLs of 65.97 and 56.06 Da, which showed to be consistent with a loss of SO$_2$ and C$_4$H$_8$, respectively. Finally, the monoisotopic mass was also highly important to the CNL based model, which is understandable due to the direct relationship between the molecular weight and its retention behavior. Overall, these results show that CNL contains enough structural information on the functional groups and/or molecular substructures to be used for the prediction of r$_{i}$ values, however, alarger experimental dataset may further improve these interpretations.

### Potentials and limitations

In this work, we showed a novel approach for predicting r$_{i}$ values of the structurally unknown compounds using CNLs obtained from public HRMS spectra. The model requires no prior chemical or structural information, which enables the use of the model for NTA where both known and unknown chemicals can be encountered. The developed model enables a calibrant free use of r$_{i}$ values in NTA experiments. In other words, the analyst can predict the r$_{i}$ values of every single feature in the LC-HRMS chromatogram without knowing the chemical structure of the feature or without the need for measuring the r$_{i}$ calibrants. Besides the possibility of obtaining r$_{i}$ for unknown chemicals from CNLs, the model can also be used to, for example, enhance the performance of library searching and analysis of historical data. Specifically for cases where no r$_{i}$ calibrants were measured. Being able to predict the r$_{i}$ values for these cases can enhance library searching by reducing the number of initial candidates based on r$_{i}$ filtering. Also, the predicted r$_{i}$ values for historical data enables their alignment, independently from the experimental setup, which consequently provides the means for performing trend analysis and detection of novel unknown CECs. Additionally, r$_{i}$ values across the full chromatogram (i.e. pixel-by-pixel) could be mapped, providing insights into the chemical space covered by the analysis method. This range, also, gives insight into which compounds can and cannot be analyzed with one method vs another. Finally, the model could also be used for cross selectivity tracking. For example, if the current RPLC r$_{i}$ model would be used for a HILIC method, a reversed order of retention indexes is expected due to opposing selectivity modes. Overall, r$_{i}$ prediction from CNLs could potentially be used for a variety of applications in resolving human exposome.

One of the current limitations of the model is the number of chemicals with measured r$_{i}$ and HRMS spectra, impacting our models AD. Expansion of such measurements will be part of a near future study. To expand its AD and potentially the model confidence, larger spectral databases and more measured r$_{i}$ values for a specific selectivity would be required. Additionally, the current version of the model is trained using clean spectra. Therefore, in the case of compounds that are co-eluting during an LC-HRMS measurement, the obtained r$_{i}$ values could be less accurate due to the presence of false positive fragments in the spectrum. However, an adequate spectral clean-up and deconvolution could mitigate the above mentioned issue.

## Supplementary Information

**Additional file 1: Additional figures.**

## Data Availability

The algorithms for r$_i$ prediction from descriptors and CNLs, including the trained models and leverage matrices are available at: https://github.com/Jimbo994/NL2RI. In addition, a Google Collab tutorial is made available explaining usability of the models and creation of CNL from HRMS data. All datasets can be found at https://figshare.com/projects/Predicting_RP-LC_retention_indices_of_structurally_unknown_chemicals_from_mass_spectrometry_data/138373

## Abbreviations

r$$_i$$: Retention index
RMSE: Root mean squared error
$R^2$: Coefficient of determination
NTA: Non-target analysis
HRMS: High resolution mass spectrometry
CECs: Chemicals of emerging concern
QSRR: Quantitative structure retention relationship
CNL: Cumulative neutral losses
RP-LC: Reversed phase liquid chromatography
AD: Applicability domain
ESI: Electrospray ionization

## References

[1] Vermeulen R, Schymanski EL, Barabási AL, Miller GW (2020). The exposome and health: where chemistry meets biology. Science.

[2] Escher BI, Stapleton HM, Schymanski EL (2020). Tracking complex mixtures of chemicals in our changing environment. Science.

[3] Reymond JL, Van Deursen R, Blum LC, Ruddigkeit L (2010). Chemical space as a source for new drugs. Med Chem Comm.

[4] Schulze B, Jeon Y, Kaserzon S, Heffernan AL, Dewapriya P, O’Brien J, Gomez Ramos MJ, Ghorbani Gorji S, Mueller JF, Thomas KV, Samanipour S (2020). An assessment of quality assurance/quality control efforts in high resolution mass spectrometry non-target workflows for analysis of environmental samples. TrAC Trends Anal Chem.

[5] Muir DCG, Howard PH (2006). Are there other persistent organic pollutants? A challenge for environmental chemists. Environ Sci Technol.

[6] Lohmann R, Breivik K, Dachs J, Muir D (2007). Global fate of POPs: Current and future research directions. Environ Poll.

[7] Howard PH, Muir DCG (2011). Identifying new persistent and bioaccumulative organics among chemicals in commerce II: pharmaceuticals. Environ Sci Technol.

[8] Schymanski EL, Singer HP, Slobodnik J, Ipolyi IM, Oswald P, Krauss M, Schulze T, Haglund P, Letzel T, Grosse S, Thomaidis NS, Bletsou A, Zwiener C, Ibáñez M, Portolés T, De Boer R, Reid MJ, Onghena M, Kunkel U, Schulz W, Guillon A, Noyon N, Leroy G, Bados P, Bogialli S, Stipaničev D, Rostkowski P, Hollender J (2015). Non-target screening with high-resolution mass spectrometry: Critical review using a collaborative trial on water analysis. Anal Bioanal Chem.

[9] Samanipour S, Martin JW, Lamoree MH, Reid MJ, Thomas KV (2019). Letter to the editor: optimism for nontarget analysis in environmental chemistry. Environ Sci Technol.

[10] Werner E, Heilier J-F, Ducruix C, Ezan E, Junot C, Tabet J-C (2008). Mass spectrometry for the identification of the discriminating signals from metabolomics: current status and future trends. J Chromatogr B.

[11] Samanipour S, Reid MJ, Bæk K, Thomas KV (2018). Combining a deconvolution and a universal library search algorithm for the nontarget analysis of data-independent acquisition mode liquid chromatography-high-resolution mass spectrometry results. Environ Sci Technol.

[12] Samanipour S, Kaserzon S, Vijayasarathy S, Jiang H, Choi P, Reid MJ, Mueller JF, Thomas KV (2019). Machine learning combined with non-targeted LC-HRMS analysis for a risk warning system of chemical hazards in drinking water: a proof of concept. Talanta.

[13] Brack W, Hollender J, de Alda ML, Müller C, Schulze T, Schymanski E, Slobodnik J, Krauss M (2019). High-resolution mass spectrometry to complement monitoring and track emerging chemicals and pollution trends in European water resources. Environ Sci Eur.

[14] Schulze B, van Herwerden D, Allan I, Bijlsma L, Etxebarria N, Hansen M, Merel S, Vrana B, Aalizadeh R, Bajema B, Dubocq F, Coppola G, Fildier A, Fialová P, Frøkjær E, Grabic R, Gago-Ferrero P, Gravert T, Hollender J, Huynh N, Jacobs G, Jonkers T, Kaserzon S, Lamoree M, Le Roux J, Mairinger T, Margoum C, Mascolo G, Mebold E, Menger F, Miège C, Meijer J, Moilleron R, Murgolo S, Peruzzo M, Pijnappels M, Reid M, Roscioli C, Soulier C, Valsecchi S, Thomaidis N, Vulliet E, Young R, Samanipour S (2021). Inter-laboratory mass spectrometry dataset based on passive sampling of drinking water for non-target analysis. Scientific Data.

[15] Hollender J, Schymanski EL, Singer HP, Ferguson PL (2017). Nontarget screening with high resolution mass spectrometry in the environment: ready to go?. Environ Sci Technol.

[16] Andra SS, Austin C, Patel D, Dolios G, Awawda M, Arora M (2017). Trends in the application of high-resolution mass spectrometry for human biomonitoring: an analytical primer to studying the environmental chemical space of the human exposome. Environ Int.

[17] Gosetti F, Mazzucco E, Gennaro MC, Marengo E (2016). Contaminants in water: non-target UHPLC/MS analysis. Environ Chem Lett.

[18] Martínez-Bueno MJ, Gómez Ramos MJ, Bauer A, Fernández-Alba AR (2019). An overview of non-targeted screening strategies based on high resolution accurate mass spectrometry for the identification of migrants coming from plastic food packaging materials. TrAC Trends Anal Chem.

[19] Sobus JR, Wambaugh JF, Isaacs KK, Williams AJ, McEachran AD, Richard AM, Grulke CM, Ulrich EM, Rager JE, Strynar MJ, Newton SR (2017). Integrating tools for non-targeted analysis research and chemical safety evaluations at the US EPA. J Exposure Sci Environ Epidemiol.

[20] Dulio V, Koschorreck J, van Bavel B, van den Brink P, Hollender J, Munthe J, Schlabach M, Aalizadeh R, Agerstrand M, Ahrens L, Allan I, Alygizakis N, Barcelo’ D, Bohlin-Nizzetto P, Boutroup S, Brack W, Bressy A, Christensen JH, Cirka L, Covaci A, Derksen A, Deviller G, Dingemans MML, Engwall M, Fatta-Kassinos D, Gago-Ferrero P, Hernández F, Herzke D, Hilscherová K, Hollert H, Junghans M, Kasprzyk-Hordern B, Keiter S, Kools SAE, Kruve A, Lambropoulou D, Lamoree M, Leonards P, Lopez B, López de Alda M, Lundy L, Makovinská J, Marigómez I, Martin JW, McHugh B, Miège C, O’Toole S, Perkola N, Polesello S, Posthuma L, Rodriguez-Mozaz S, Roessink I, Rostkowski P, Ruedel H, Samanipour S, Schulze T, Schymanski EL, Sengl M, Tarábek P, Ten Hulscher D, Thomaidis N, Togola A, Valsecchi S, van Leeuwen S, von der Ohe P, Vorkamp K, Vrana B, Slobodnik J (2020). The NORMAN association and the european partnership for chemicals risk assessment (PARC): let’s cooperate!. Environ Sci Eur.

[21] van Herwerden D, O’Brien JW, Choi PM, Thomas KV, Schoenmakers PJ, Samanipour S (2022). Naive Bayes classification model for isotopologue detection in LC-HRMS data. Chemomet Intell Lab Syst.

[22] Alygizakis NA, Samanipour S, Hollender J, Ibáñez M, Kaserzon S, Kokkali V, Van Leerdam JA, Mueller JF, Pijnappels M, Reid MJ, Schymanski EL, Slobodnik J, Thomaidis NS, Thomas KV (2018). Exploring the potential of a global emerging contaminant early warning network through the use of retrospective suspect screening with high-resolution mass spectrometry. Environ Sci Technol.

[23] Samanipour S, Choi P, O’Brien JW, Pirok BWJ, Reid MJ, Thomas KV (2021). From centroided to profile mode: machine learning for prediction of peak width in HRMS data. Anal Chem.

[24] Samanipour S, Baz-Lomba JA, Alygizakis NA, Reid MJ, Thomaidis NS, Thomas KV (2017). Two stage algorithm vs commonly used approaches for the suspect screening of complex environmental samples analyzed via liquid chromatography high resolution time of flight mass spectroscopy: a test study. J Chromatogr A.

[25] Samanipour S, Baz-Lomba JA, Alygizakis NA, Reid MJ, Thomaidis NS, Thomas KV (2017). Two stage algorithm vs commonly used approaches for the suspect screening of complex environmental samples analyzed via liquid chromatography high resolution time of flight mass spectroscopy: a test study. J Chromatogr A.

[26] Alygizakis NA, Oswald P, Thomaidis NS, Schymanski EL, Aalizadeh R, Schulze T, Oswaldova M, Slobodnik J (2019). NORMAN digital sample freezing platform: a European virtual platform to exchange liquid chromatography high resolution-mass spectrometry data and screen suspects in “digitally frozen” environmental samples. TrAC Trends Anal Chem.

[27] Ruttkies C, Schymanski EL, Wolf S, Hollender J, Neumann S (2016). MetFrag relaunched: incorporating strategies beyond in silico fragmentation. J Cheminform.

[28] Wang M, Carver JJ, Phelan VV, Sanchez LM, Garg N, Peng Y, Nguyen DD, Watrous J, Kapono CA, Luzzatto-Knaan T, Porto C, Bouslimani A, Melnik AV, Meehan MJ, Liu WT, Crüsemann M, Boudreau PD, Esquenazi E, Sandoval-Calderón M, Kersten RD, Pace LA, Quinn RA, Duncan KR, Hsu CC, Floros DJ, Gavilan RG, Kleigrewe K, Northen T, Dutton RJ, Parrot D, Carlson EE, Aigle B, Michelsen CF, Jelsbak L, Sohlenkamp C, Pevzner P, Edlund A, McLean J, Piel J, Murphy BT, Gerwick L, Liaw CC, Yang YL, Humpf HU, Maansson M, Keyzers RA, Sims AC, Johnson AR, Sidebottom AM, Sedio BE, Klitgaard A, Larson CB, Boya CAP, Torres-Mendoza D, Gonzalez DJ, Silva DB, Marques LM, Demarque DP, Pociute E, O’Neill EC, Briand E, Helfrich EJN, Granatosky EA, Glukhov E, Ryffel F, Houson H, Mohimani H, Kharbush JJ, Zeng Y, Vorholt JA, Kurita KL, Charusanti P, McPhail KL, Nielsen KF, Vuong L, Elfeki M, Traxler MF, Engene N, Koyama N, Vining OB, Baric R, Silva RR, Mascuch SJ, Tomasi S, Jenkins S, Macherla V, Hoffman T, Agarwal V, Williams PG, Dai J, Neupane R, Gurr J, Rodríguez AMC, Lamsa A, Zhang C, Dorrestein K, Duggan BM, Almaliti J, Allard PM, Phapale P, Nothias LF, Alexandrov T, Litaudon M, Wolfender JL, Kyle JE, Metz TO, Peryea T, Nguyen DT, VanLeer D, Shinn P, Jadhav A, Müller R, Waters KM, Shi W, Liu X, Zhang L, Knight R, Jensen PR, Palsson B, Pogliano K, Linington RG, Gutiérrez M, Lopes NP, Gerwick WH, Moore BS, Dorrestein PC, Bandeira N (2016). Sharing and community curation of mass spectrometry data with global natural products social molecular networking. Nat Biotechnol.

[29] Tautenhahn R, Bottcher C, Neumann S (2008). Highly sensitive feature detection for high resolution LC/MS. BMC Bioinform.

[30] Treviño V, Yañez-Garza IL, Rodriguez-López CE, Urrea-López R, Garza-Rodriguez ML, Barrera-Saldaña HA, Tamez-Peña JG, Winkler R, Díaz De-La-Garza RI (2015). GridMass: a fast two-dimensional feature detection method for LC/MS. J Mass Spectrometr.

[31] Kenar E, Franken H, Forcisi S, Wörmann K, Häring HU, Lehmann R, Schmitt-Kopplin P, Zell A, Kohlbacher O (2014). Automated label-free quantification of metabolites from liquid chromatography-mass spectrometry data. Mol Cell Proteom.

[32] Kuhl C, Tautenhahn R, Böttcher C, Larson TR, Neumann S (2012). CAMERA: An integrated strategy for compound spectra extraction and annotation of liquid chromatography/mass spectrometry data sets. Anal Chem.

[33] Ludwig M, Dührkop K, Böcker S (2018). Bayesian networks for mass spectrometric metabolite identification via molecular fingerprints. Bioinformatics.

[34] Dührkop K, Shen H, Meusel M, Rousu J, Böcker S (2015). Searching molecular structure databases with tandem mass spectra using CSI:FingerID. Proc Nat Acad Sci USA.

[35] Allen F, Greiner R, Wishart D (2015). Competitive fragmentation modeling of ESI-MS/MS spectra for putative metabolite identification. Metabolomics.

[36] Loos M, Gerber C, Corona F, Hollender J, Singer H (2015). Accelerated isotope fine structure calculation using pruned transition trees. Anal Chem.

[37] Hohrenk LL, Itzel F, Baetz N, Tuerk J, Vosough M, Schmidt TC (2020). Comparison of software tools for liquid chromatography-high-resolution mass spectrometry data processing in nontarget screening of environmental samples. Anal Chem.

[38] Hohrenk LL, Vosough M, Schmidt TC (2019). Implementation of chemometric tools to improve data mining and prioritization in LC-HRMS for nontarget screening of organic micropollutants in complex water matrixes. Anal Chem.

[39] Myers OD, Sumner SJ, Li S, Barnes S, Du X (2017). Detailed investigation and comparison of the XCMS and MZmine 2 chromatogram construction and chromatographic peak detection methods for preprocessing mass spectrometry metabolomics data. Anal Chem.

[40] Rafiei A, Sleno L (2014). Comparison of peak-picking workflows for untargeted liquid chromatography/high-resolution mass spectrometry metabolomics data analysis. Rapid Commun Mass Spectr.

[41] Samanipour S, Baz-Lomba JA, Reid MJ, Ciceri E, Rowland S, Nilsson P, Thomas KV (2018). Assessing sample extraction efficiencies for the analysis of complex unresolved mixtures of organic pollutants: a comprehensive non-target approach. Anal Chim Acta.

[42] Samanipour S, Reid MJ, Thomas KV (2017). Statistical variable selection: an alternative prioritization strategy during the nontarget analysis of LC-HR-MS data. Anal Chem.

[43] Samanipour S, Dimitriou-Christidis P, Gros J, Grange A, Arey JS (2015). Analyte quantification with comprehensive two-dimensional gas chromatography: assessment of methods for baseline correction, peak delineation, and matrix effect elimination for real samples. J Chromatogr A.

[44] Schymanski EL, Jeon J, Gulde R, Fenner K, Ruff M, Singer HP, Hollender J (2014). Identifying small molecules via high resolution mass spectrometry: communicating confidence. Environ Sci Technol.

[45] Schymanski EL, Ruttkies C, Krauss M, Brouard C, Kind T, Dührkop K, Allen F, Vaniya A, Verdegem D, Böcker S, Rousu J, Shen H, Tsugawa H, Sajed T, Fiehn O, Ghesquière B, Neumann S (2017). Critical assessment of small molecule identification 2016: automated methods. J Cheminform.

[46] Schymanski EL, Williams AJ (2017). Open science for identifying “known unknown” chemicals. Am Chem Soc.

[47] Contributors Mc (2020) its: MassBank/MassBank-data: Release version 2020.06. 10.5281/ZENODO.3903207

[48] Kim S, Chen J, Cheng T, Gindulyte A, He J, He S, Li Q, Shoemaker BA, Thiessen PA, Yu B, Zaslavsky L, Zhang J, Bolton EE (2019). PubChem 2019 update: improved access to chemical data. Nucl Acids Res.

[49] Williams AJ, Grulke CM, Edwards J, McEachran AD, Mansouri K, Baker NC, Patlewicz G, Shah I, Wambaugh JF, Judson RS, Richard AM (2017). The CompTox chemistry dashboard: a community data resource for environmental chemistry. J Cheminf.

[50] den Uijl MJ, Schoenmakers PJ, Pirok BWJ, van Bommel MR (2021). Recent applications of retention modelling in liquid chromatography. J Sep Sci.

[51] Peng CT (2000). Prediction of retention indices: V. Influence of electronic effects and column polarity on retention index. J Chromatogr A.

[52] McEachran AD, Mansouri K, Newton SR, Beverly BEJ, Sobus JR, Williams AJ (2018). A comparison of three liquid chromatography (LC) retention time prediction models. Talanta.

[53] Kind T, Fiehn O (2010). Advances in structure elucidation of small molecules using mass spectrometry. Bioanal Rev.

[54] Vivó-Truyols G (2012). Bayesian approach for peak detection in two-dimensional chromatography. Anal Chem.

[55] Bade R, Bijlsma L, Sancho JV, Hernández F (2015). Critical evaluation of a simple retention time predictor based on LogKow as a complementary tool in the identification of emerging contaminants in water. Talanta.

[56] Noreldeen HAA, Liu X, Wang X, Fu Y, Li Z, Lu X, Zhao C, Xu G (2018). Quantitative structure-retention relationships model for retention time prediction of veterinary drugs in food matrixes. Int J Mass Spectr.

[57] Héberger K (2007). Quantitative structure-(chromatographic) retention relationships. J Chromatogr A.

[58] Vrzal T, Malečková M, Olšovská J (2021). DeepReI: deep learning-based gas chromatographic retention index predictor. Anal Chim Acta.

[59] Hall LM, Hill DW, Menikarachchi LC, Chen MH, Hall LH, Grant DF (2015). Optimizing artificial neural network models for metabolomics and systems biology: An example using HPLC retention index data. Bioanalysis.

[60] Aalizadeh R, Alygizakis NA, Schymanski EL, Krauss M, Schulze T, Ibáñez M, McEachran AD, Chao A, Williams AJ, Gago-Ferrero P, Covaci A, Moschet C, Young TM, Hollender J, Slobodnik J, Thomaidis NS (2021). Development and application of liquid chromatographic retention time indices in HRMS-based suspect and nontarget screening. Anal Chem.

[61] Wen Y, Amos RIJ, Talebi M, Szucs R, Dolan JW, Pohl CA, Haddad PR (2018). Retention index prediction using quantitative structure-retention relationships for improving structure identification in nontargeted metabolomics. Anal Chem.

[62] Amos RIJ, Haddad PR, Szucs R, Dolan JW, Pohl CA (2018). Molecular modeling and prediction accuracy in quantitative structure-retention relationship calculations for chromatography. TrAC Trends Anal Chem.

[63] Horai H, Arita M, Kanaya S, Nihei Y, Ikeda T, Suwa K, Ojima Y, Tanaka K, Tanaka S, Aoshima K, Oda Y, Kakazu Y, Kusano M, Tohge T, Matsuda F, Sawada Y, Hirai MY, Nakanishi H, Ikeda K, Akimoto N, Maoka T, Takahashi H, Ara T, Sakurai N, Suzuki H, Shibata D, Neumann S, Iida T, Tanaka K, Funatsu K, Matsuura F, Soga T, Taguchi R, Saito K, Nishioka T (2010). MassBank: a public repository for sharing mass spectral data for life sciences. J Mass Spectr.

[64] Yap CW (2011). PaDEL-descriptor: an open source software to calculate molecular descriptors and fingerprints. J Comput Chem.

[65] Consortium M MassBank EU. https://massbank.eu/MassBank/Index

[66] Heller SR, McNaught A, Pletnev I, Stein S, Tchekhovskoi D (2014). The IUPAC international chemical identifier (InChI). Chem Int Newsmag IUPAC.

[67] Lunnon WF, Brunvoll J, Cyvin SJ, Cyvin BN, Balaban AT (1988). Topological properties of benzenoid systems-the boundary code. Rev Res Fac Sci Univ Novi Sad Math Ser.

[68] Prokhorenkova L, Gusev G, Vorobev A, Dorogush AV, Gulin A (2018) Catboost: Unbiased boosting with categorical features. Advances in Neural Information Processing Systems **2018-Decem**, 6638–6648

[69] Hancock JT, Khoshgoftaar TM (2020). CatBoost for big data: an interdisciplinary review. J Big Data.

[70] Gramatica P (2007). Principles of QSAR models validation: internal and external. QSAR Combin Sci.

[71] Mason JS, Beno BR (2000). Library design using BCUT chemistry-space descriptors and multiple four-point pharmacophore fingerprints: Simultaneous optimization and structure-based diversity. J Mol Graph Modell.

