# Supplementary material for: Predicting RP-LC retention indices of structurally unknown chemicals from mass spectrometry data
Source: J Cheminform. 2023 Feb 24;15:28. doi: 10.1186/s13321-023-00699-8 (PMC9960388; doi:10.1186/s13321-023-00699-8)
Supplement: Supplementary file 1 — Additional file 1: Additional figures. [file 13321_2023_699_MOESM1_ESM.pdf]

Supplementary Information

Predicting RP-LC retention indices of structurally unknown chemicals from mass spectrometry data

Jim Boelrijk,[*,†,§] Denice van Herwerden,[‡] Bernd Ensing,[†,‖] Patrick Forré,[†,§] and Saer Samanipour[*,‡,¶,⊥]

†*AI4Science Lab, University of Amsterdam, The Netherlands*

‡*Van 't Hoff Institute for Molecular Sciences (HIMS), University of Amsterdam, Amsterdam, the Netherlands*

¶*UvA Data Science Center, University of Amsterdam, the Netherlands*

§*AMLab, Informatics Institute, University of Amsterdam, The Netherlands*

‖*Computational Chemistry Group, Van 't Hoff Institute for Molecular Sciences, University of Amsterdam, The Netherlands*

⊥*Queensland Alliance for Environmental Health Sciences (QAEHS), The University of Queensland, Woolloongabba, Australia*

E-mail: j.h.m.boelrijk@uva.nl; s.samanipour@uva.nl

Contents

S1. Leverage distributions **3**

S1.1. Descriptor based model 3

S1.2. Cumulative Neutral Losses (CNL) based model 4

S2. Prinicipal component analysis **5**

S3. Hyperparameter selection **7**

S4. Distributions of important features **8**

S4.1. Descriptor based model 8

S4.2. Cumulative Neutral Losses (CNL) based model 28

S5. Interpretation of Selected Descriptors for Descriptor Based model **54**

S6. Interpretation of Selected CNLs for CNL Based model **55**

S1. Leverage distributions

This section contains figures of the distributions of leverages in the training and test sets of descriptor to retention index (Section S1.1.) and the neutral losses to retention index model (Section S1.2.).

S1.1. Descriptor based model

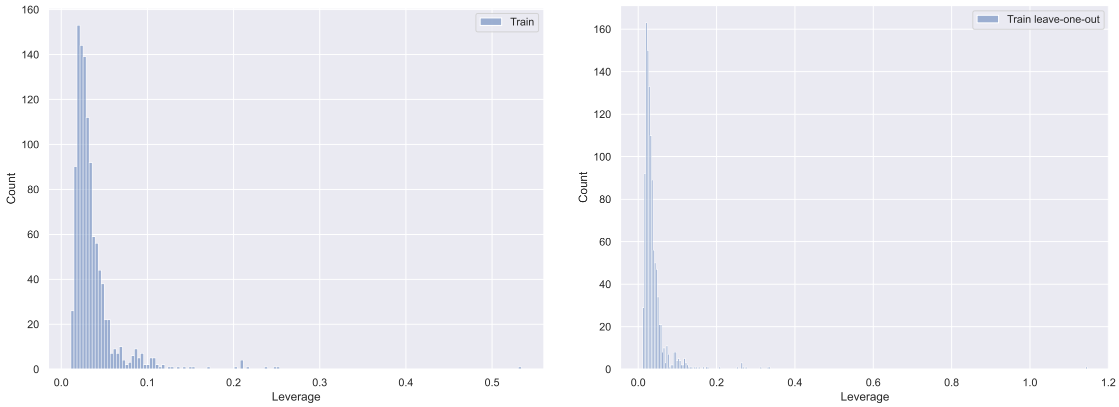

Figure 1: Leverage distribution of the training set (left) and the training set with leverages computed using a leave-one-out approach (right)

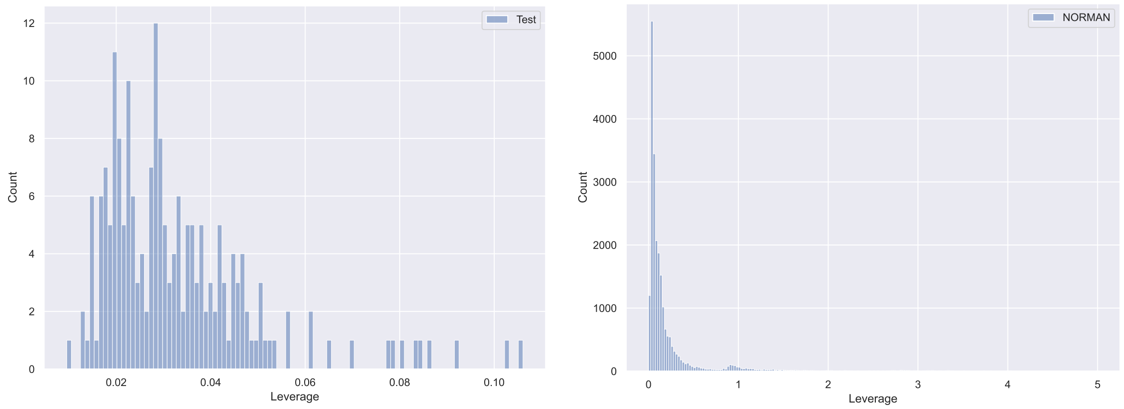

Figure 2: Leverage distributions of the test set (left) and NORMAN dataset (right)

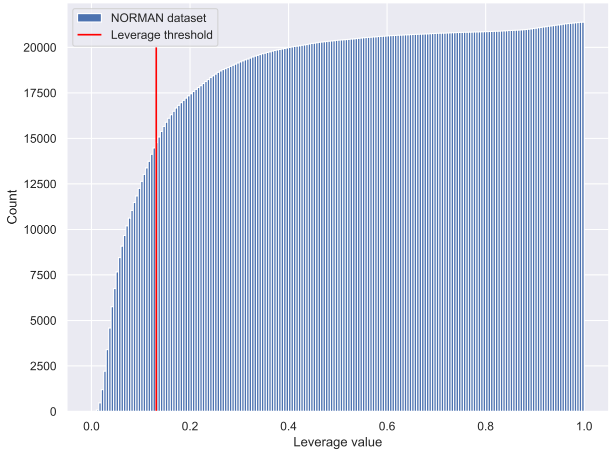

Figure 3: Cumulative distribution of the Norman dataset. With the red vertical line denoting the acceptable leverage threshold. All compound on the left of the red vertical line can be reliably used for prediction.

S1.2. Cumulative Neutral Losses (CNL) based model

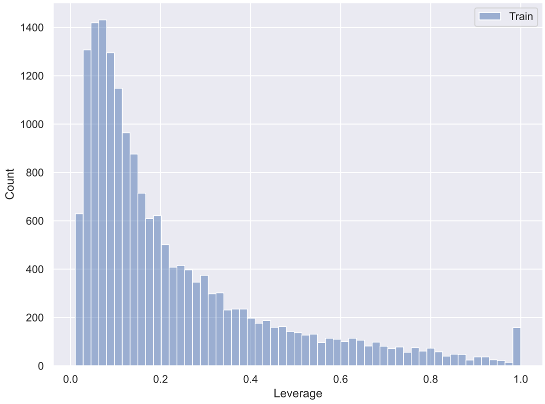

Figure 4: Leverage distribution of the training set.

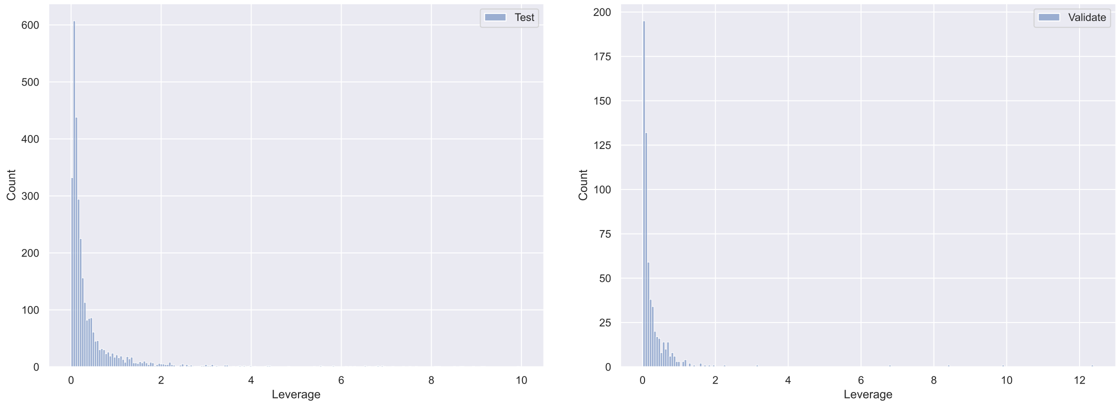

Figure 5: Leverage distribution of the test set (left) and the external validation set (right)

S2. Principal component analysis

To further assess the chemical similarity of the Amide and NORMAN datasets, besides the leverage calculations, we performed principal component analysis. This was done to determine if we could reliably use the descriptor based model trained on the Amide dataset to predict retention indices for the NORMAN dataset. Which in turn then could be used as additional training data for the CNL model.

PCA was performed on a concatenated dataset which contained all datapoints from both the Amide and NORMAN dataset. Next all features were standardized onto unit scale (mean=0 and variance=1). Then PCA was performed to project the descriptor data onto 2 principal components (i.e. dimensions). In addition, it was checked if a third principal component would not separate the two distributions.

This was done using only the 40 descriptors that were used in the final descriptor based model and is shown in Figure 6 and also using all stable descriptors as is shown in Figure 7. Here it can be seen that a large fraction of the Norman dataset overlaps with the Amide dataset. However, the Amide dataset does not fully describe the Norman dataset. This provides similar insights as was shown in the leverage analysis.

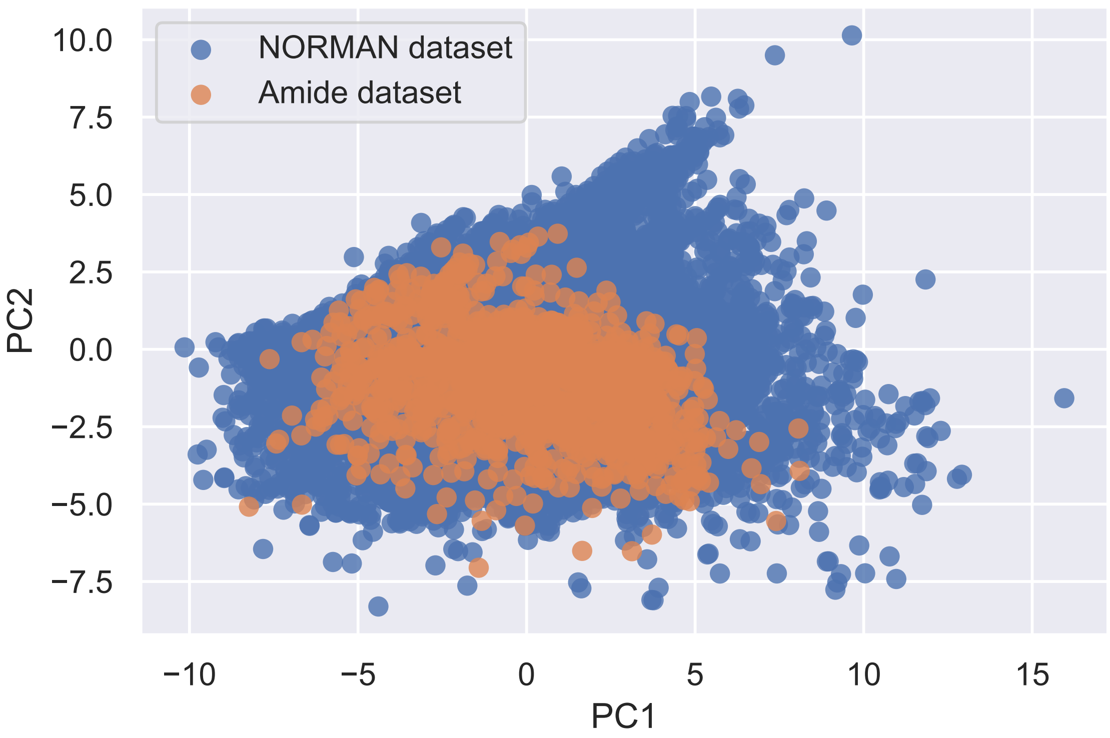

Figure 6: PCA on only the 40 descriptors used by the model.

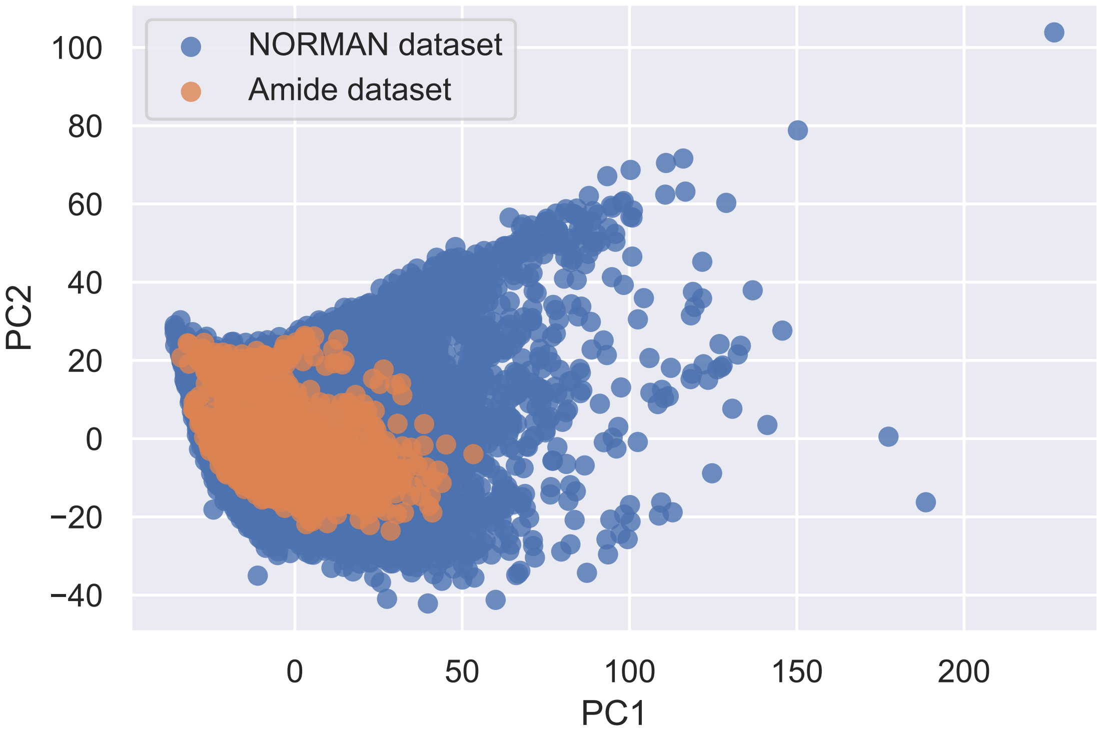

Figure 7: PCA on all model descriptors.

S3. Hyperparameter selection

Here we provide additional information regarding the hyperparameters we chose for the models described in Section 3.2 of the main text. In general, CatBoost is known to provide good model performance without parameter tuning (thus using their default parameters), as is is shown at ref. [1] for a plethora of datasets. Yet the developers provide general advice to perform parameter tuning [2] for the most important parameters. Both regarding their impact on model performance and what viable ranges are for these parameters. Based on this advice and model training time we performed our hyperparameter selection. For the descriptor based model, we performed a grid search over a parameter grid of depth=[4,6,8]

[1] https://catboost.ai/#benchmark
[2] https://catboost.ai/en/docs/concepts/parameter-tuning

and L2 leaf regularization of [1, 5, 10], from which the best model was picked. By using the early stopping, i.e., stopping training if the error on the validation set did not decrease for more than 5 iterations, we prevented overfitting.

For the CNL model, training time took considerably longer (in the range of 12 hours), which was deemed too long to perform an extensive hyper parameter search. Here we manually tested combinations of learning rate and the number of trees. Generally, a lower learning rate prevents overfitting, but requires more iterations and thus longer training times and better model expectations. As the CNL dataset contains considerably more features, it was believed that the CNL model would require more iterations, therefore, we kept the learning rate fixed at an acceptable value, we iteratively increased the training iterations if no overfitting occurred on the last iterations (the training did not yet converge). Other model parameters were kept to the default value.

We note that all the parameters of our model can be found (in the model object) on our Github page [3].

S4. Distributions of important features

S4.1. Descriptor based model

This section contains figures of the distributions of the descriptors used by the descriptor based model described in the main text, for both the amide and Norman dataset, and labeled as such.

[3] https://github.com/Jimbo994/NL2RI

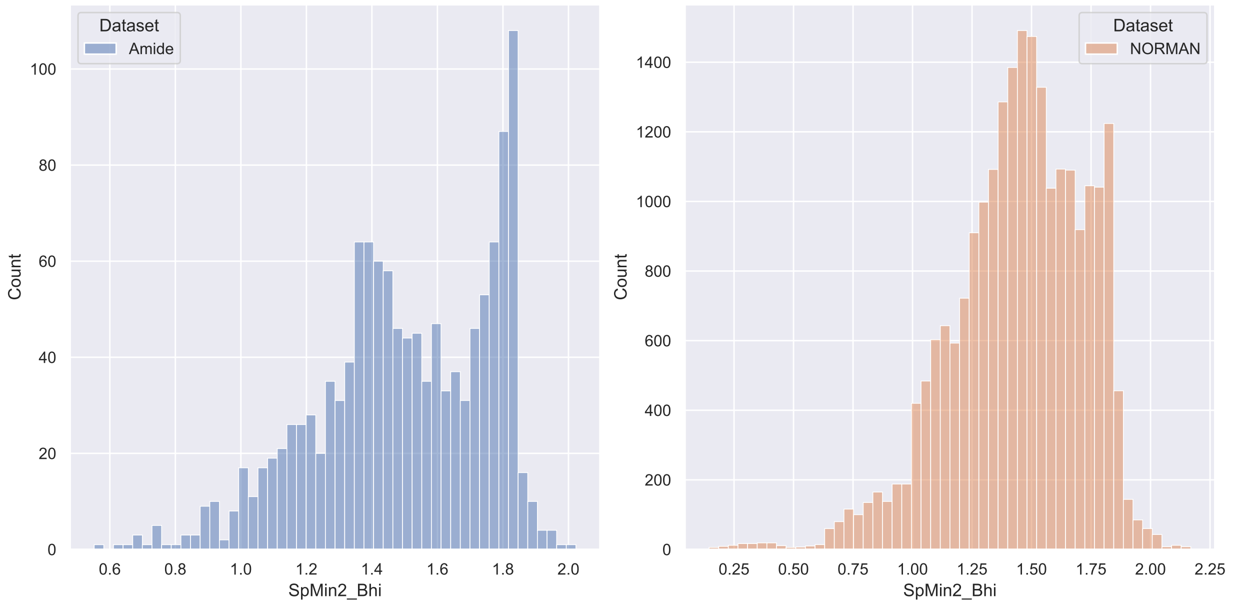

Figure 8: Distribution of SpMin2_Bhi descriptor for Amide dataset (left) and Norman dataset (right).

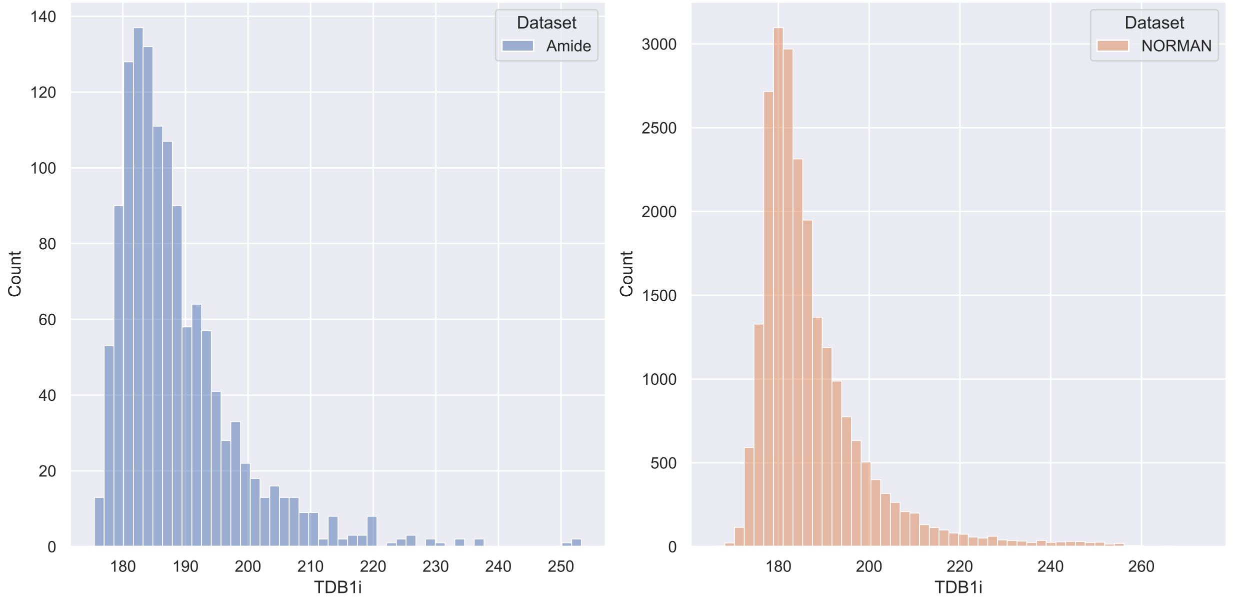

Figure 9: Distribution of TDB1i descriptor for Amide dataset (left) and Norman dataset (right).

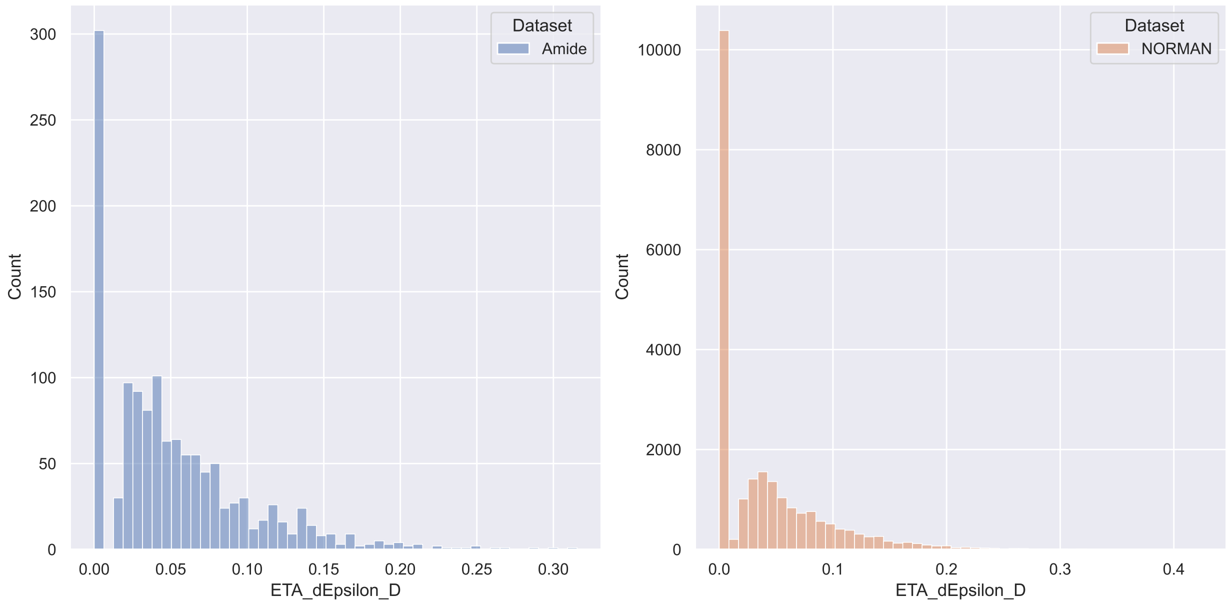

Figure 10: Distribution of ETA_dEpsilon_D descriptor for Amide dataset (left) and Norman dataset (right).

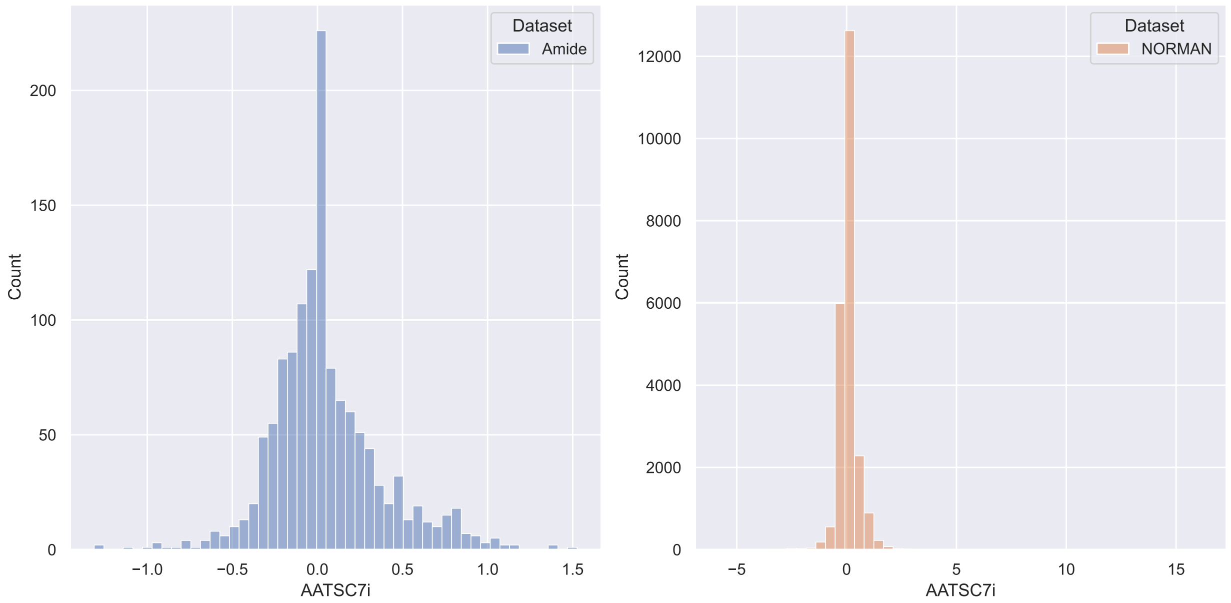

Figure 11: Distribution of AATSC7i descriptor for Amide dataset (left) and Norman dataset (right).

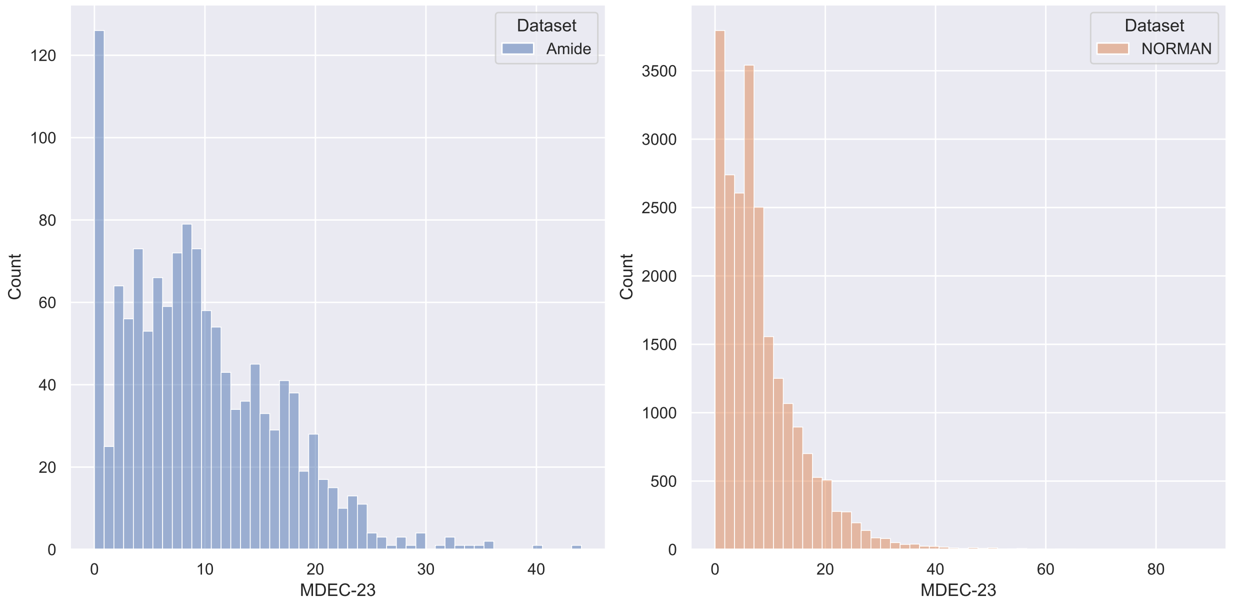

Figure 12: Distribution of MDEC-23 descriptor for Amide dataset (left) and Norman dataset (right).

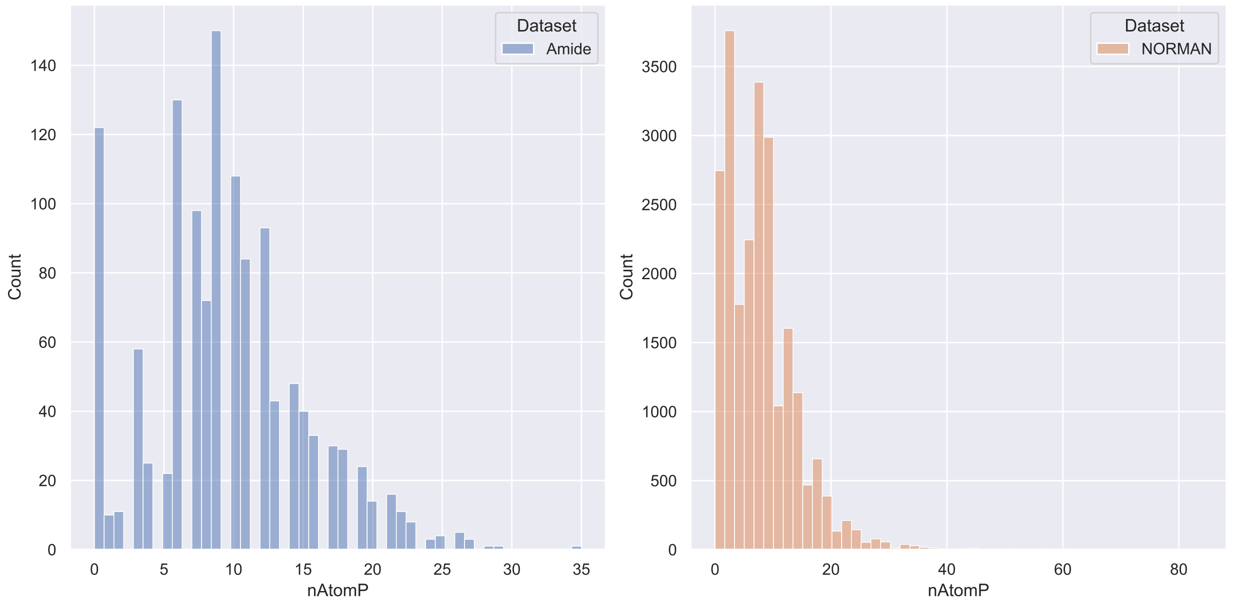

Figure 13: Distribution of nAtomP descriptor for Amide dataset (left) and Norman dataset (right).

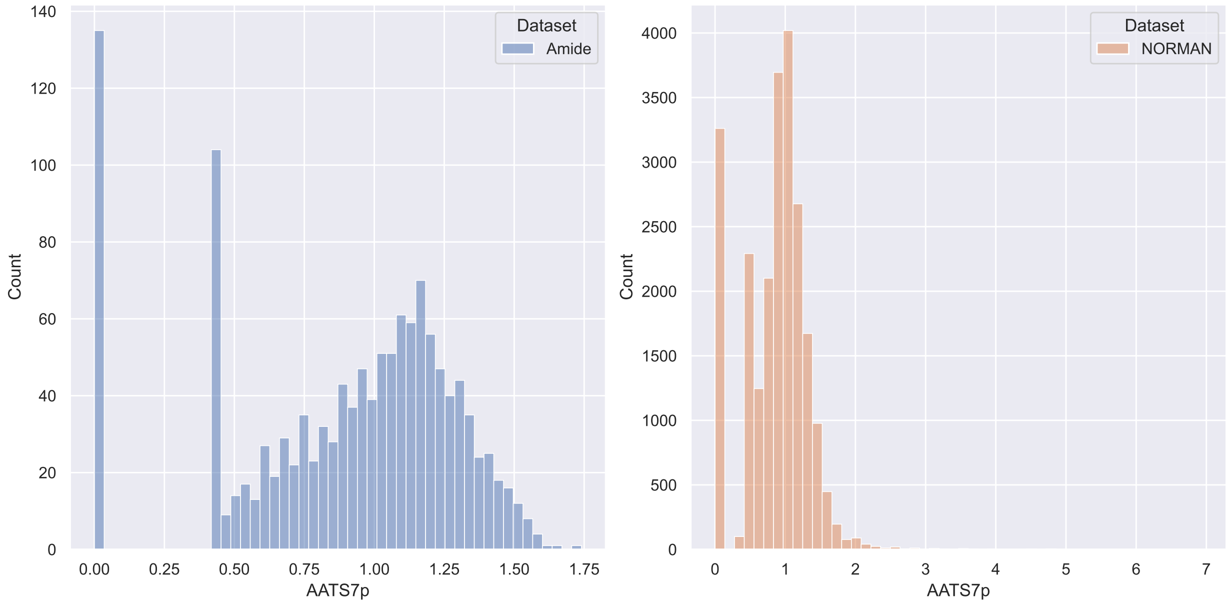

Figure 14: Distribution of AATS7p descriptor for Amide dataset (left) and Norman dataset (right).

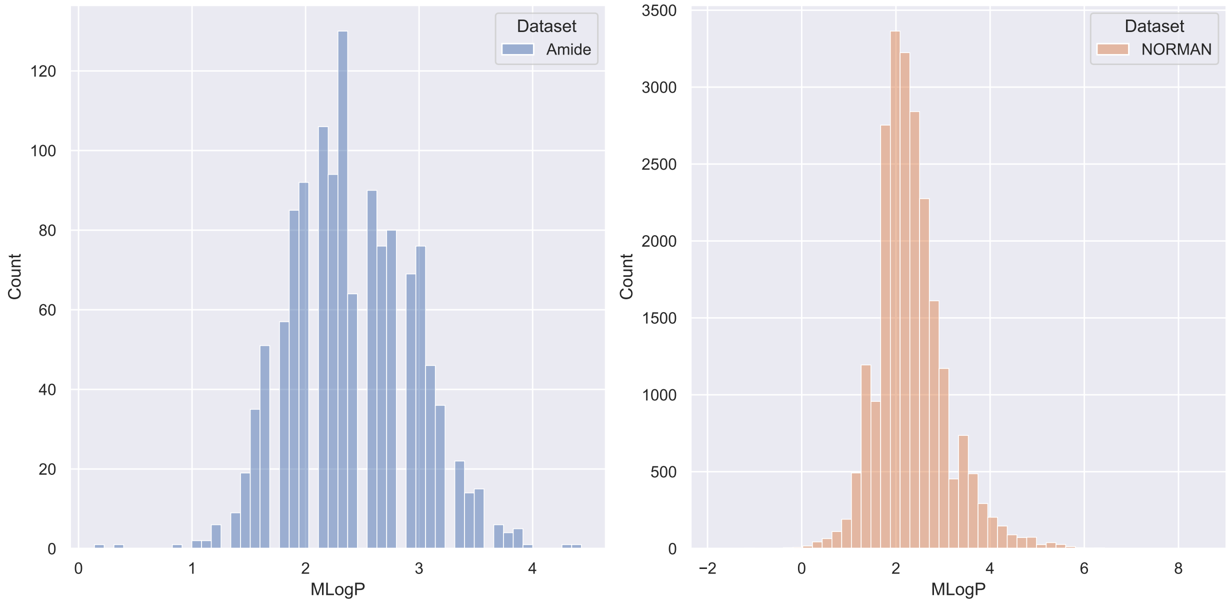

Figure 15: Distribution of MLogP descriptor for Amide dataset (left) and Norman dataset (right).

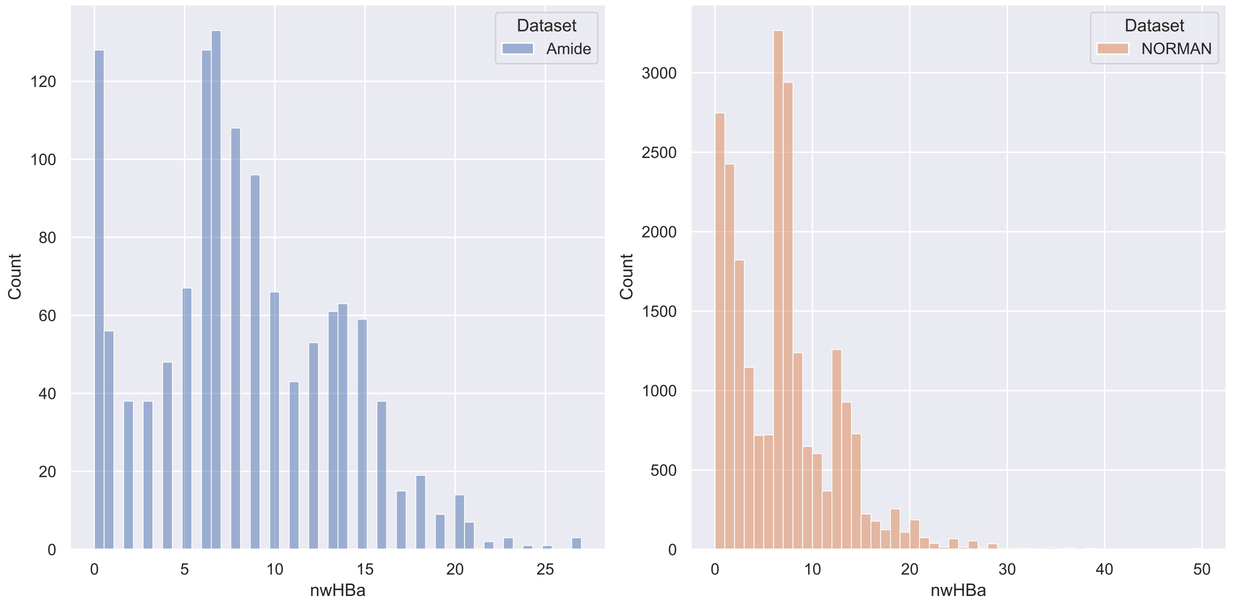

Figure 16: Distribution of nwHBa descriptor for Amide dataset (left) and Norman dataset (right).

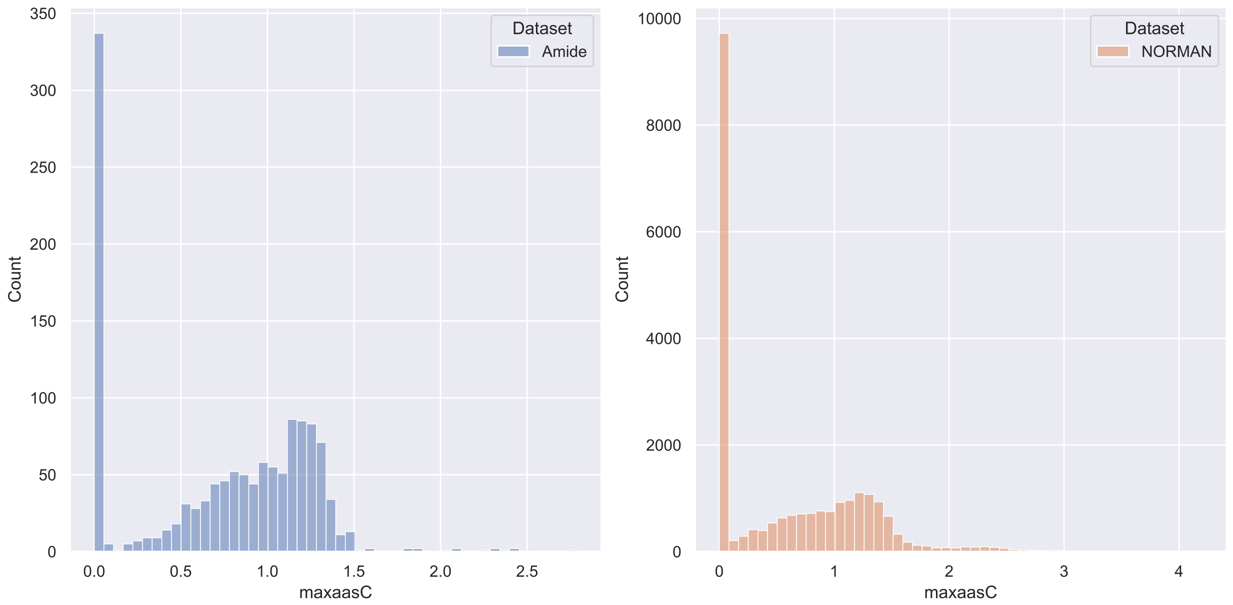

Figure 17: Distribution of maxaasC descriptor for Amide dataset (left) and Norman dataset (right).

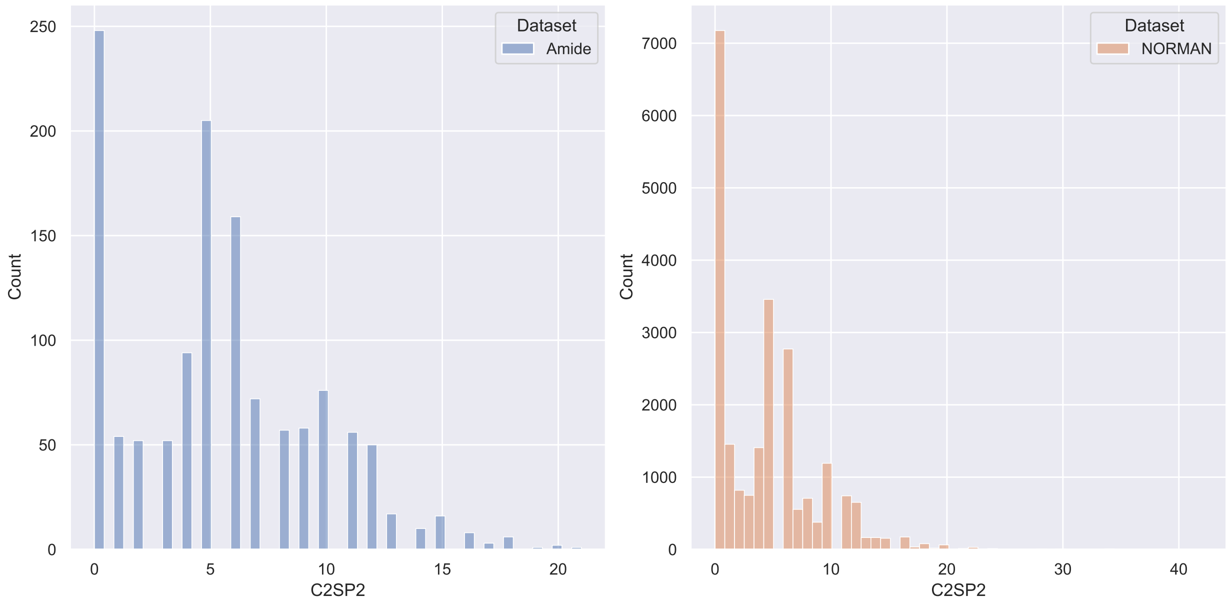

Figure 18: Distribution of C2SP2 descriptor for Amide dataset (left) and Norman dataset (right).

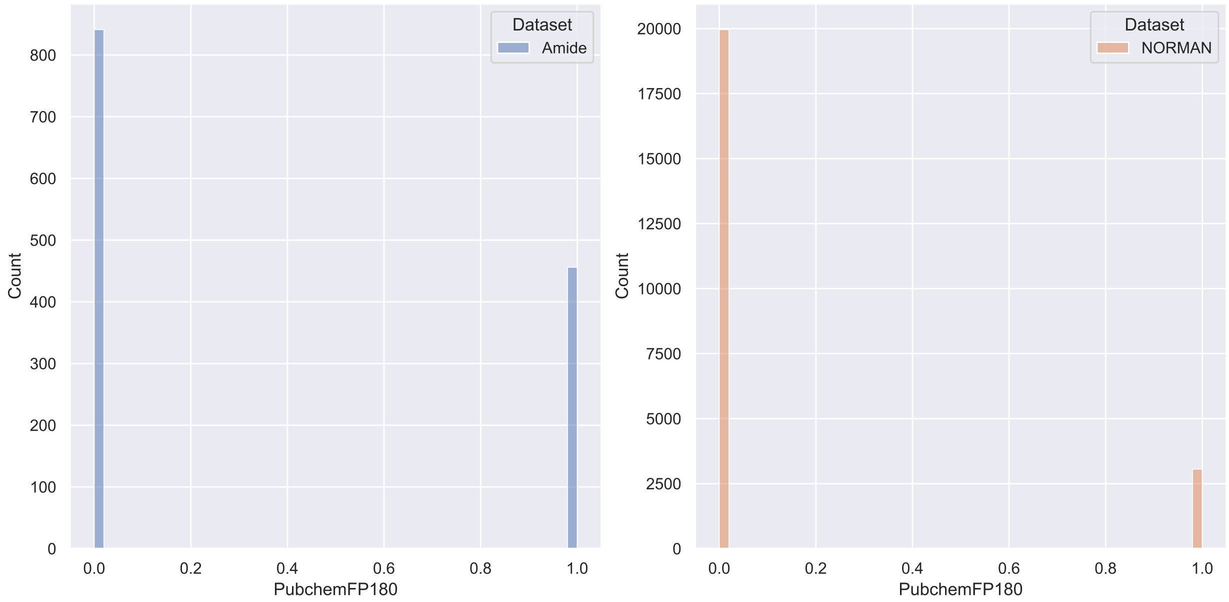

Figure 19: Distribution of PubchemFP180 descriptor for Amide dataset (left) and Norman dataset (right).

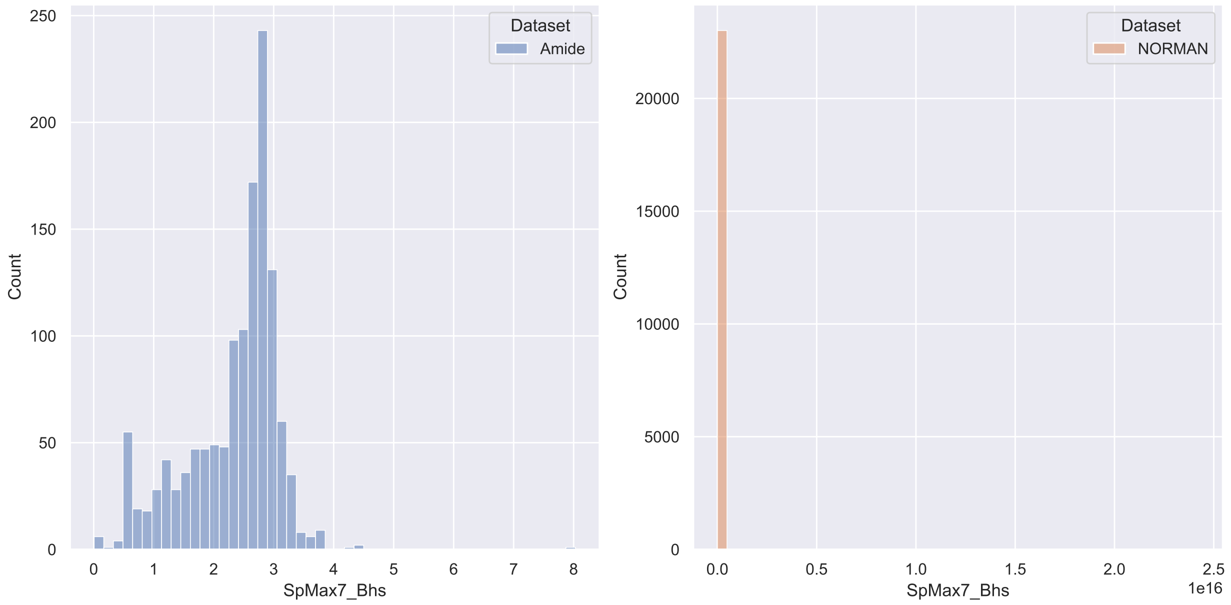

Figure 20: Distribution of SpMax7_Bhs descriptor for Amide dataset (left) and Norman dataset (right).

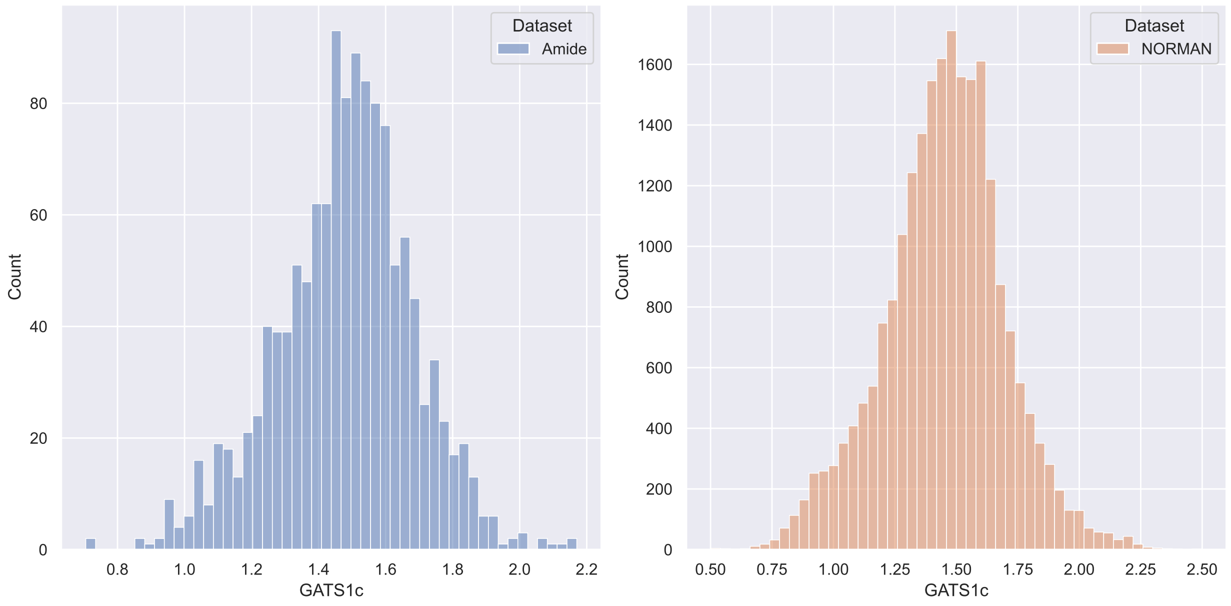

Figure 21: Distribution of GATS1c descriptor for Amide dataset (left) and Norman dataset (right).

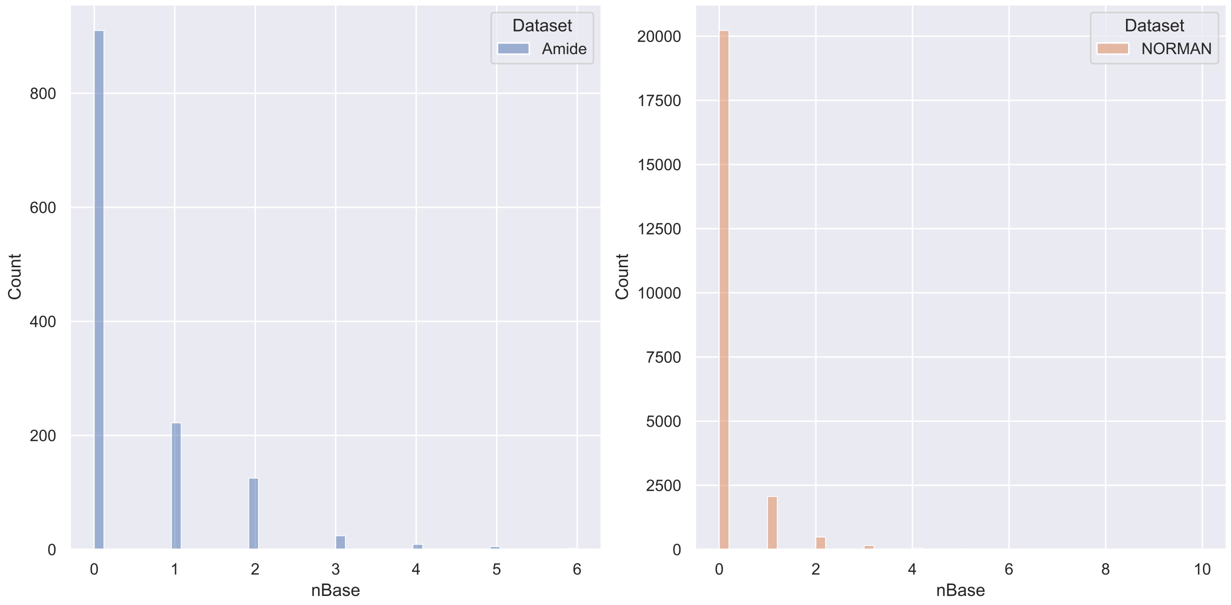

Figure 22: Distribution of nBase descriptor for Amide dataset (left) and Norman dataset (right).

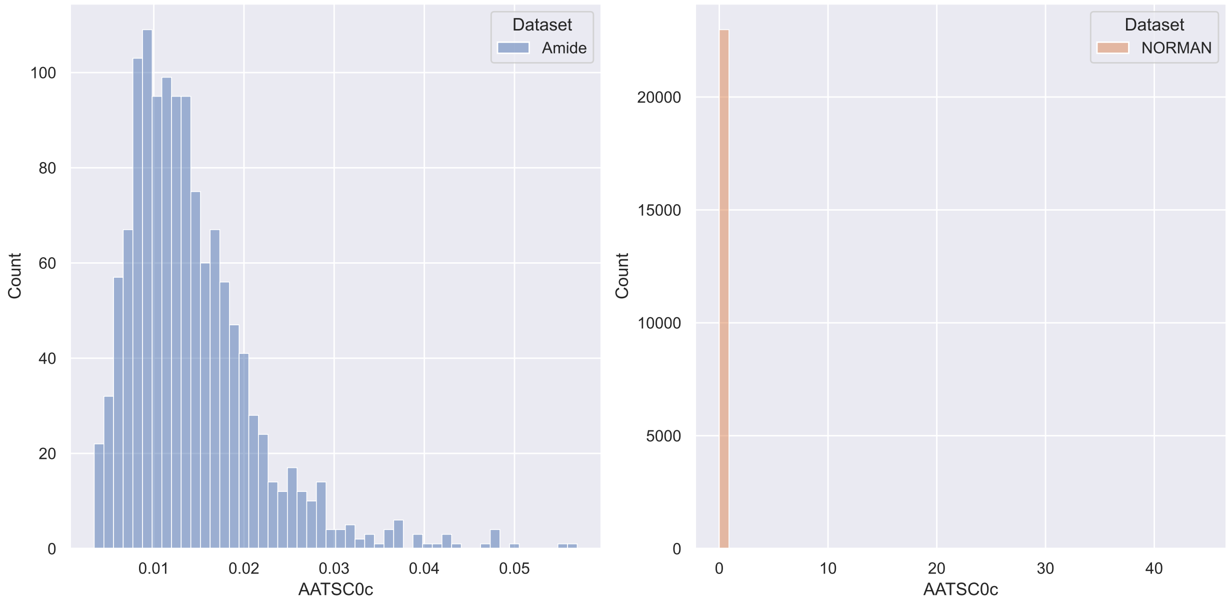

Figure 23: Distribution of AATSC0c descriptor for Amide dataset (left) and Norman dataset (right).

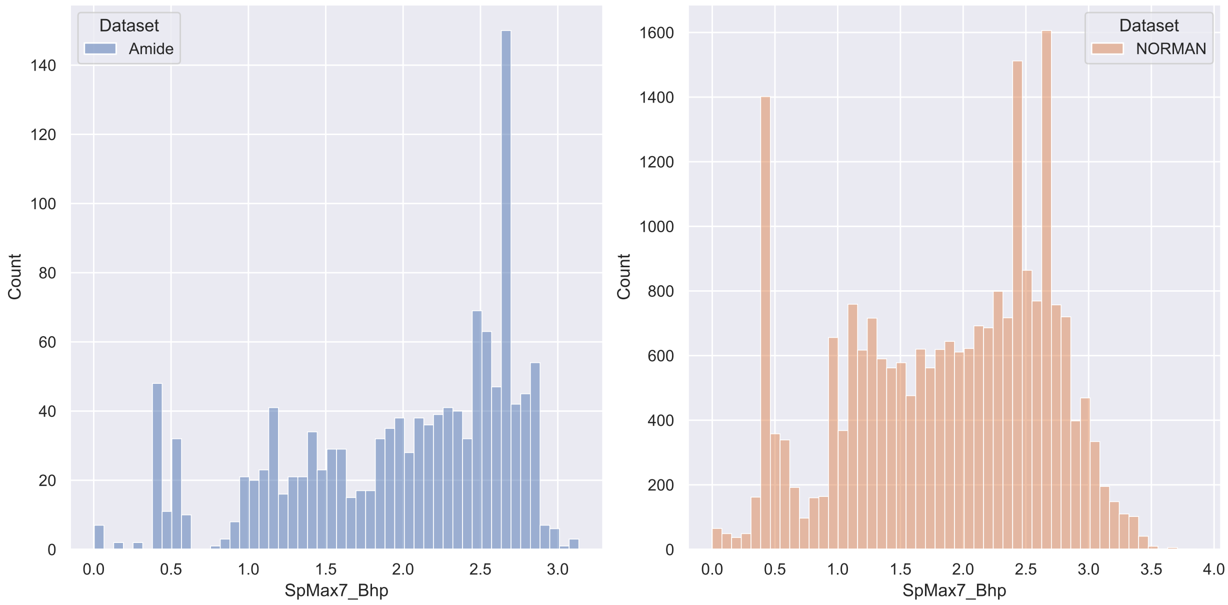

Figure 24: Distribution of SpMax7_Bhp descriptor for Amide dataset (left) and Norman dataset (right).

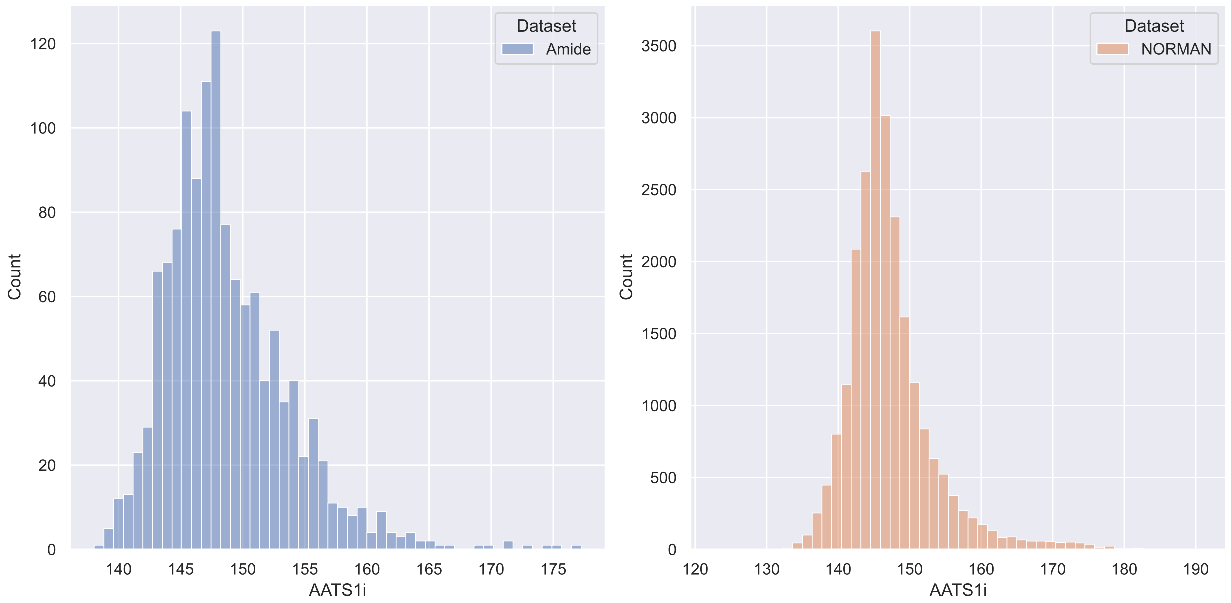

Figure 25: Distribution of AATS1i descriptor for Amide dataset (left) and Norman dataset (right).

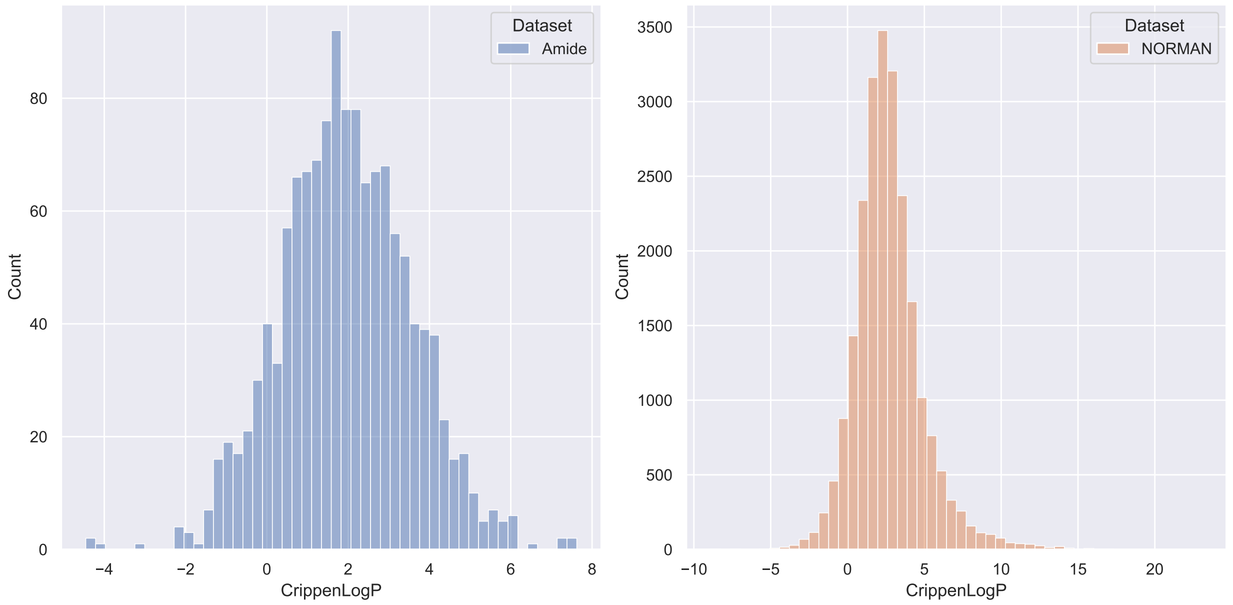

Figure 26: Distribution of CrippenLogP descriptor for Amide dataset (left) and Norman dataset (right).

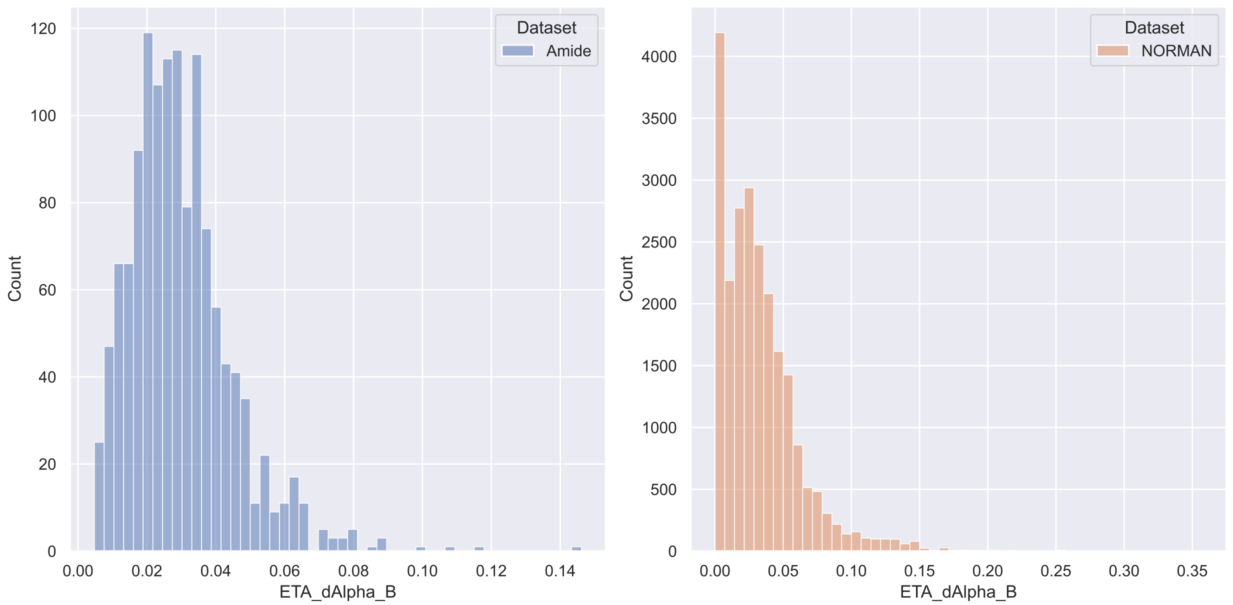

Figure 27: Distribution of ETA_dAlpha_B descriptor for Amide dataset (left) and Norman dataset (right).

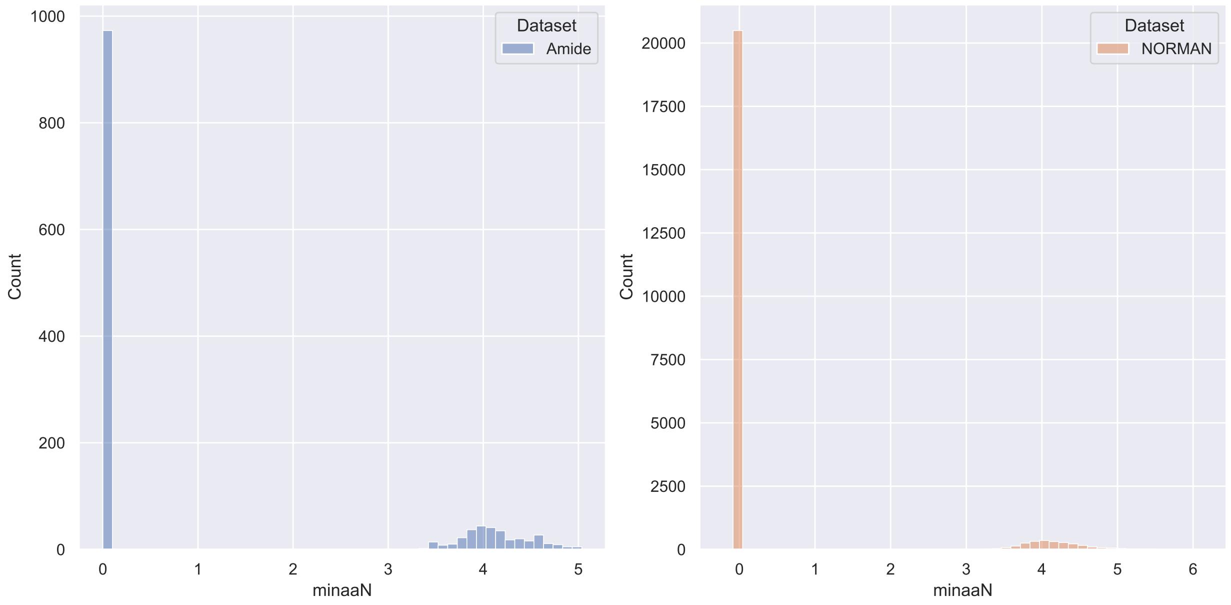

Figure 28: Distribution of minaaN descriptor for Amide dataset (left) and Norman dataset (right).

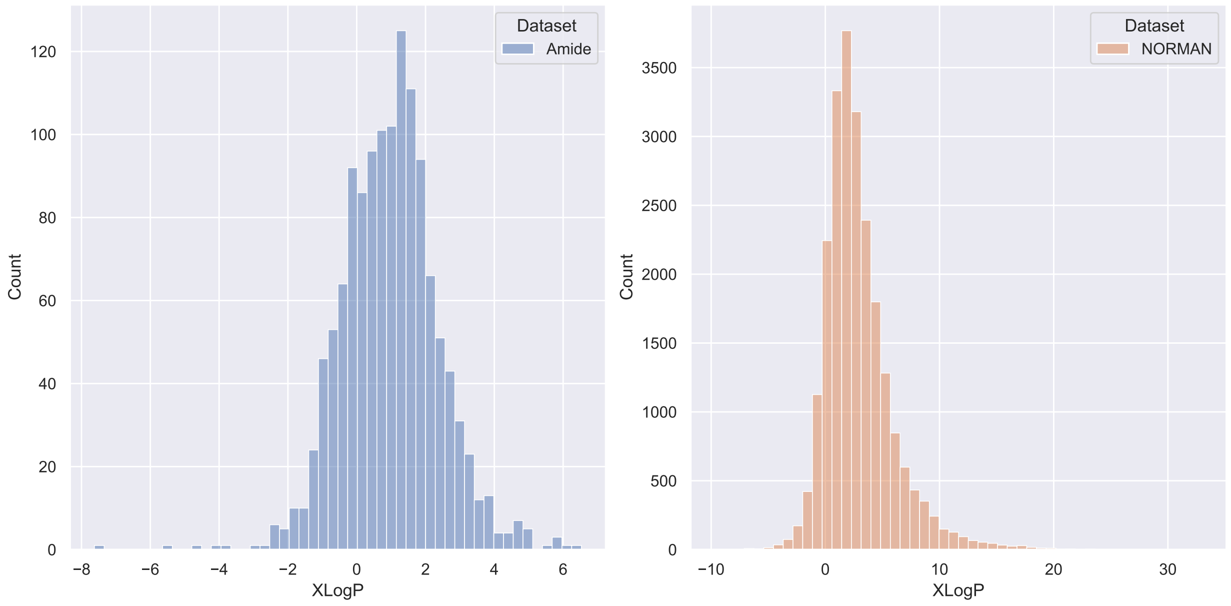

Figure 29: Distribution of XLogP descriptor for Amide dataset (left) and Norman dataset (right).

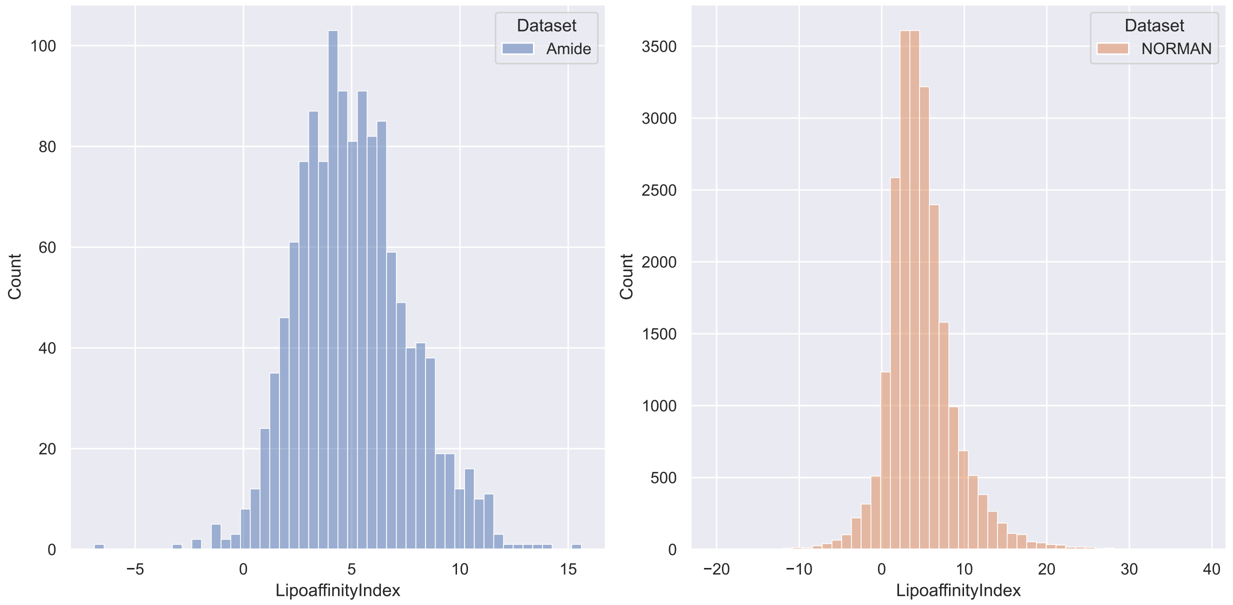

Figure 30: Distribution of LipoaffinityIndex descriptor for Amide dataset (left) and Norman dataset (right).

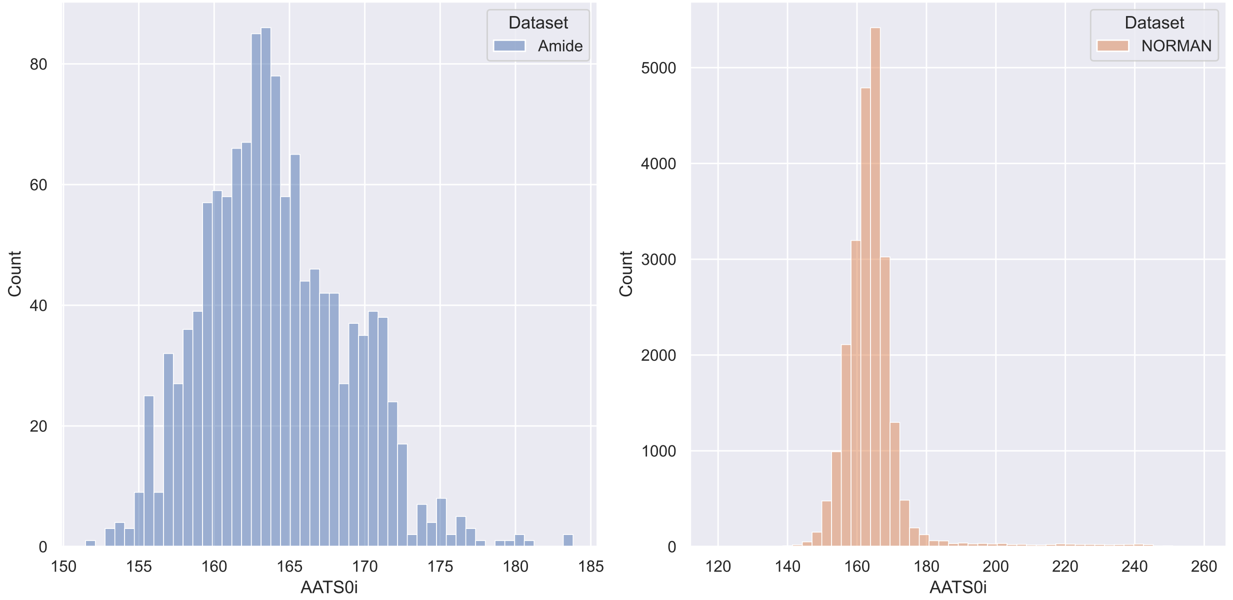

Figure 31: Distribution of AATS0i descriptor for Amide dataset (left) and Norman dataset (right).

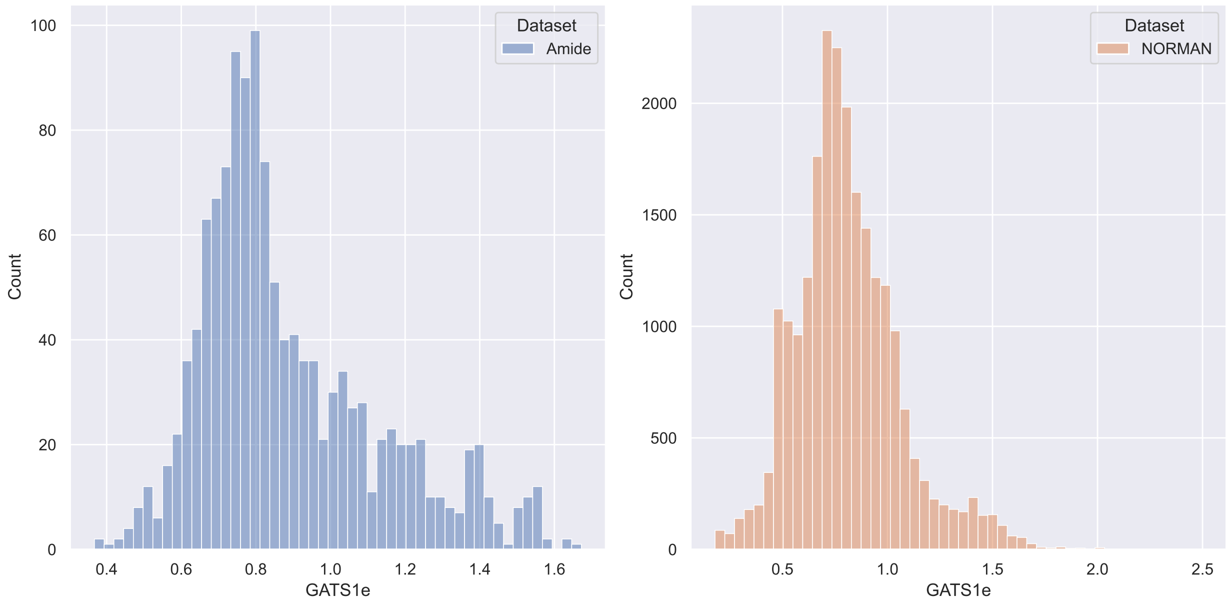

Figure 32: Distribution of GATS1e descriptor for Amide dataset (left) and Norman dataset (right).

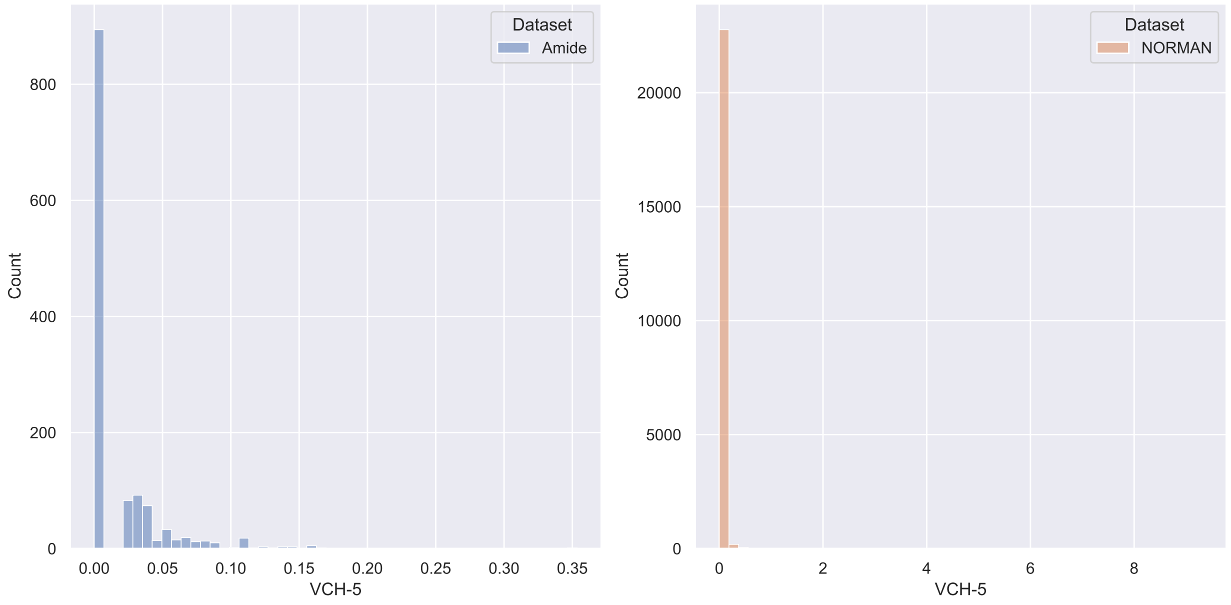

Figure 33: Distribution of VCH-5 descriptor for Amide dataset (left) and Norman dataset (right).

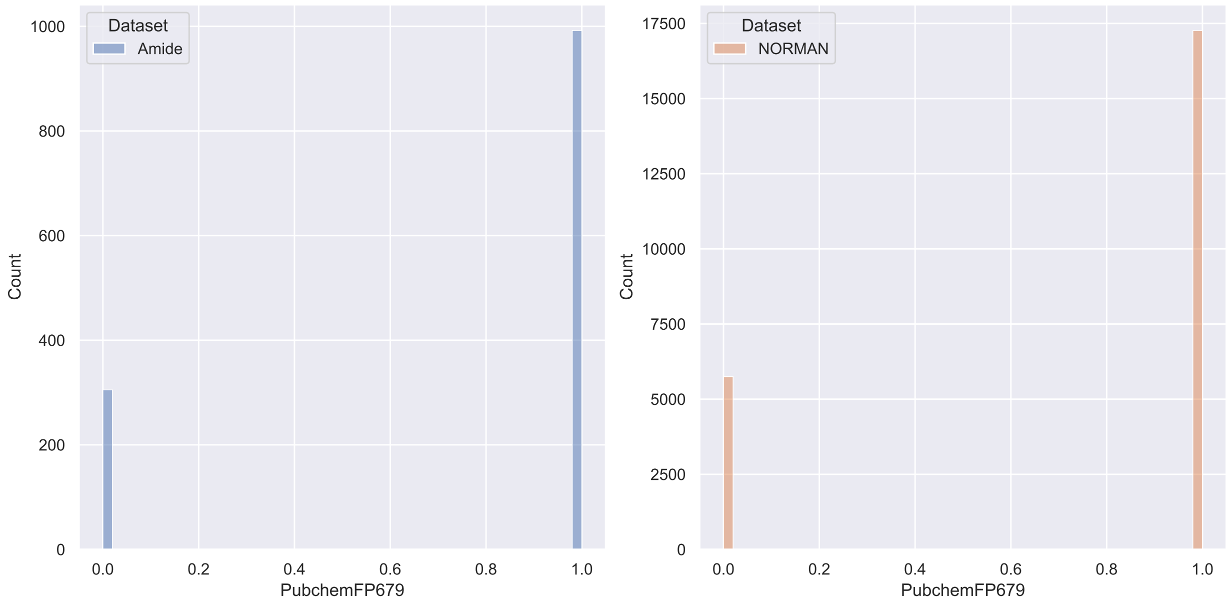

Figure 34: Distribution of PubchemFP679 descriptor for Amide dataset (left) and Norman dataset (right).

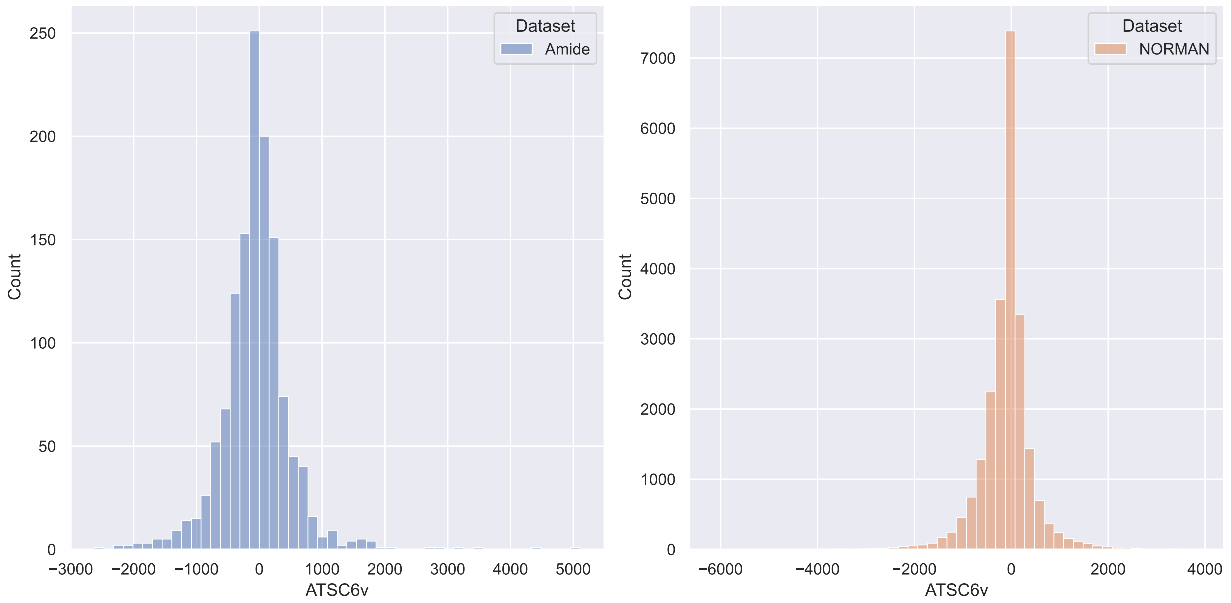

Figure 35: Distribution of ATSC6v descriptor for Amide dataset (left) and Norman dataset (right).

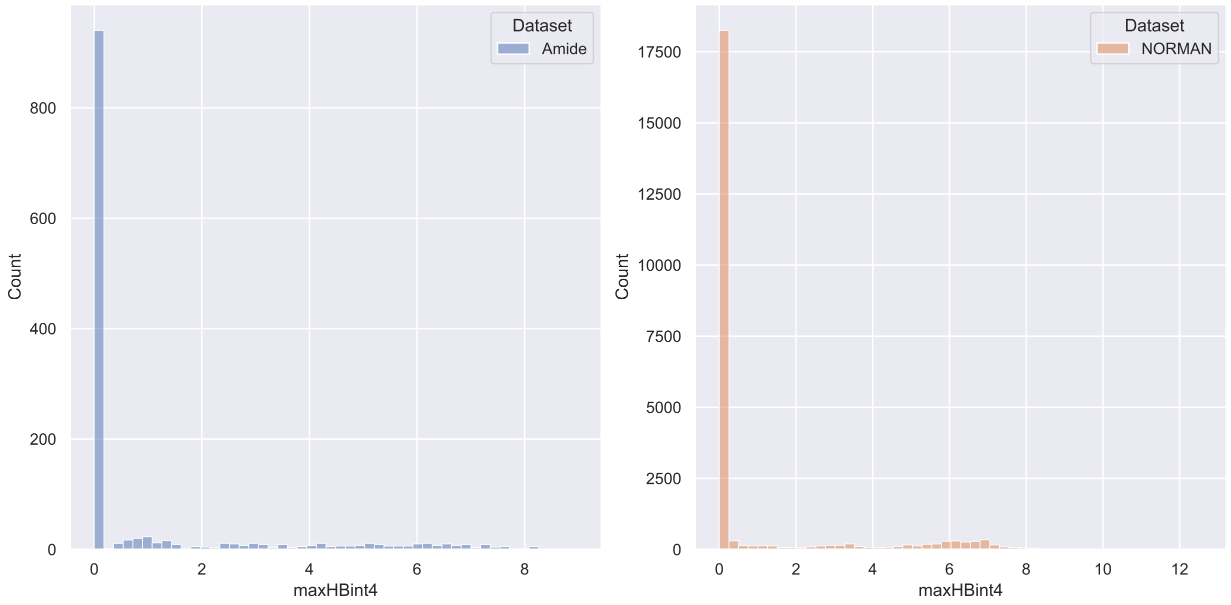

Figure 36: Distribution of maxHBint4 descriptor for Amide dataset (left) and Norman dataset (right).

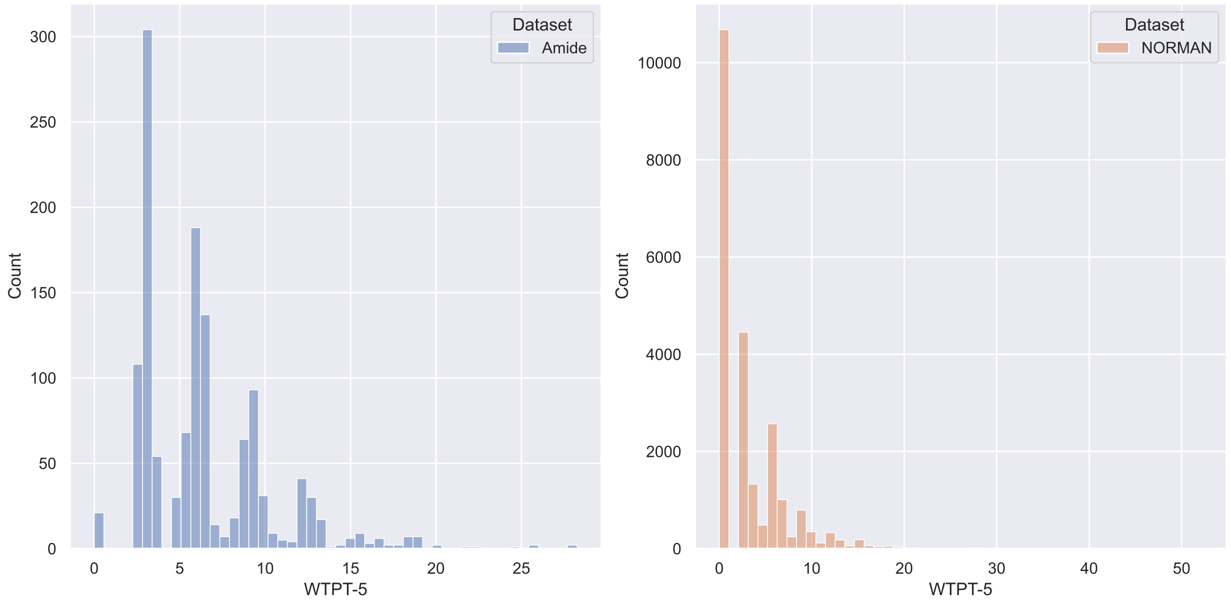

Figure 37: Distribution of WTPT-5 descriptor for Amide dataset (left) and Norman dataset (right).

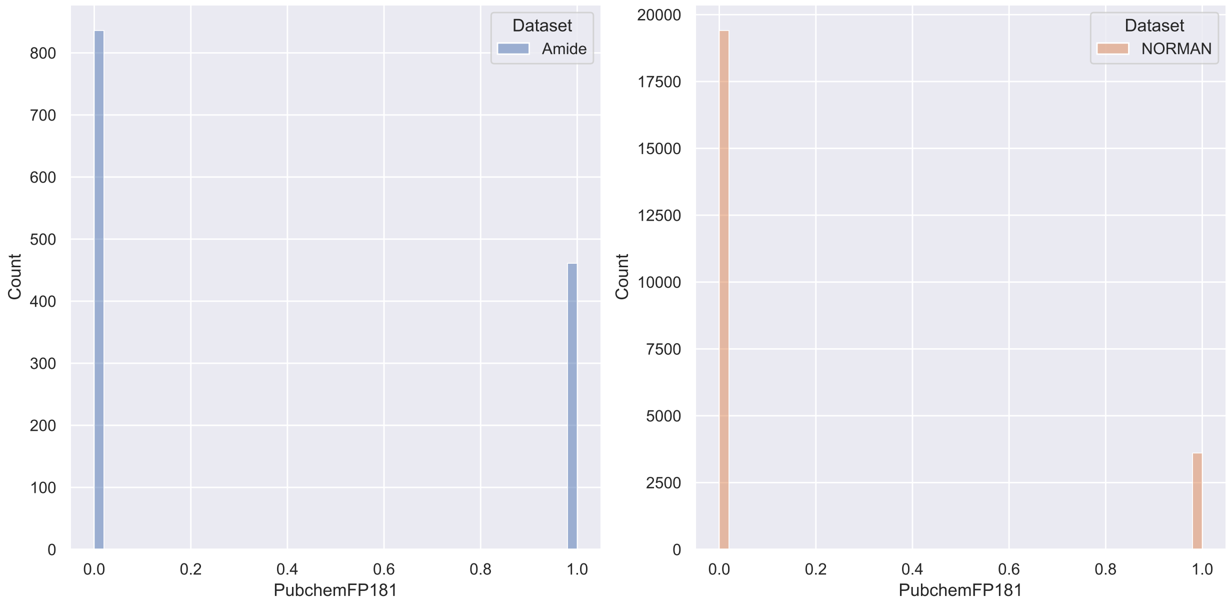

Figure 38: Distribution of PubchemFP181 descriptor for Amide dataset (left) and Norman dataset (right).

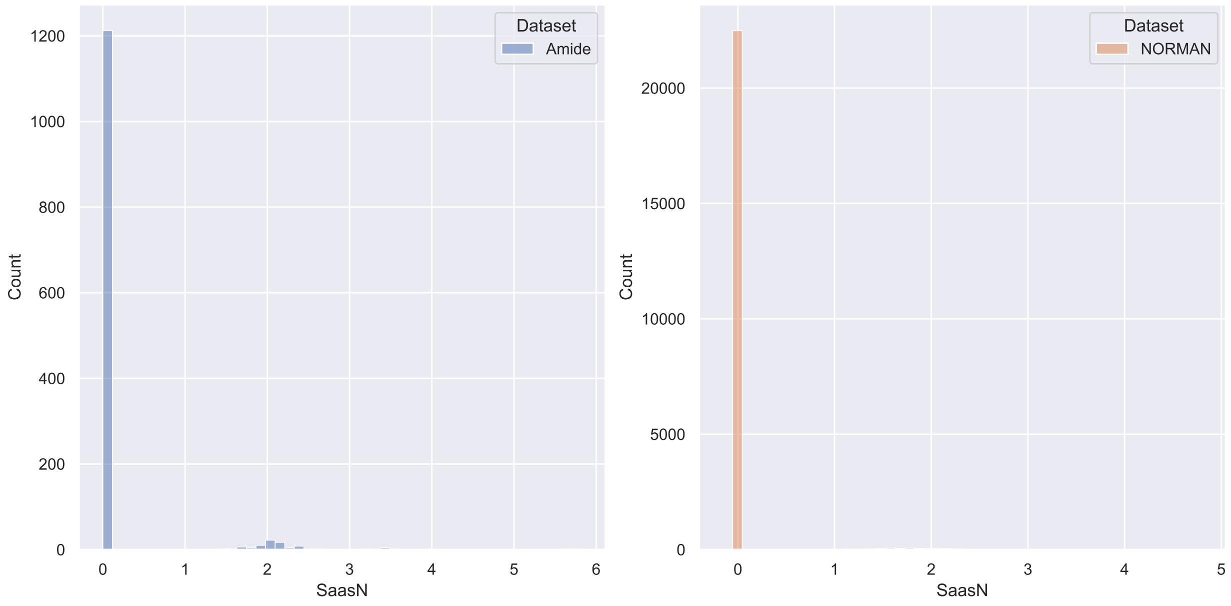

Figure 39: Distribution of SaasN descriptor for Amide dataset (left) and Norman dataset (right).

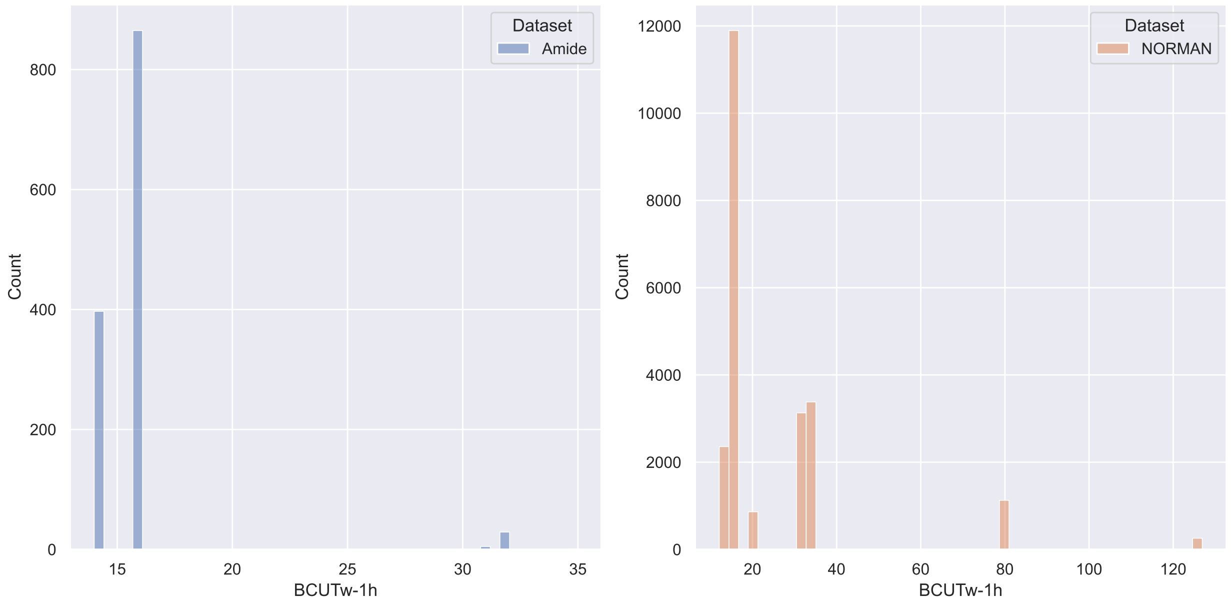

Figure 40: Distribution of BCUTw-1h descriptor for Amide dataset (left) and Norman dataset (right).

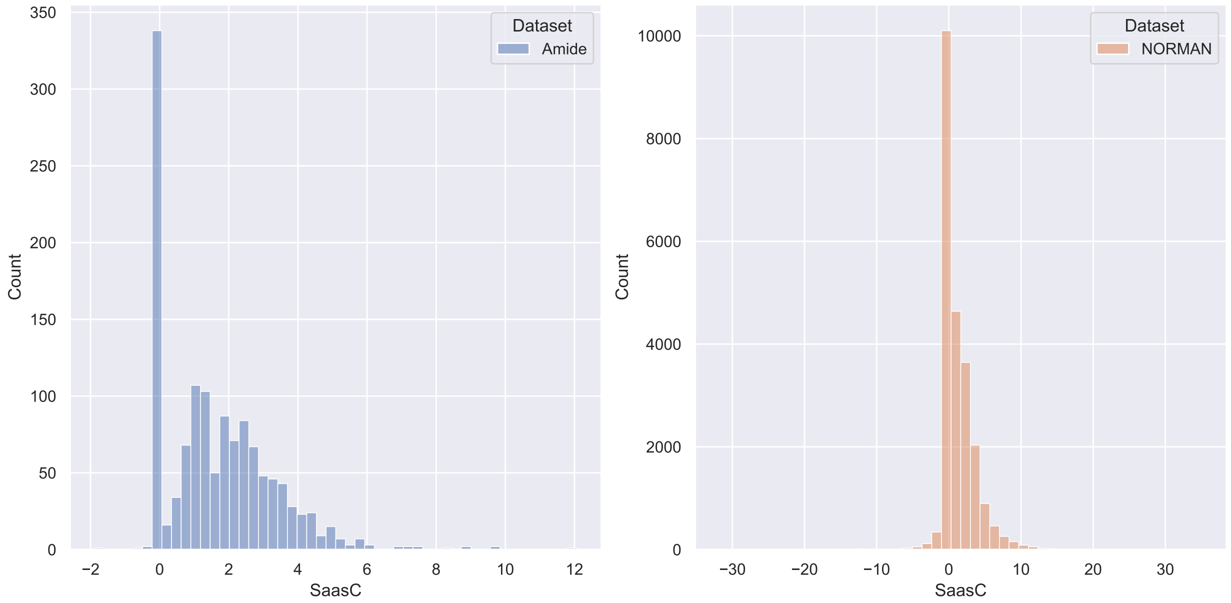

Figure 41: Distribution of SaasC descriptor for Amide dataset (left) and Norman dataset (right).

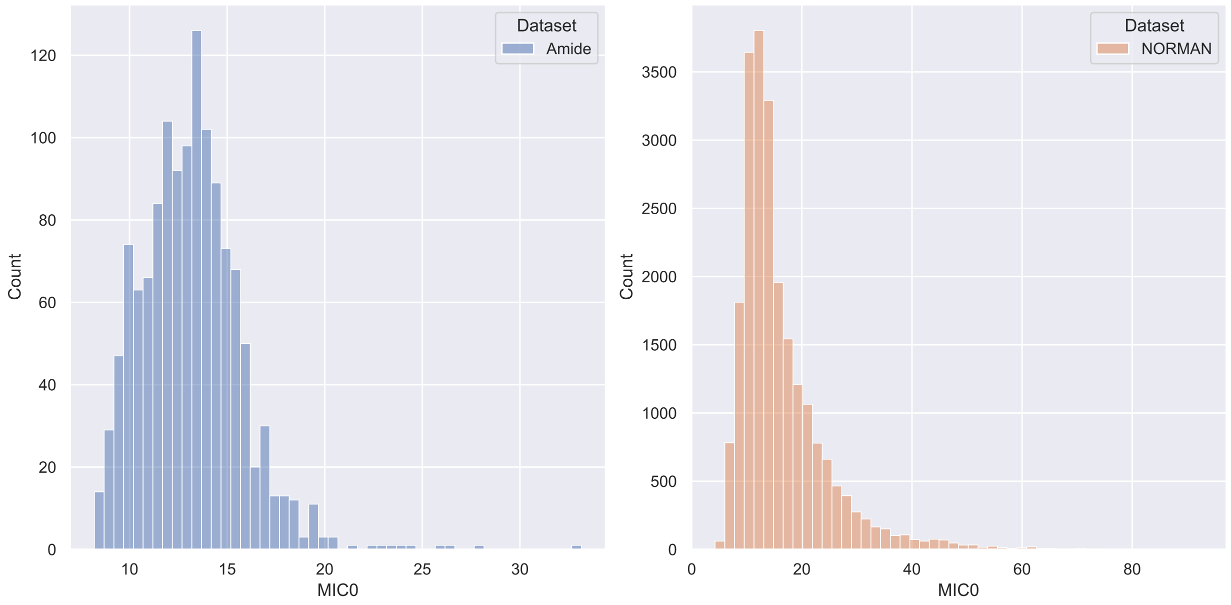

Figure 42: Distribution of MIC0 descriptor for Amide dataset (left) and Norman dataset (right).

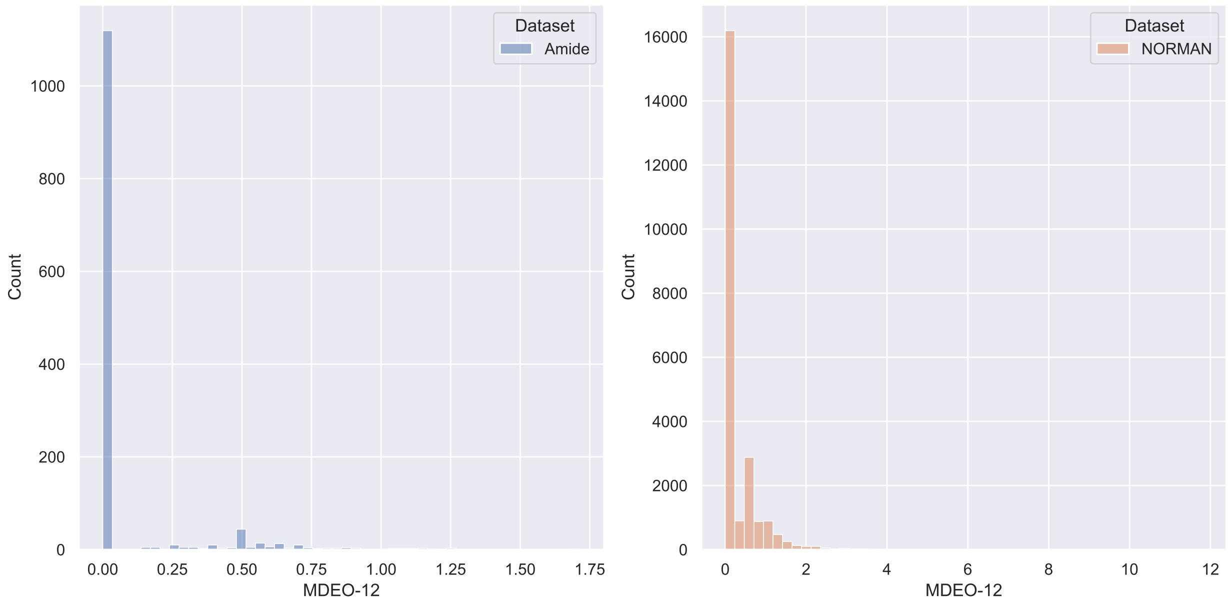

Figure 43: Distribution of MDEO-12 descriptor for Amide dataset (left) and Norman dataset (right).

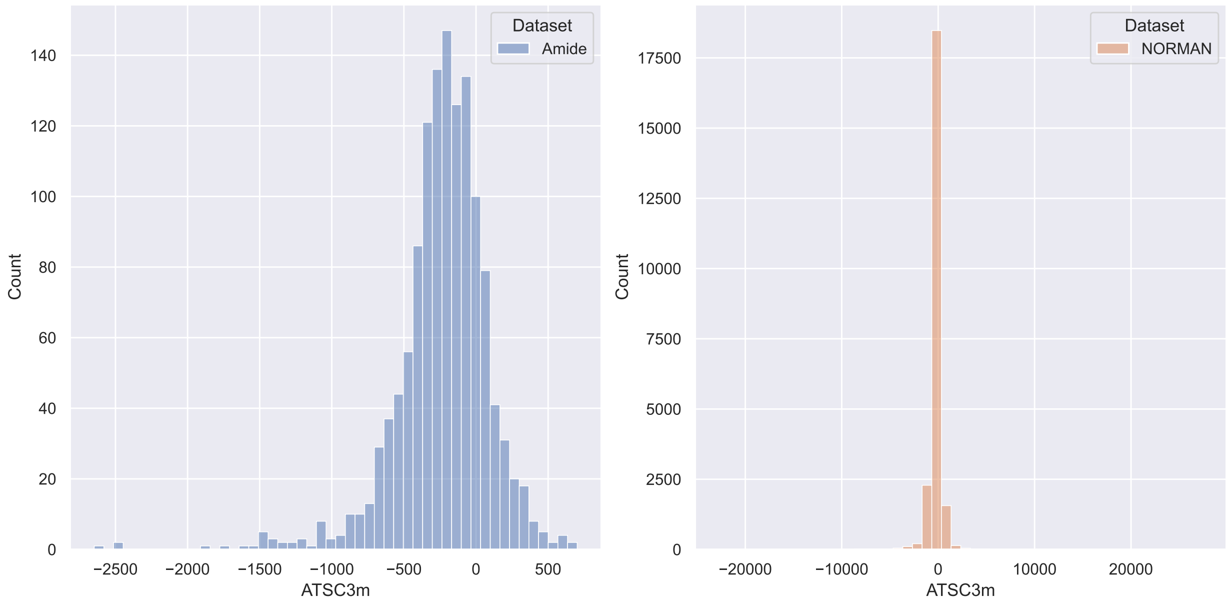

Figure 44: Distribution of ATSC3m descriptor for Amide dataset (left) and Norman dataset (right).

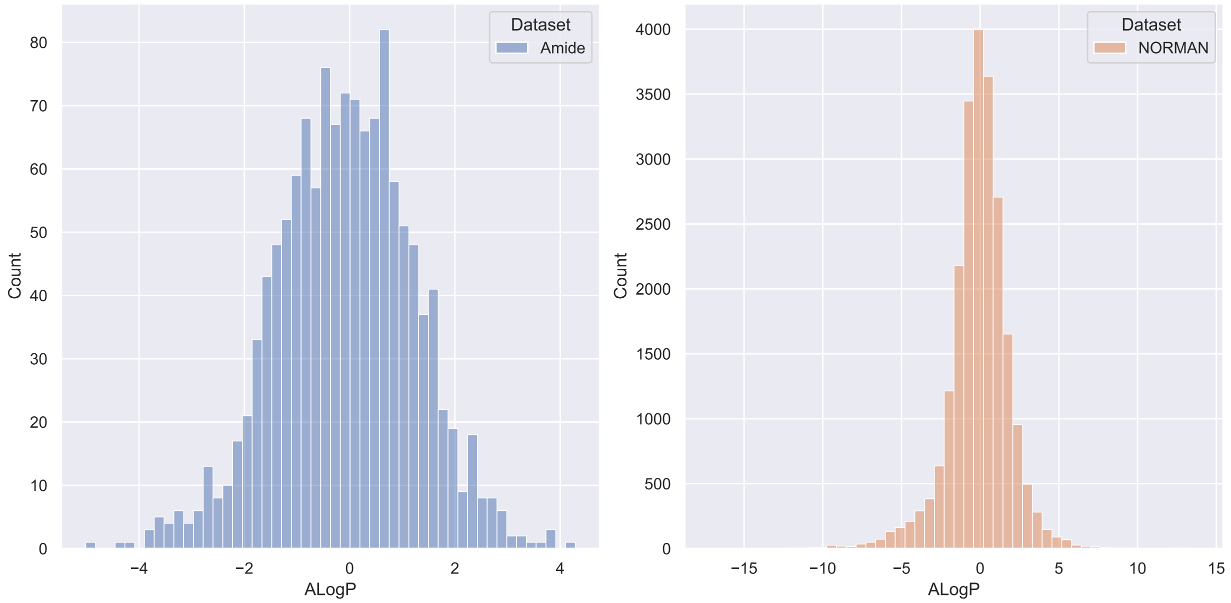

Figure 45: Distribution of ALogP descriptor for Amide dataset (left) and Norman dataset (right).

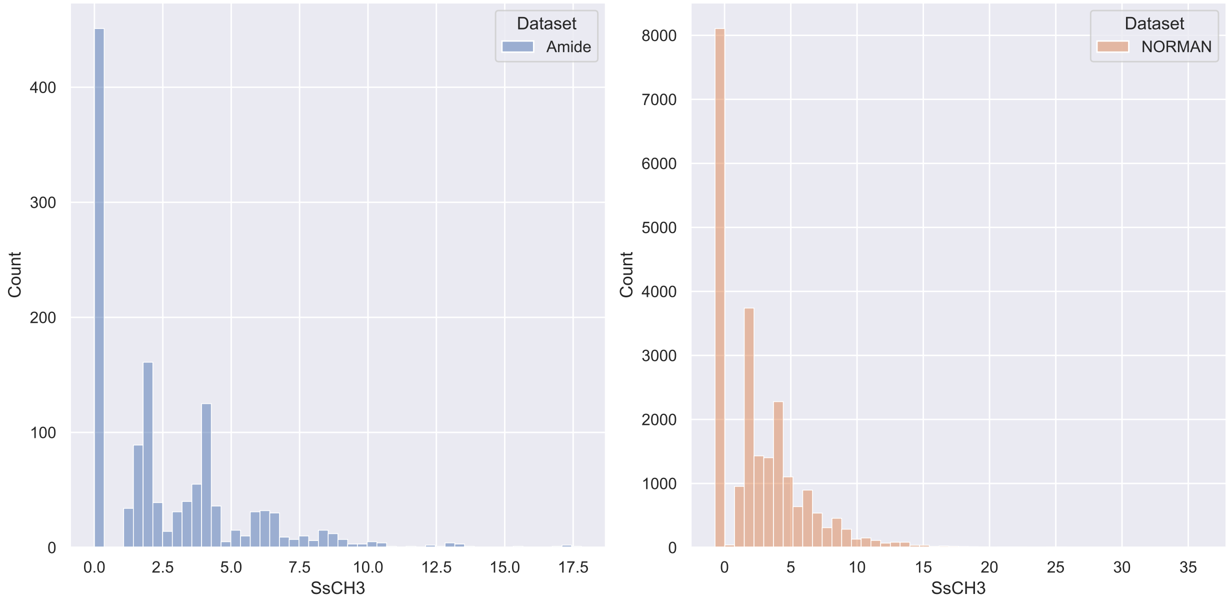

Figure 46: Distribution of SsCH3 descriptor for Amide dataset (left) and Norman dataset (right).

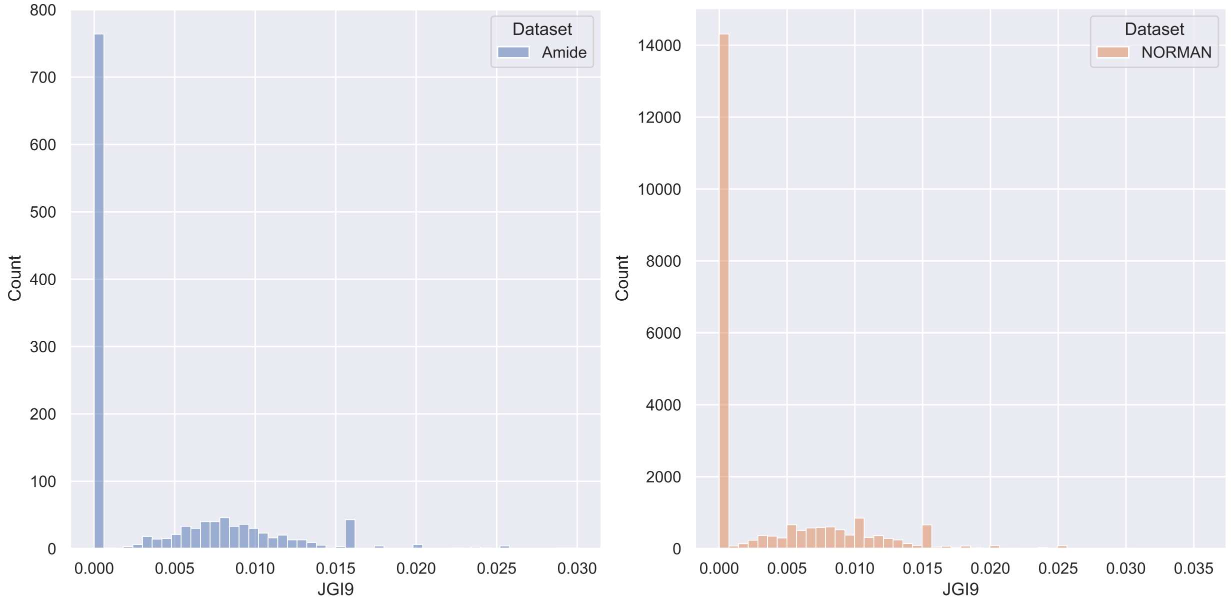

Figure 47: Distribution of JGI9 descriptor for Amide dataset (left) and Norman dataset (right).

S4.2. Cumulative Neutral Losses (CNL) based model

This section contains figures of the distributions of the 50 most important CNLS of the CNL based model described in the main text, for both the amide and Norman dataset, and labeled as such.

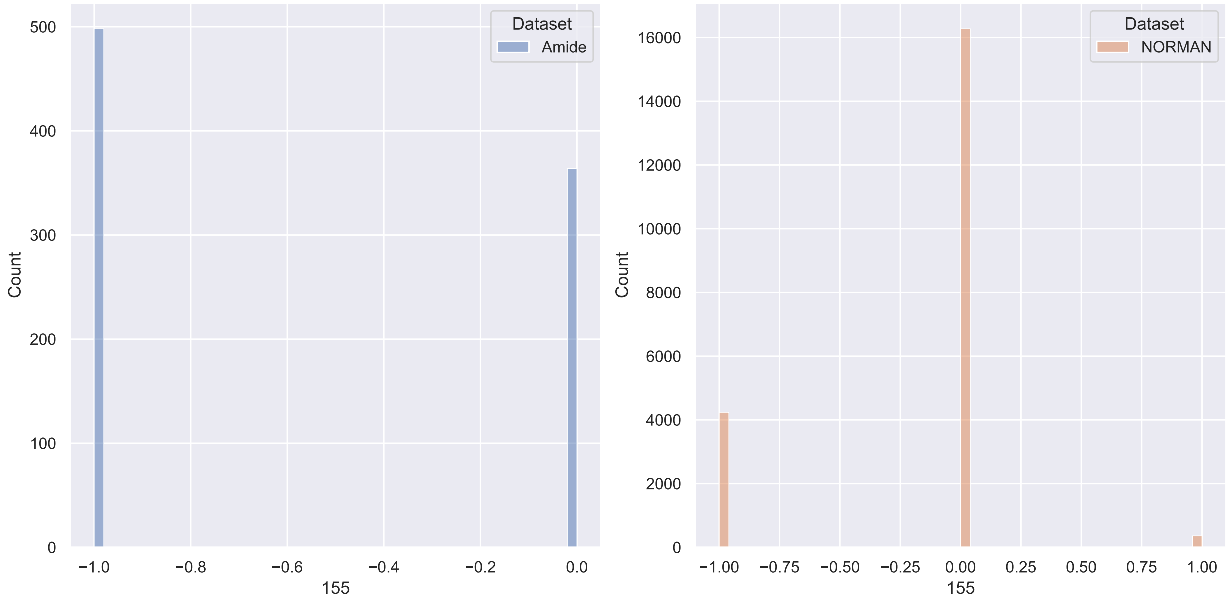

Figure 48: Distribution of neutral loss 155 for Amide dataset (left) and Norman dataset (right).

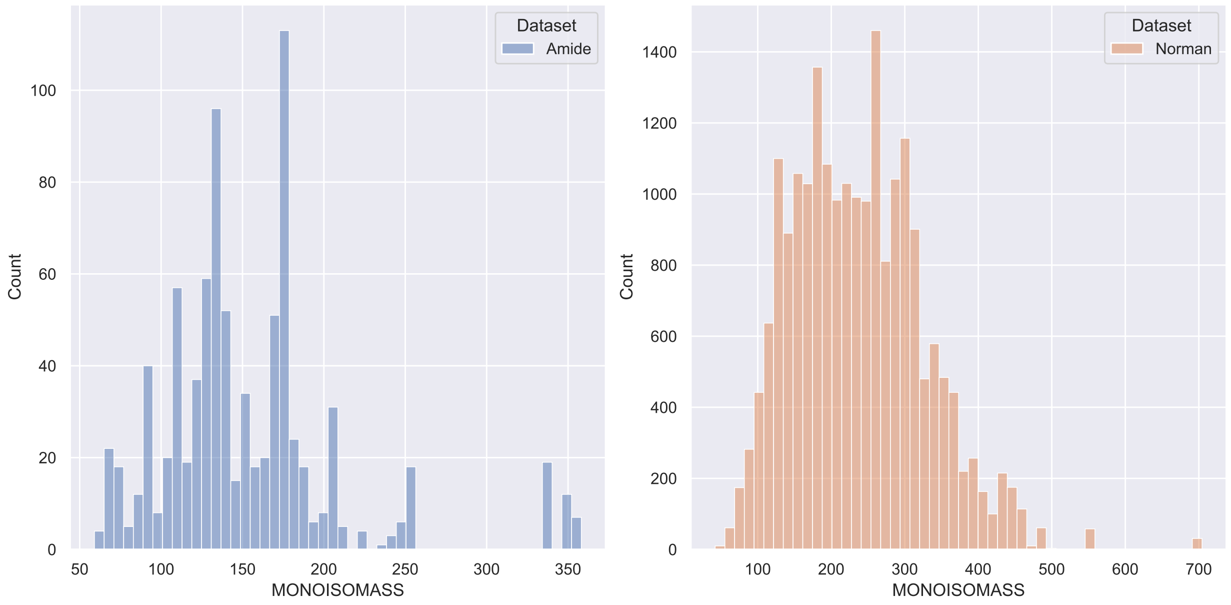

Figure 49: Distribution of MONOISOMASS for Amide dataset (left) and Norman dataset (right).

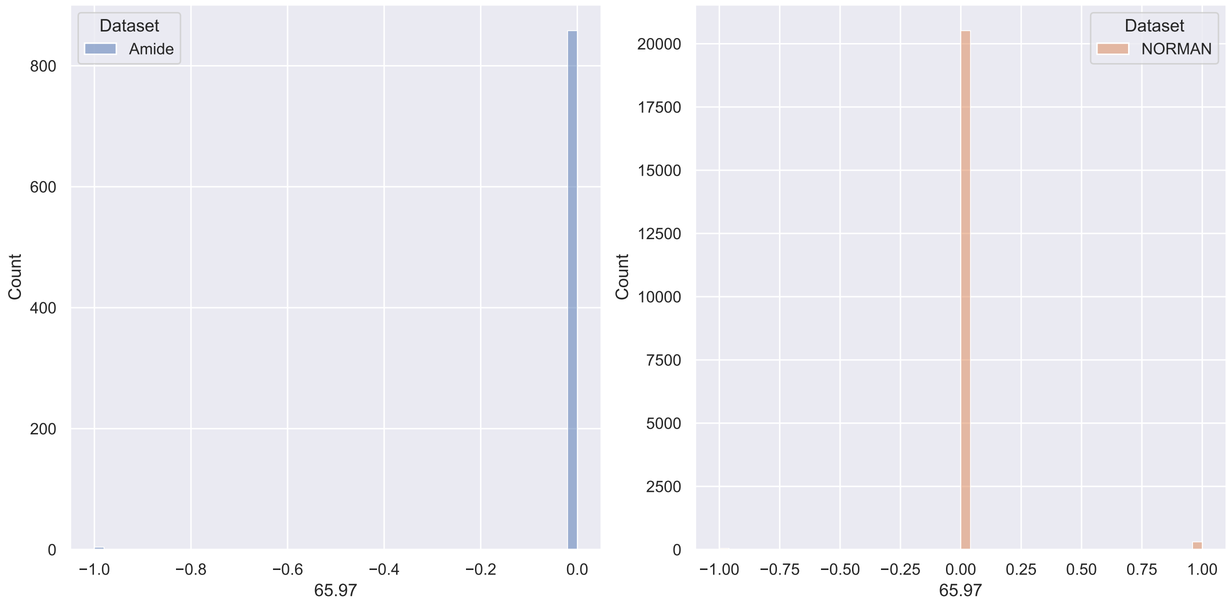

Figure 50: Distribution of neutral loss 65.97 for Amide dataset (left) and Norman dataset (right).

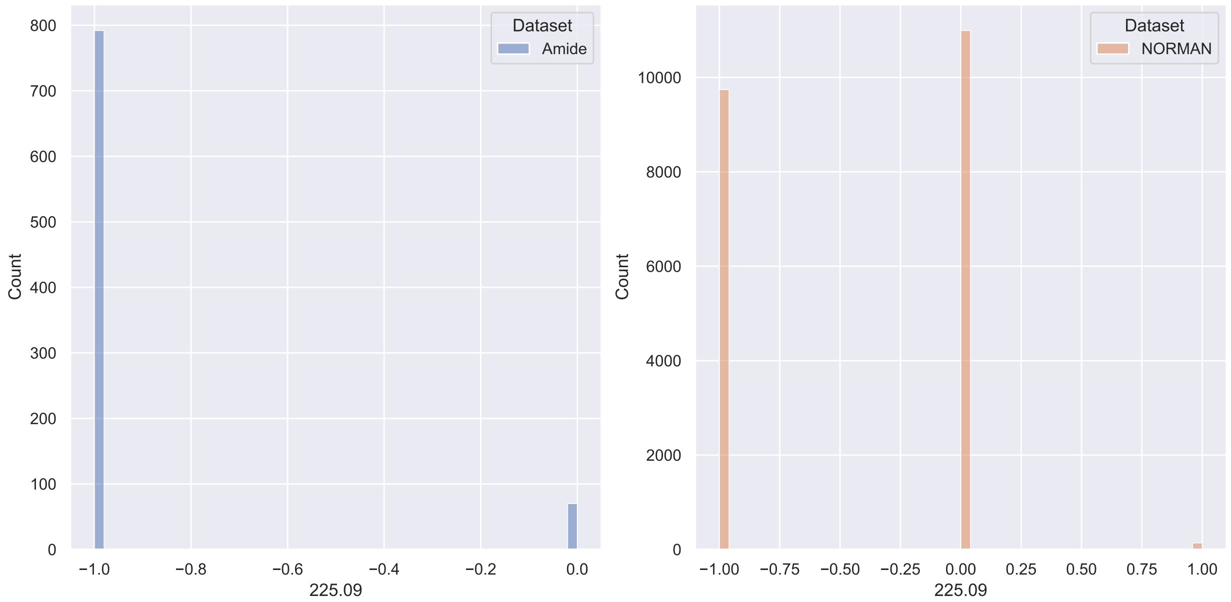

Figure 51: Distribution of neutral loss 225.09 for Amide dataset (left) and Norman dataset (right).

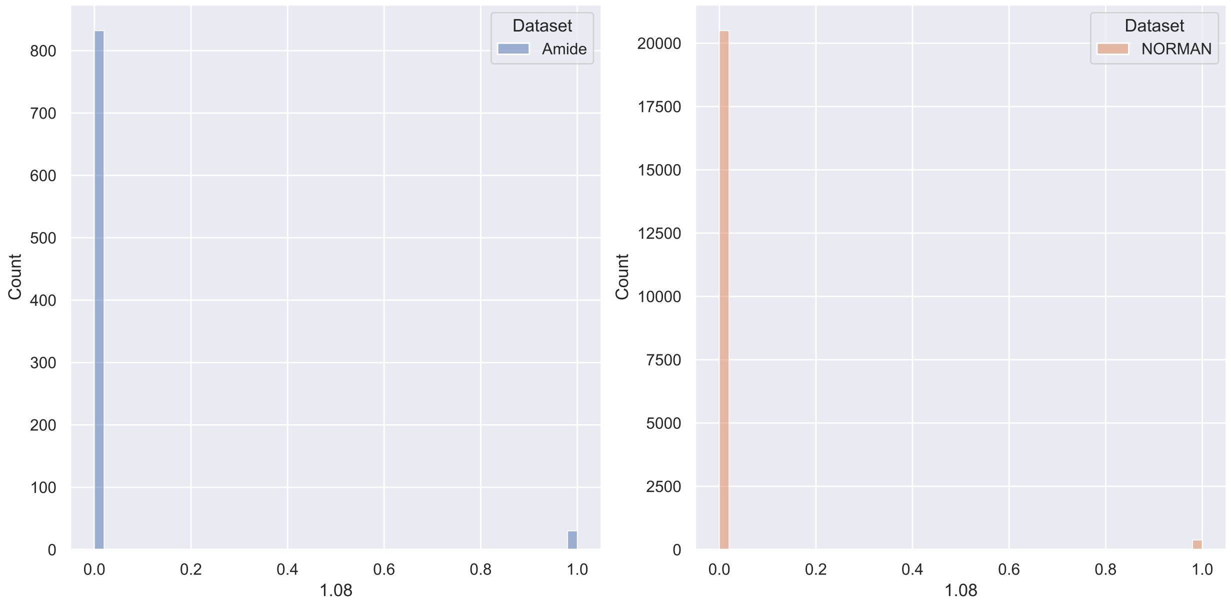

Figure 52: Distribution of neutral loss 1.08 for Amide dataset (left) and Norman dataset (right).

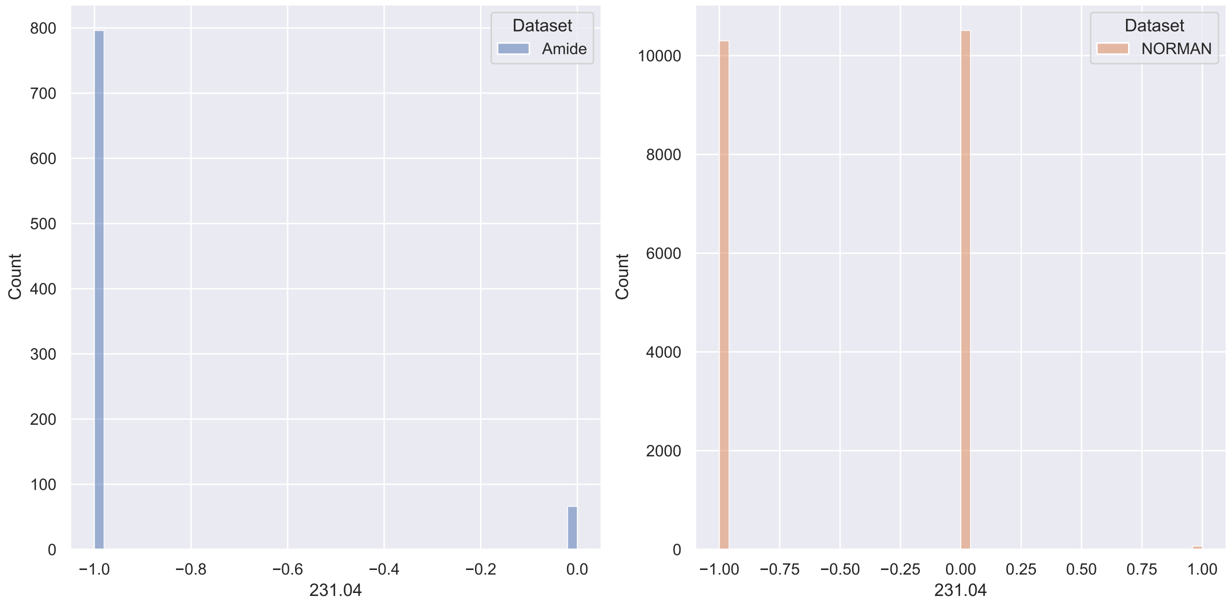

Figure 53: Distribution of neutral loss 231.04 for Amide dataset (left) and Norman dataset (right).

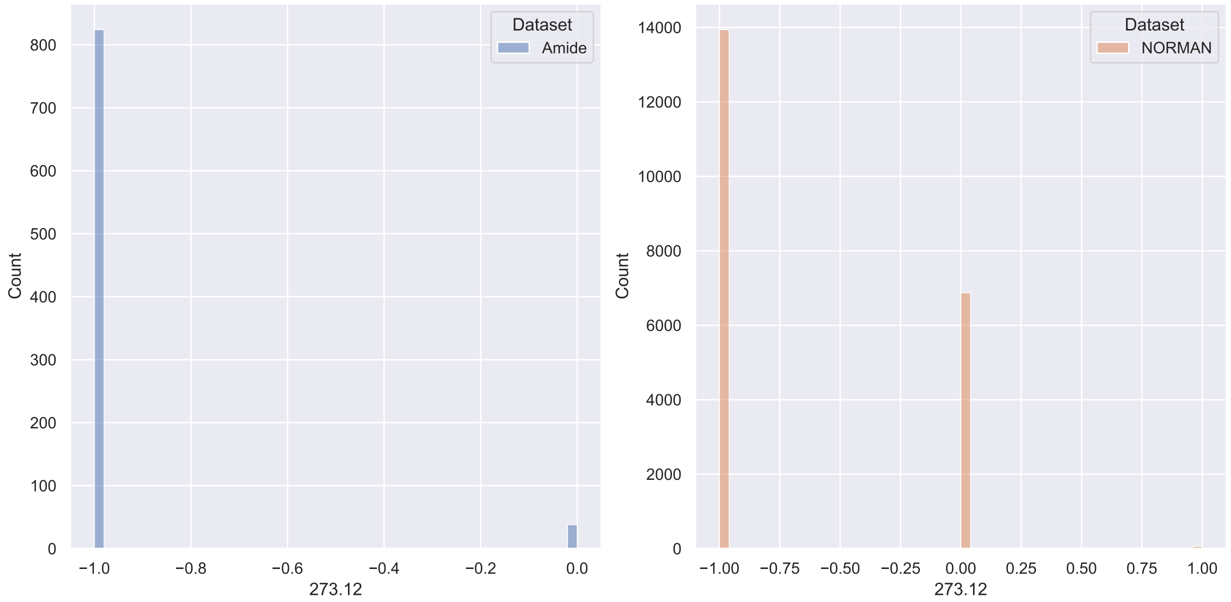

Figure 54: Distribution of neutral loss 273.12 for Amide dataset (left) and Norman dataset (right).

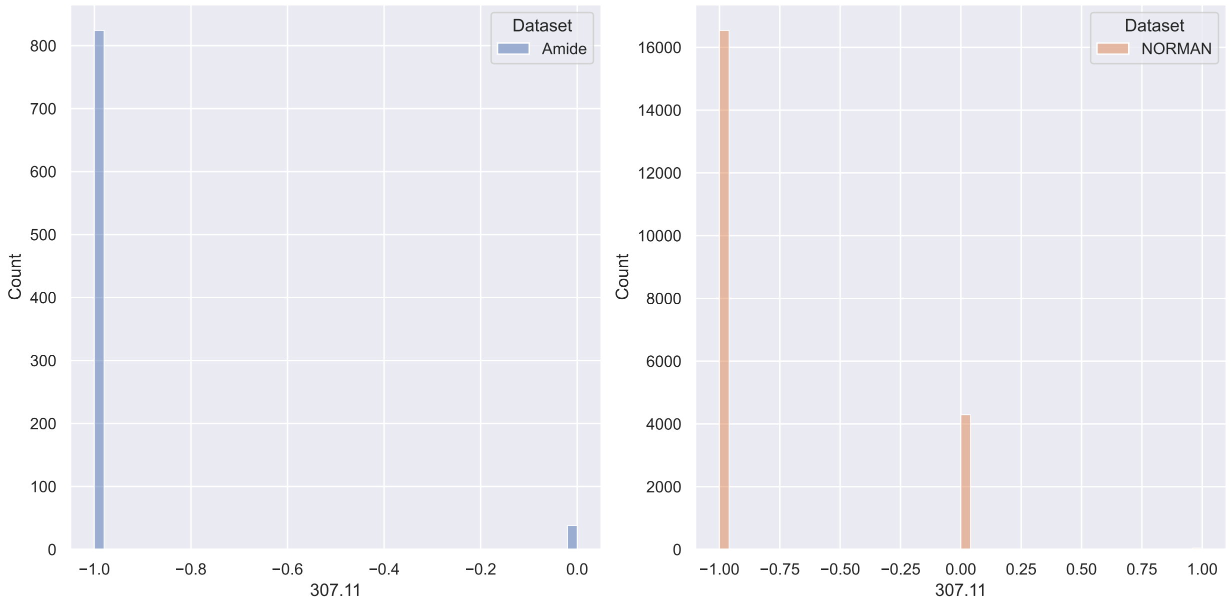

Figure 55: Distribution of neutral loss 307.11 for Amide dataset (left) and Norman dataset (right).

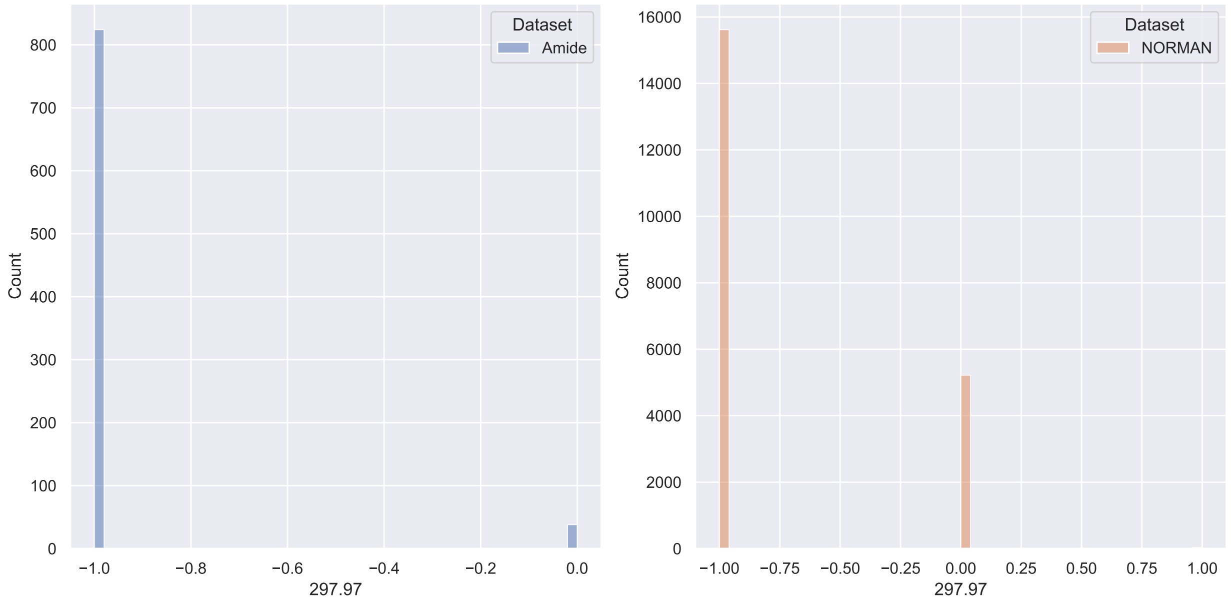

Figure 56: Distribution of neutral loss 297.97 for Amide dataset (left) and Norman dataset (right).

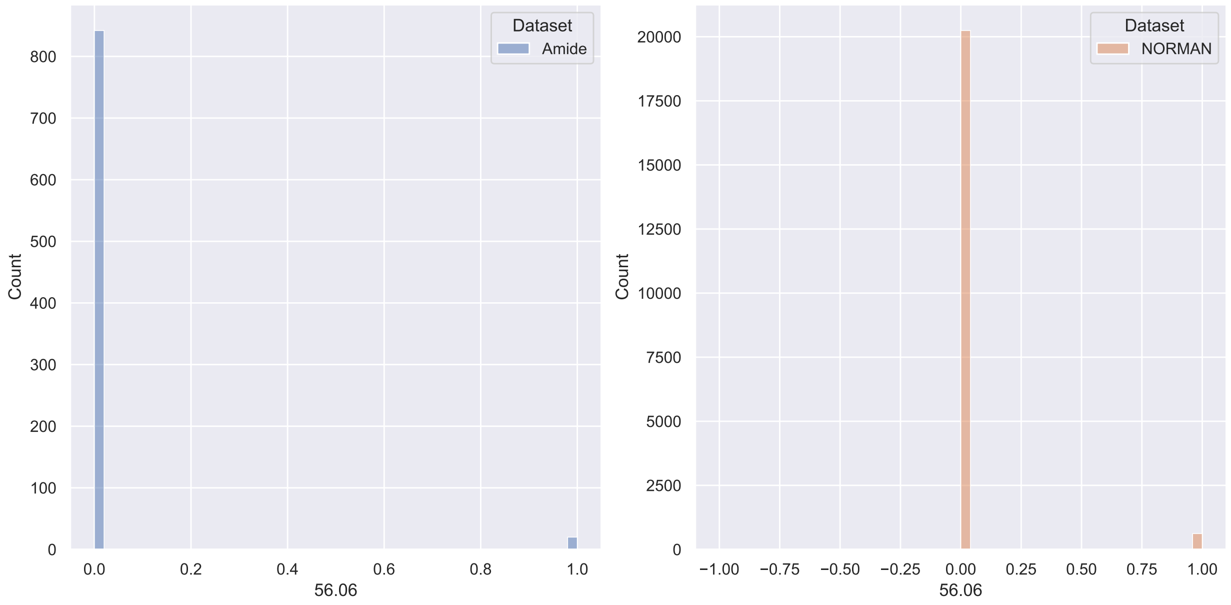

Figure 57: Distribution of neutral loss 56.06 for Amide dataset (left) and Norman dataset (right).

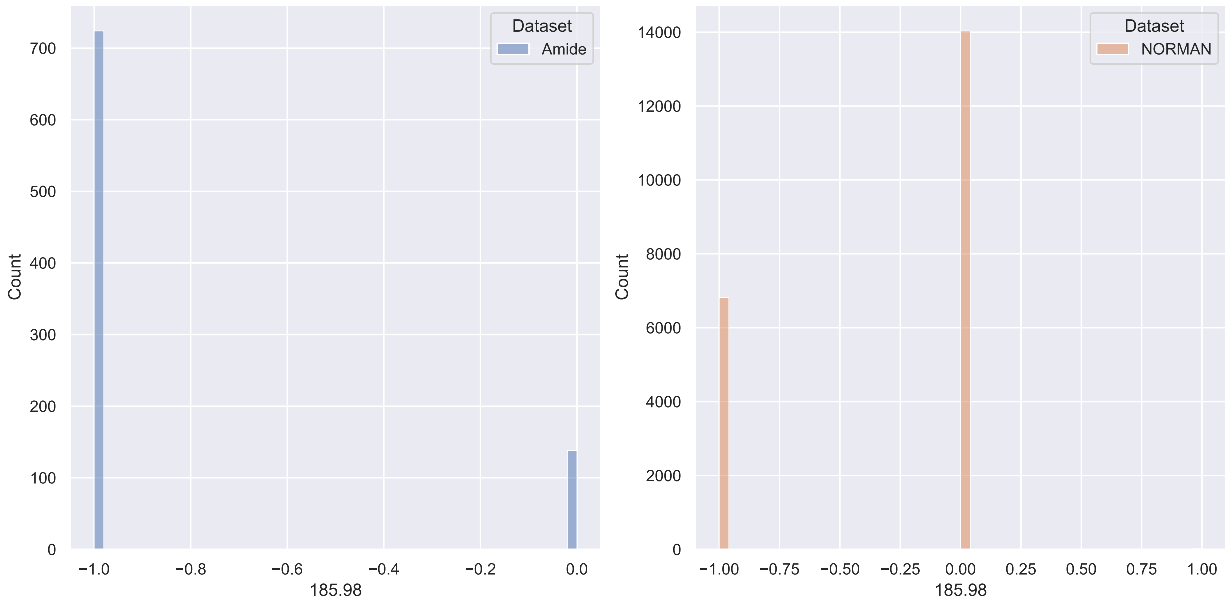

Figure 58: Distribution of neutral loss 185.98 for Amide dataset (left) and Norman dataset (right).

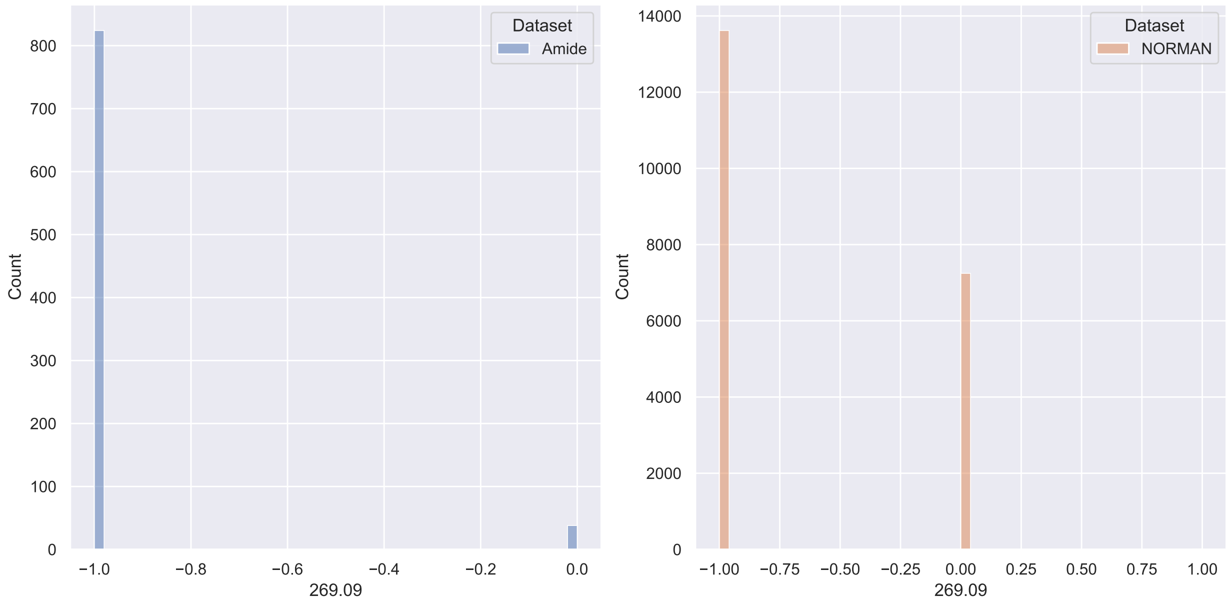

Figure 59: Distribution of neutral loss 269.09 for Amide dataset (left) and Norman dataset (right).

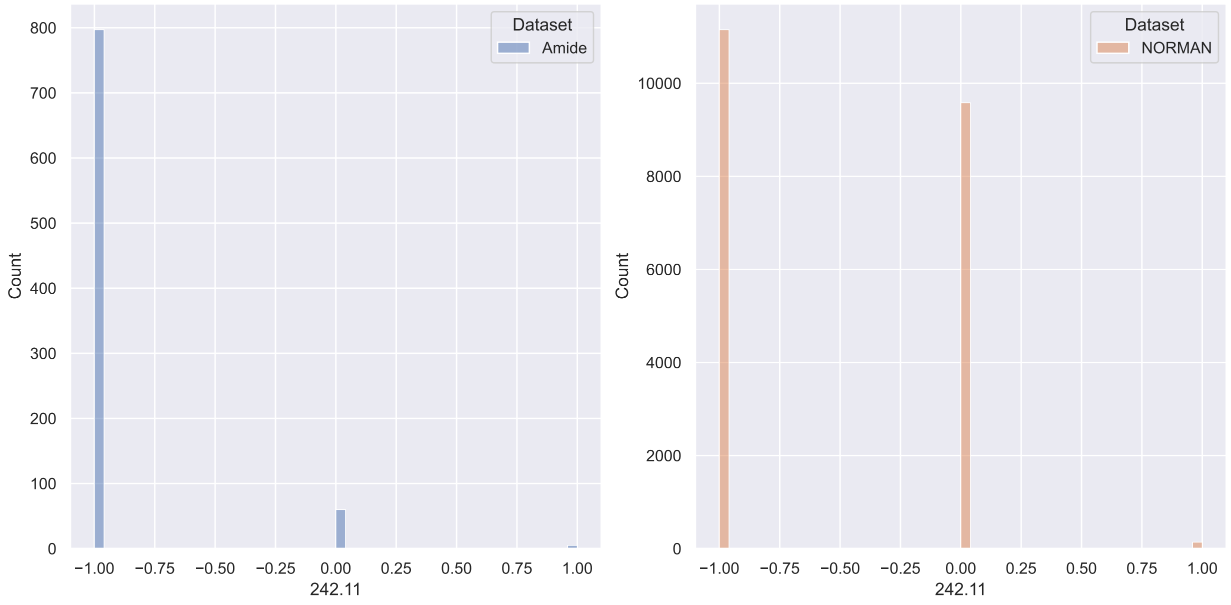

Figure 60: Distribution of neutral loss 242.11 for Amide dataset (left) and Norman dataset (right).

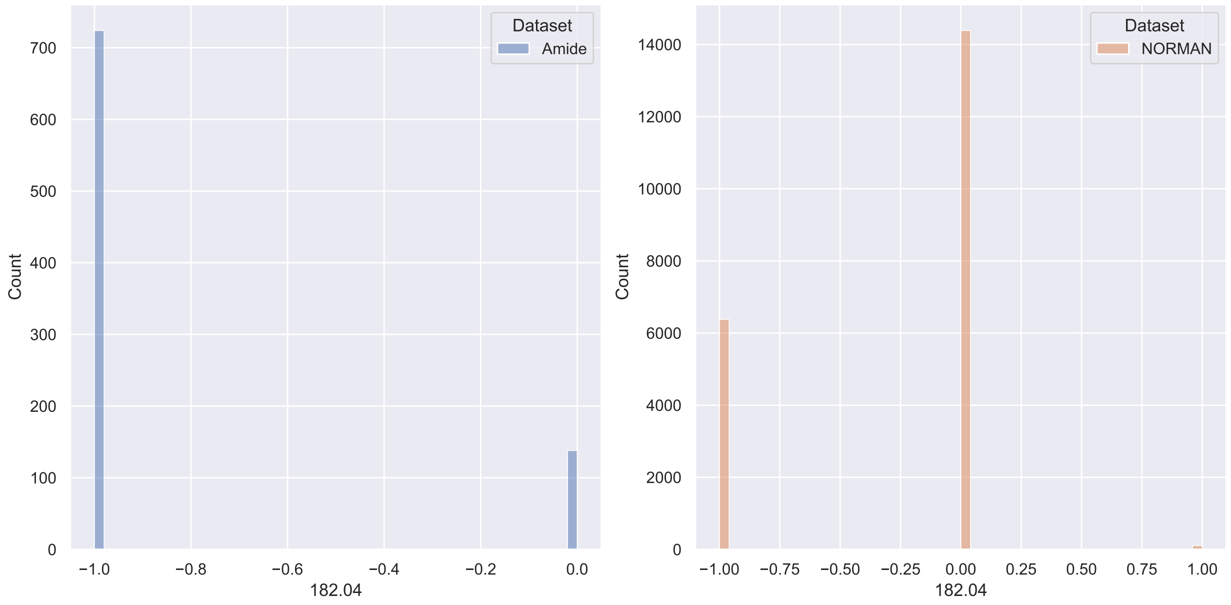

Figure 61: Distribution of neutral loss 182.04 for Amide dataset (left) and Norman dataset (right).

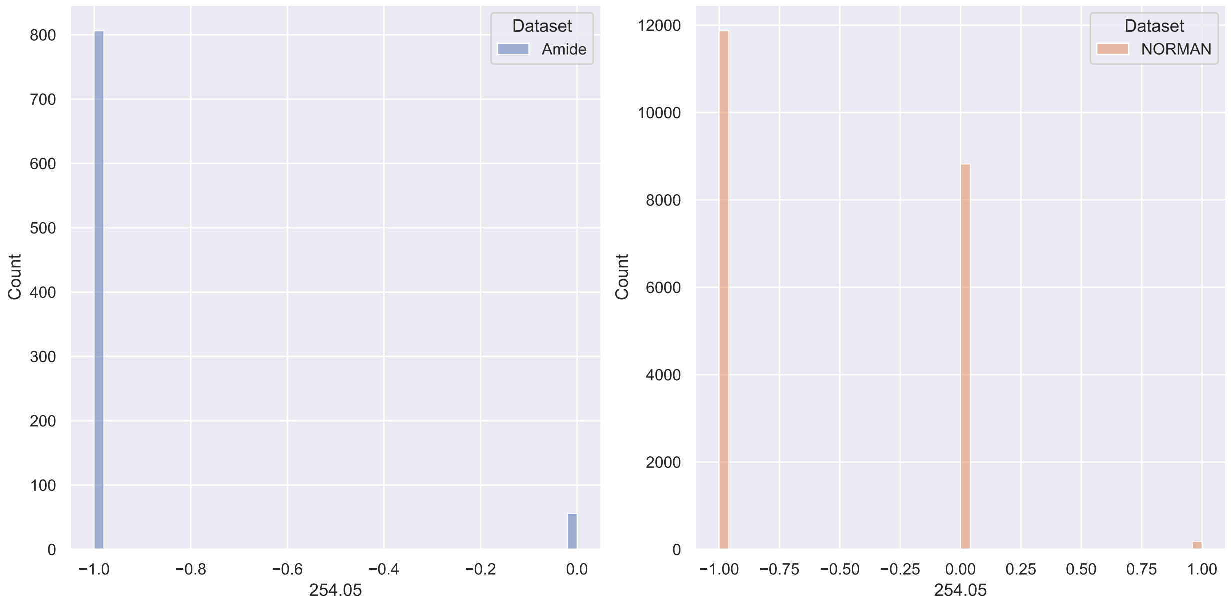

Figure 62: Distribution of neutral loss 254.05 for Amide dataset (left) and Norman dataset (right).

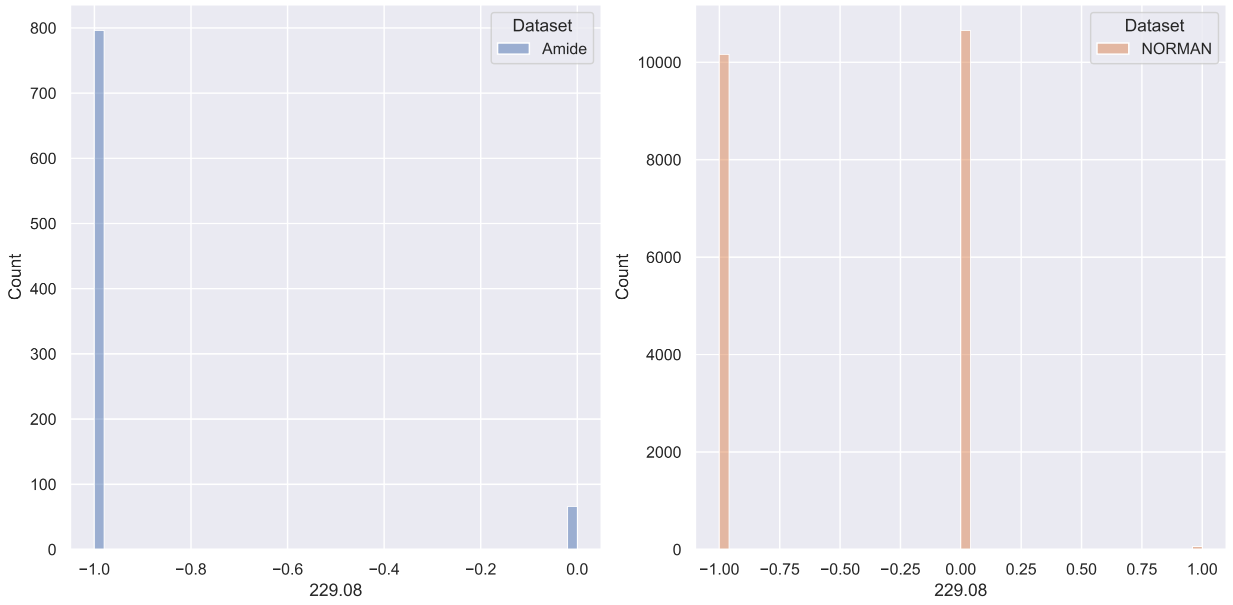

Figure 63: Distribution of neutral loss 229.08 for Amide dataset (left) and Norman dataset (right).

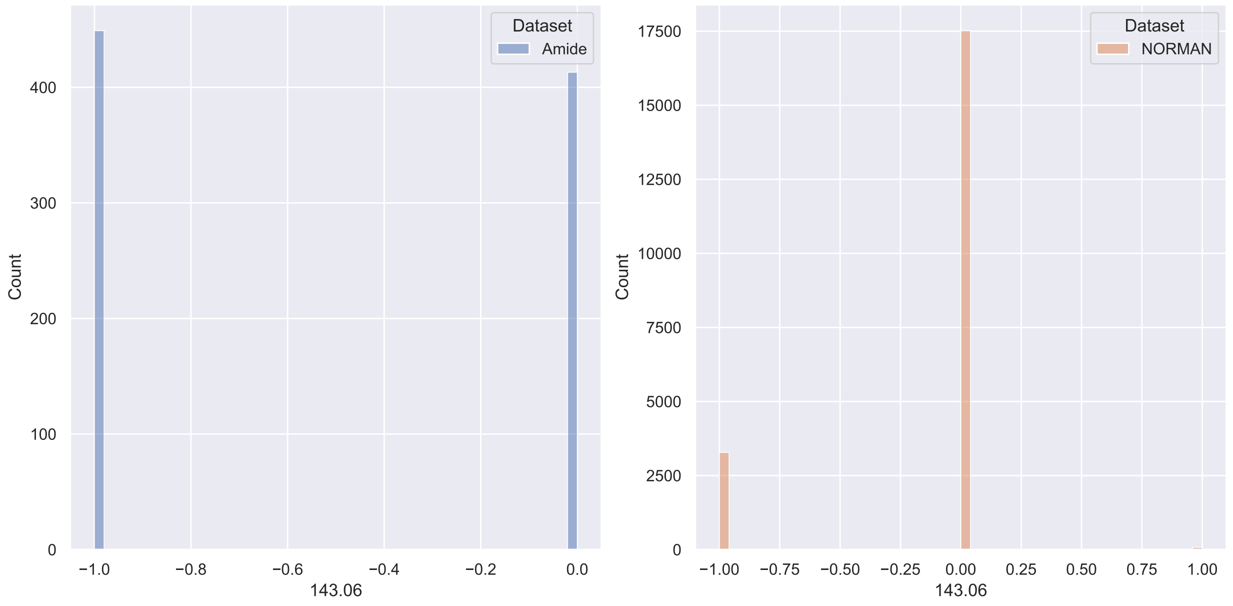

Figure 64: Distribution of neutral loss 143.06 for Amide dataset (left) and Norman dataset (right).

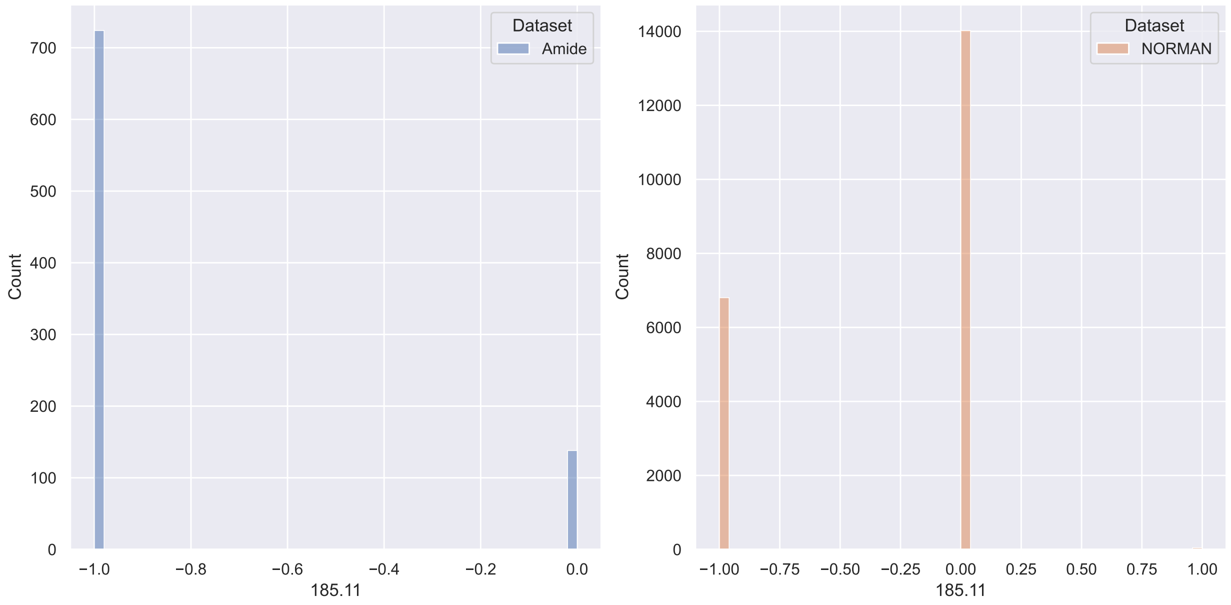

Figure 65: Distribution of neutral loss 185.11 for Amide dataset (left) and Norman dataset (right).

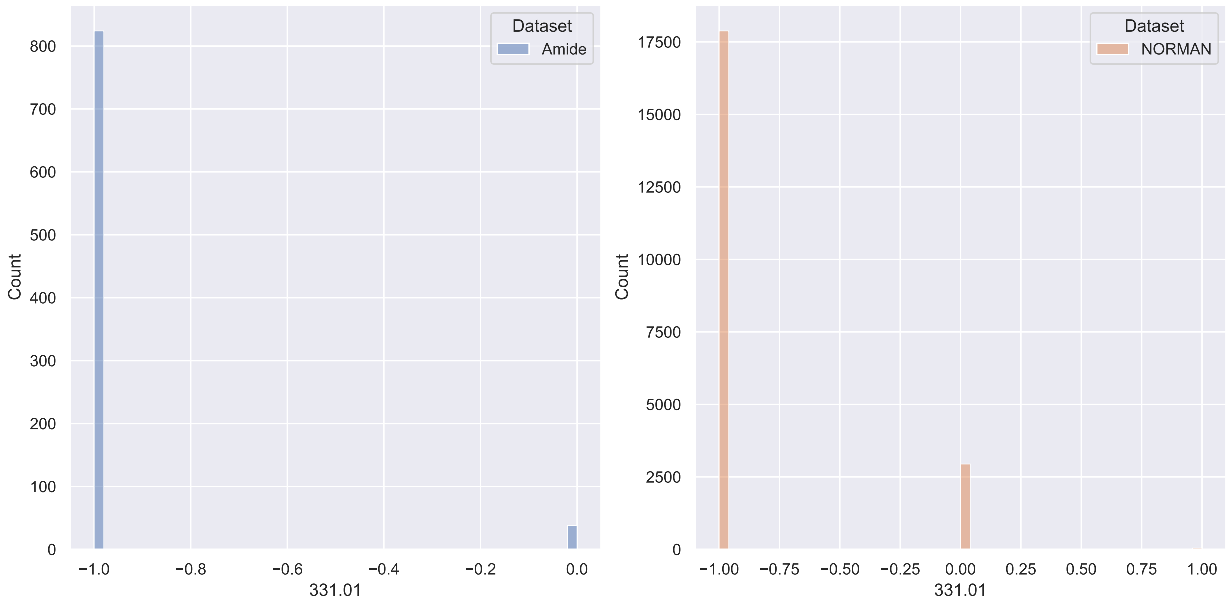

Figure 66: Distribution of neutral loss 331.01 for Amide dataset (left) and Norman dataset (right).

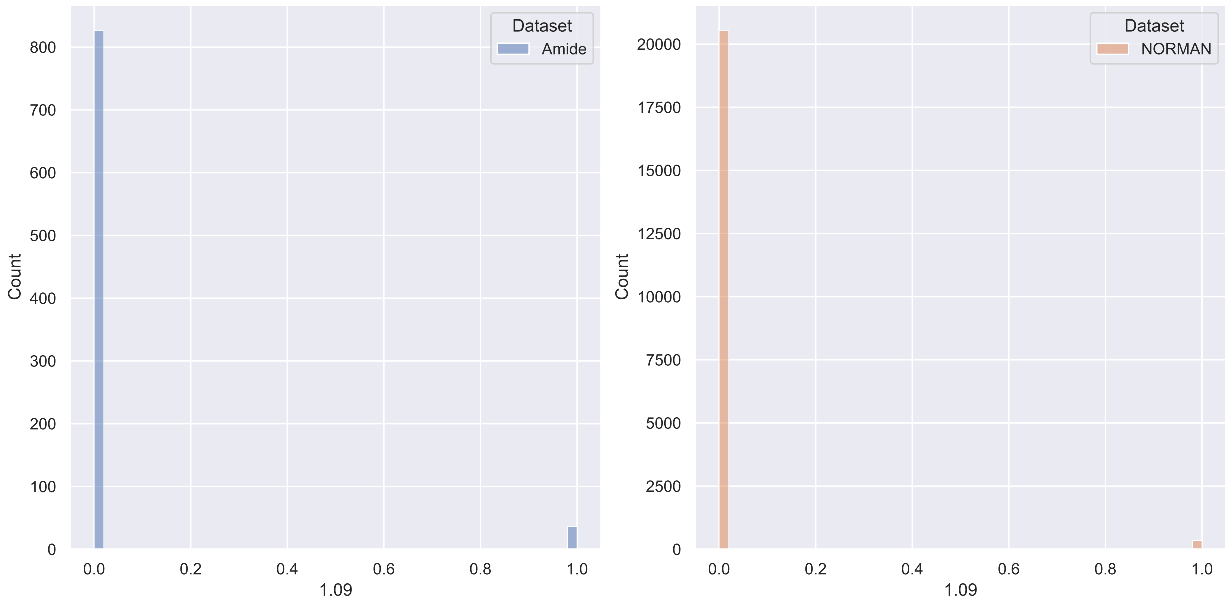

Figure 67: Distribution of neutral loss 1.09 for Amide dataset (left) and Norman dataset (right).

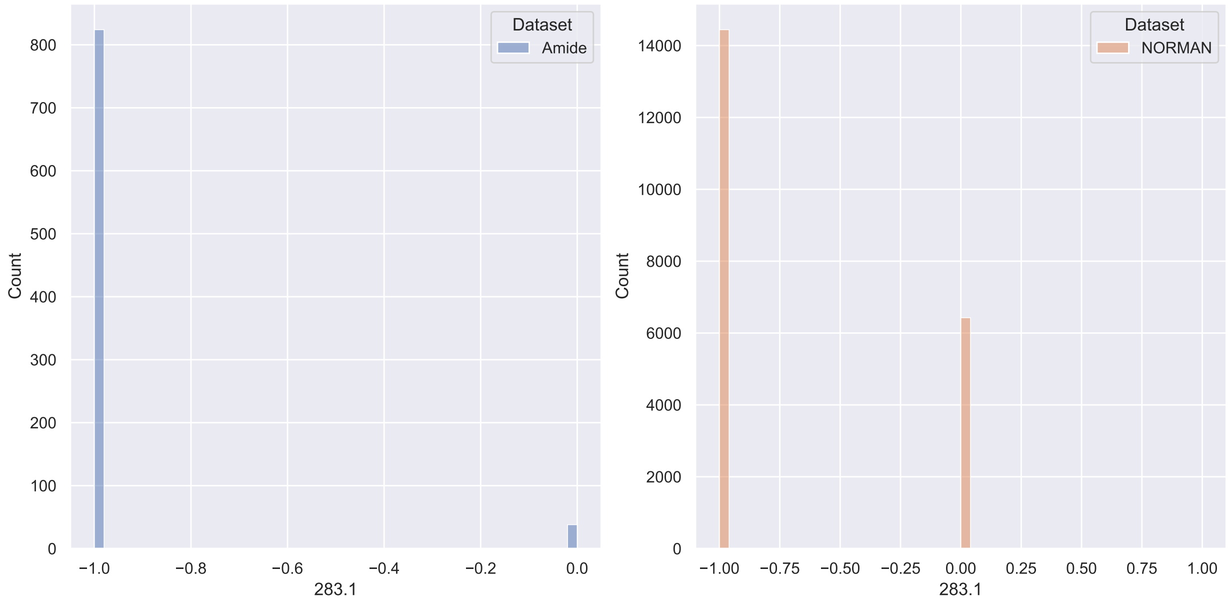

Figure 68: Distribution of neutral loss 283.1 for Amide dataset (left) and Norman dataset (right).

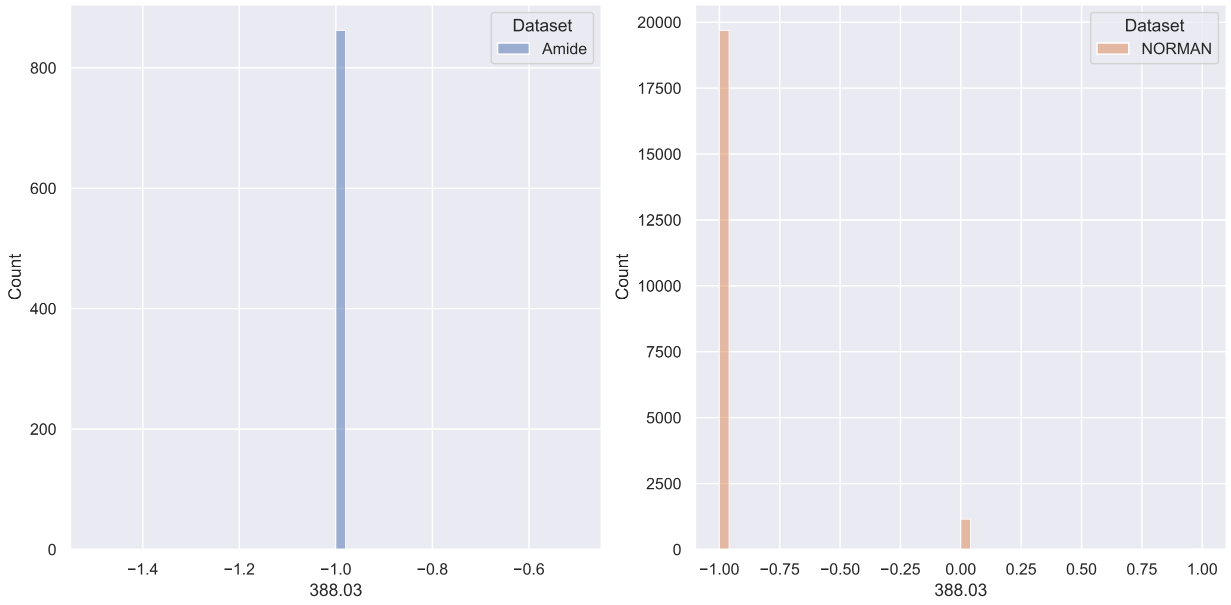

Figure 69: Distribution of neutral loss 388.03 for Amide dataset (left) and Norman dataset (right).

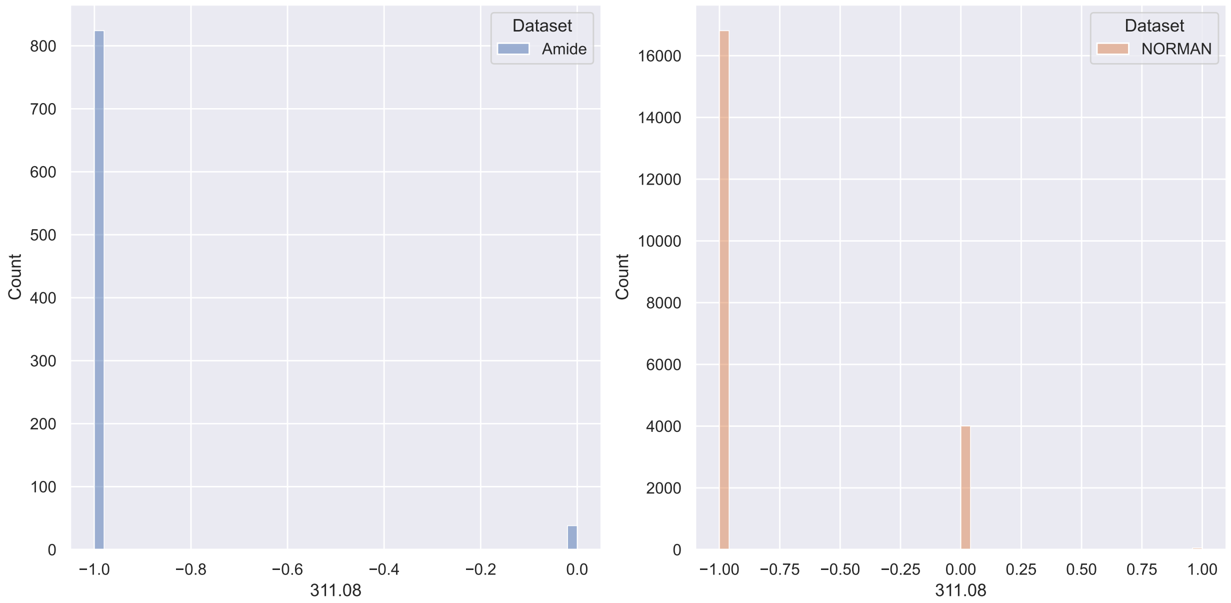

Figure 70: Distribution of neutral loss 311.08 for Amide dataset (left) and Norman dataset (right).

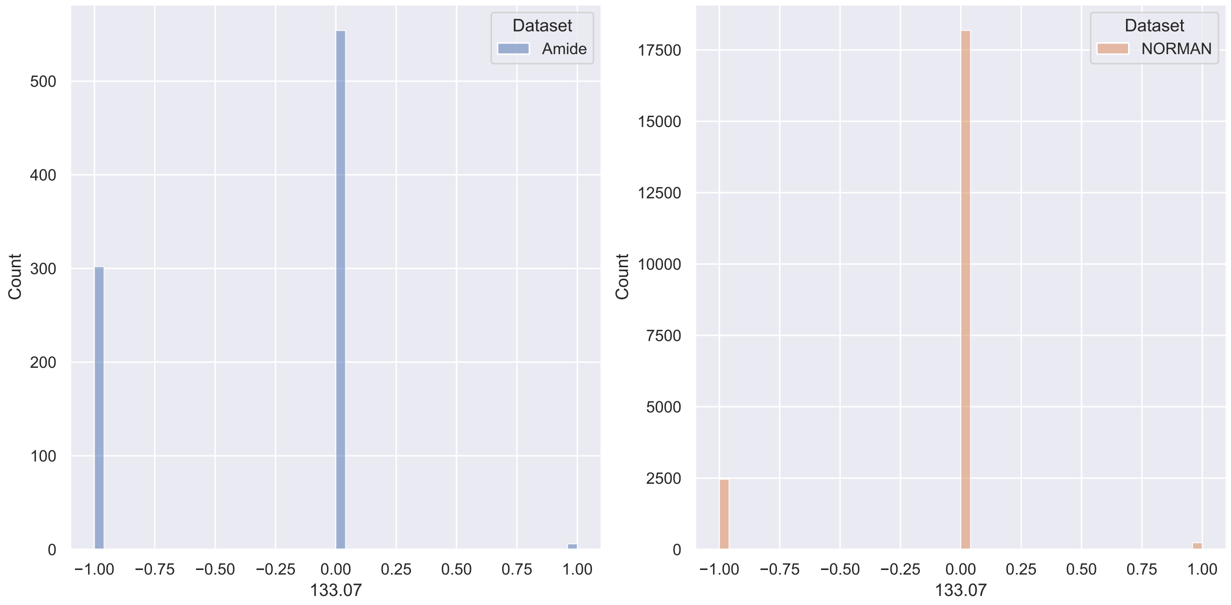

Figure 71: Distribution of neutral loss 133.07 for Amide dataset (left) and Norman dataset (right).

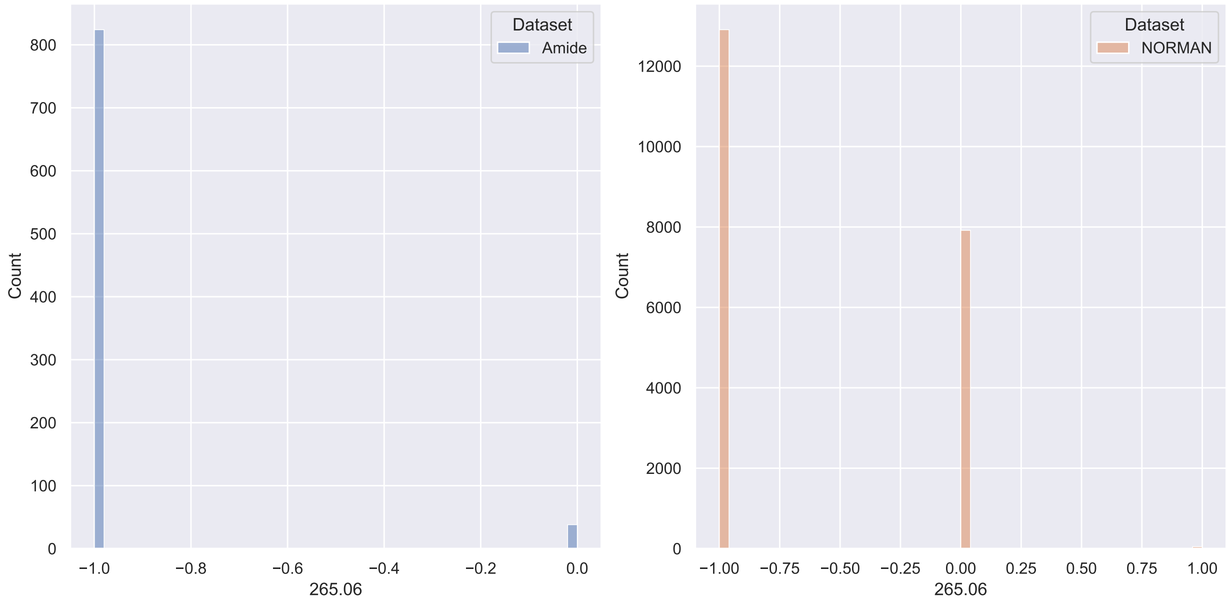

Figure 72: Distribution of neutral loss 265.06 for Amide dataset (left) and Norman dataset (right).

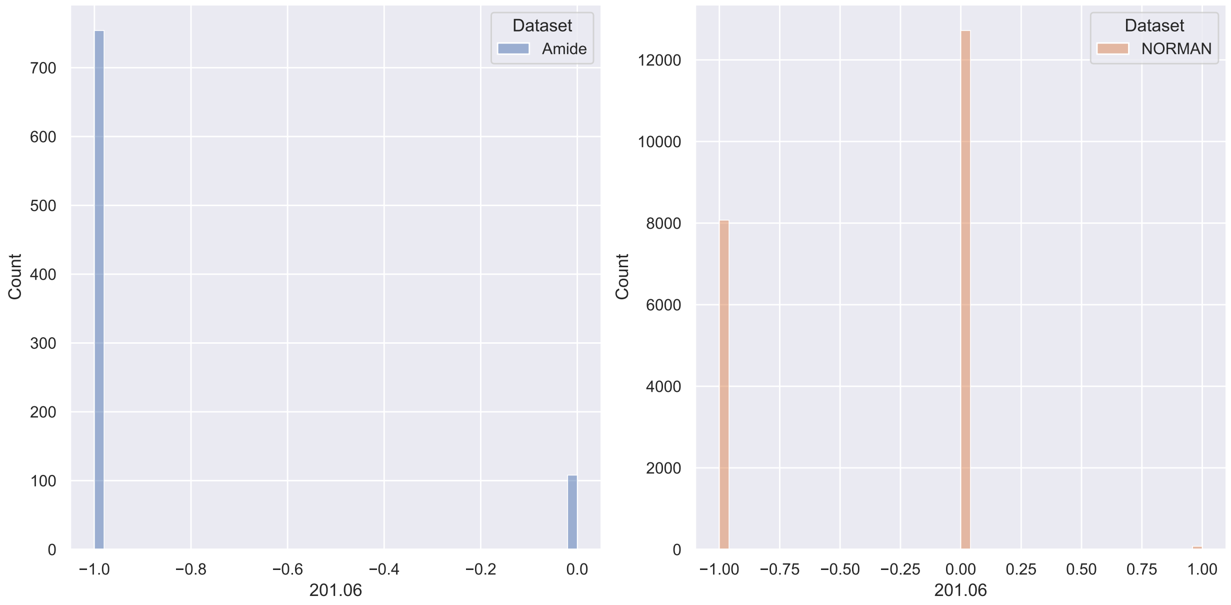

Figure 73: Distribution of neutral loss 201.06 for Amide dataset (left) and Norman dataset (right).

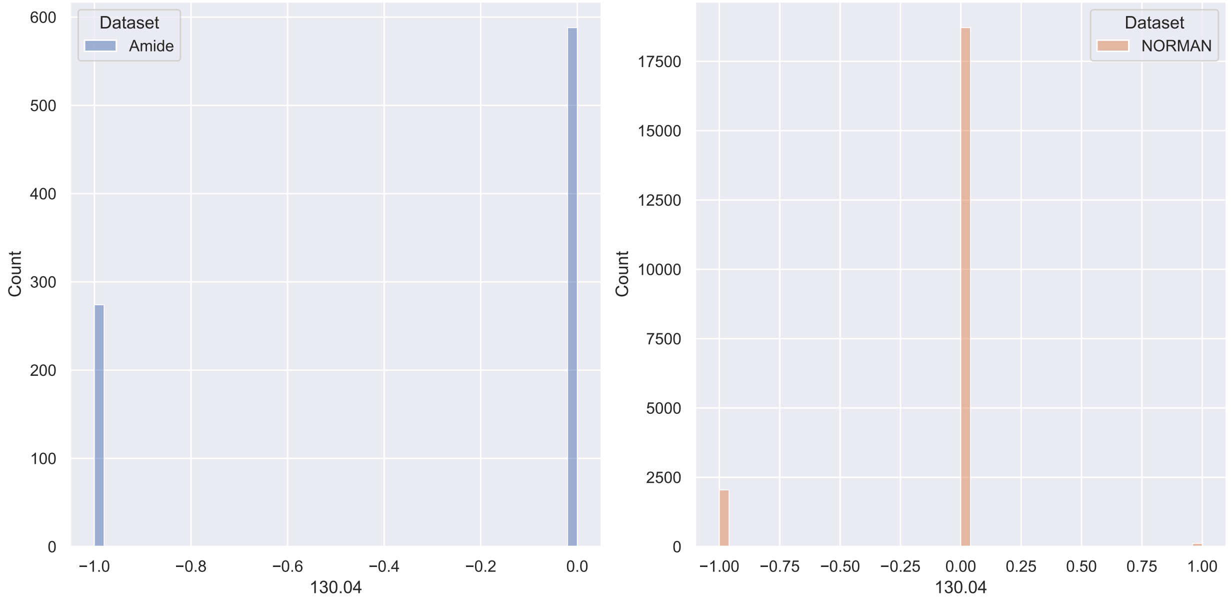

Figure 74: Distribution of neutral loss 130.04 for Amide dataset (left) and Norman dataset (right).

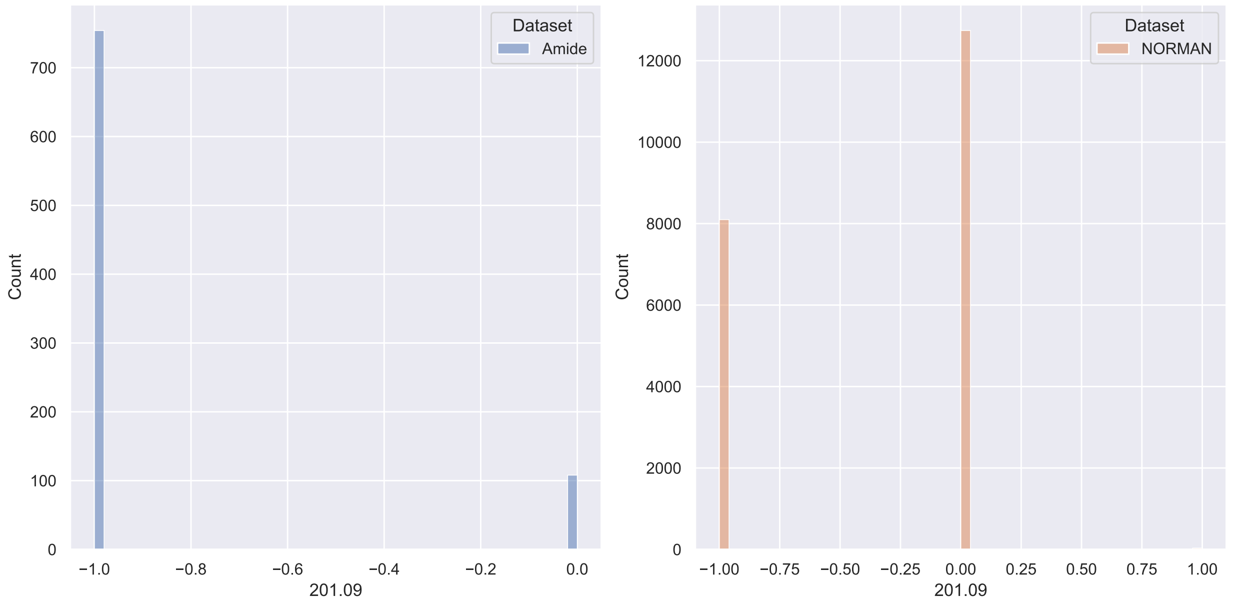

Figure 75: Distribution of neutral loss 201.09 for Amide dataset (left) and Norman dataset (right).

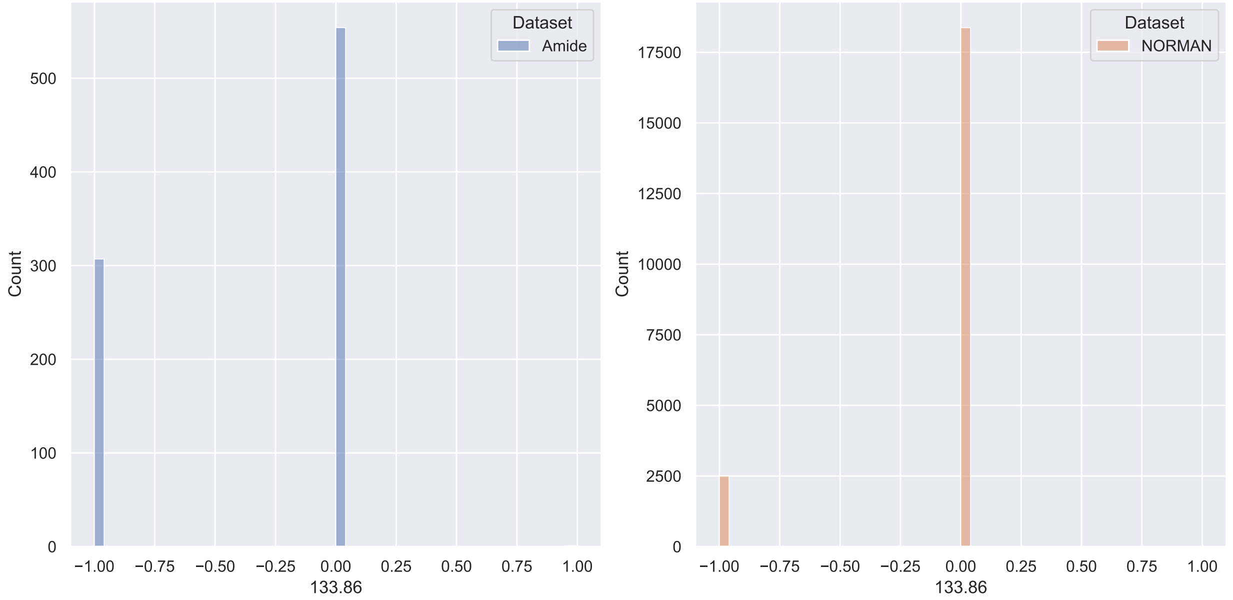

Figure 76: Distribution of neutral loss 133.86 for Amide dataset (left) and Norman dataset (right).

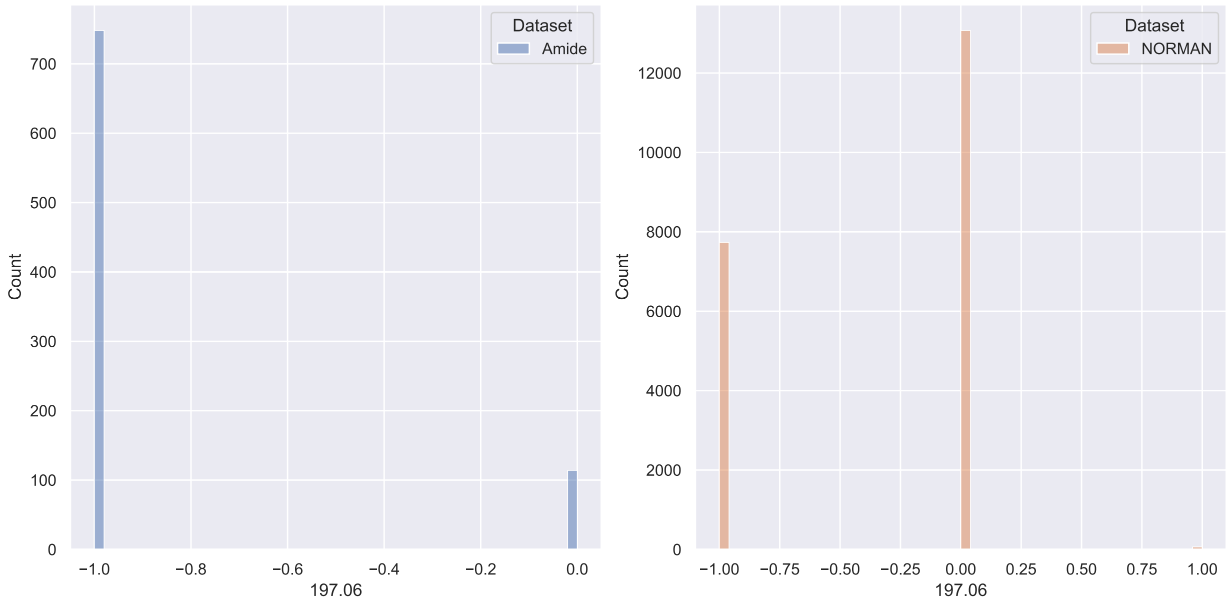

Figure 77: Distribution of neutral loss 197.06 for Amide dataset (left) and Norman dataset (right).

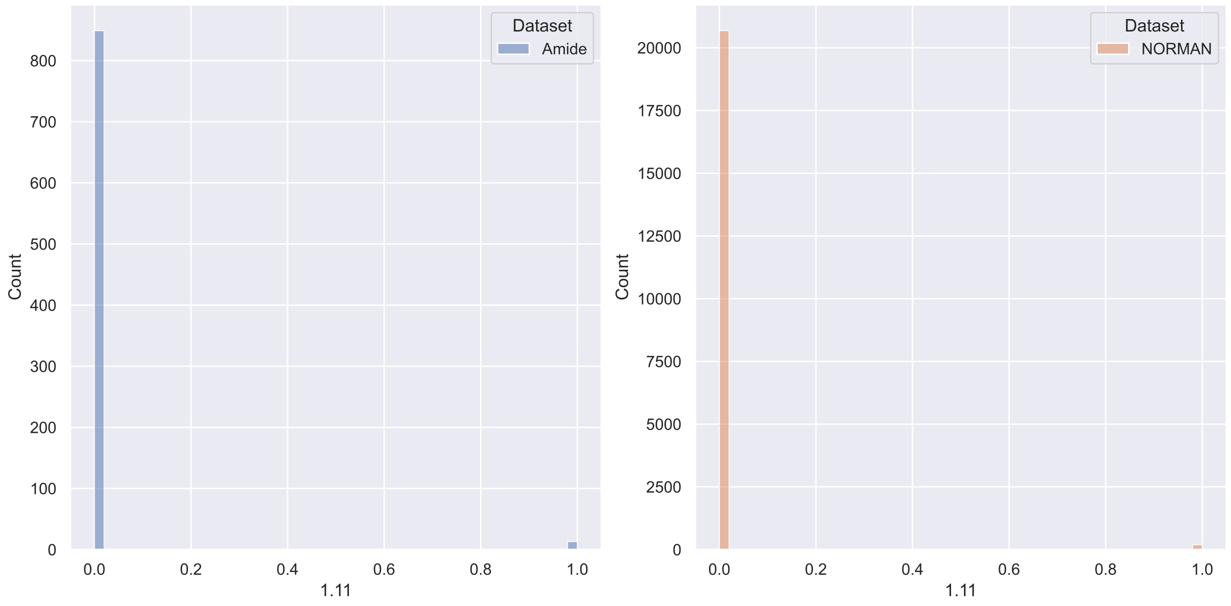

Figure 78: Distribution of neutral loss 1.11 for Amide dataset (left) and Norman dataset (right).

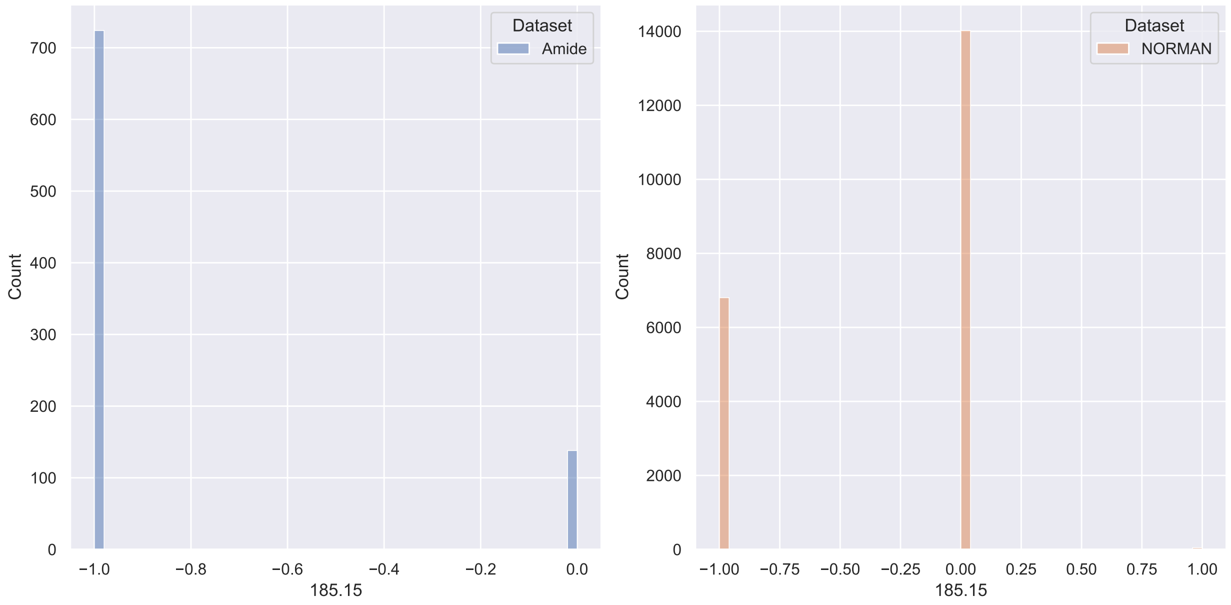

Figure 79: Distribution of neutral loss 185.15 for Amide dataset (left) and Norman dataset (right).

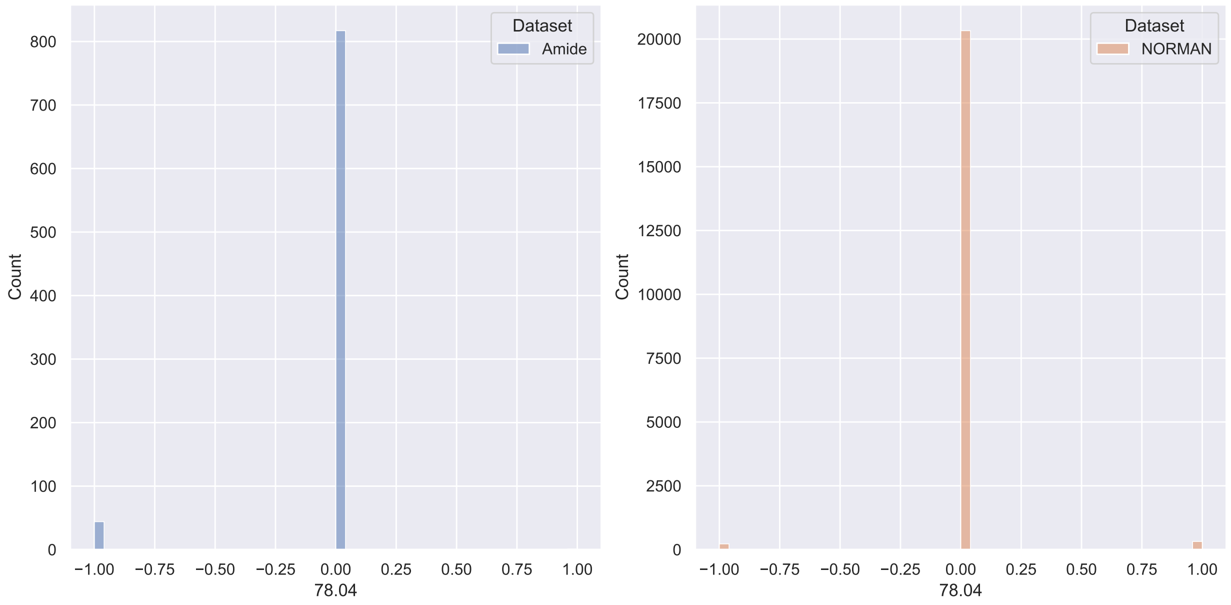

Figure 80: Distribution of neutral loss 78.04 for Amide dataset (left) and Norman dataset (right).

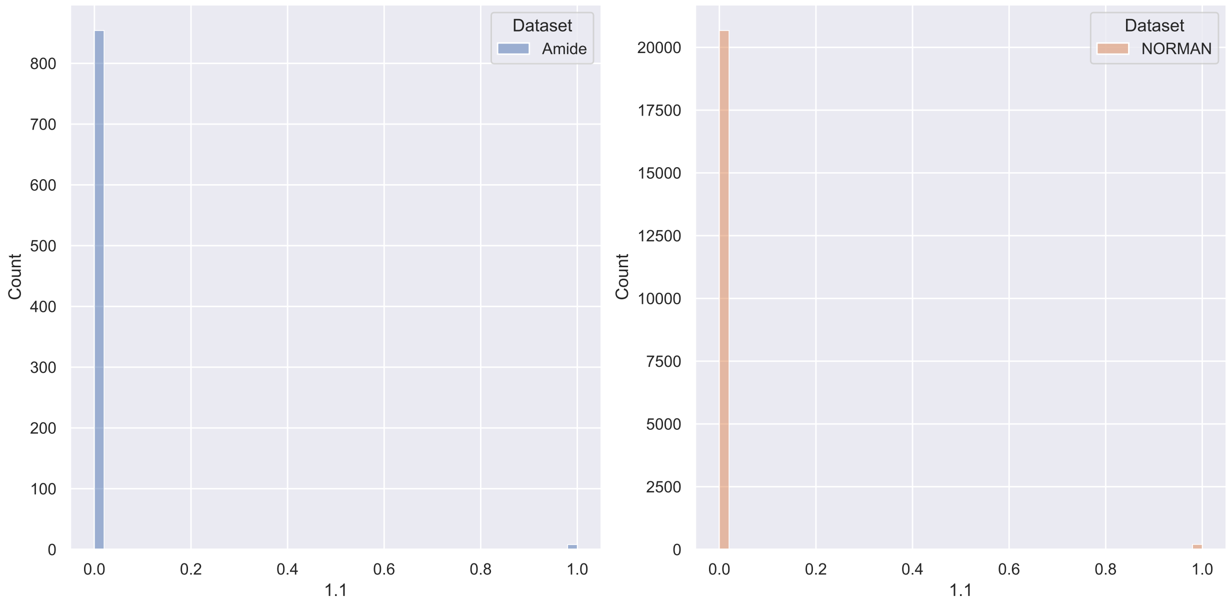

Figure 81: Distribution of neutral loss 1.1 for Amide dataset (left) and Norman dataset (right).

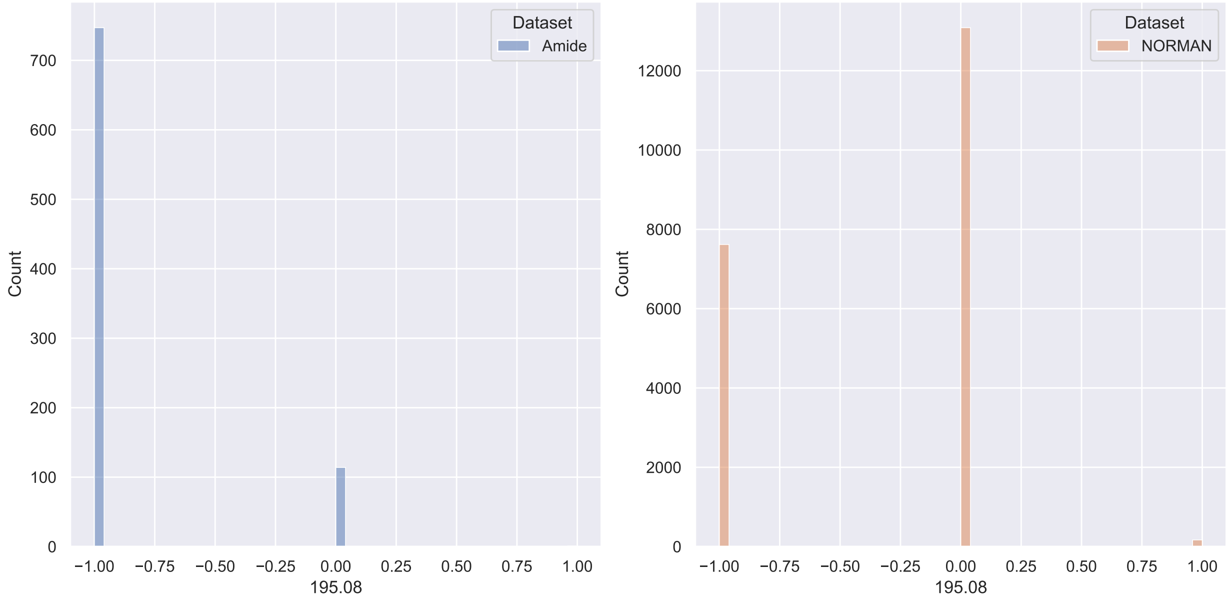

Figure 82: Distribution of neutral loss 195.08 for Amide dataset (left) and Norman dataset (right).

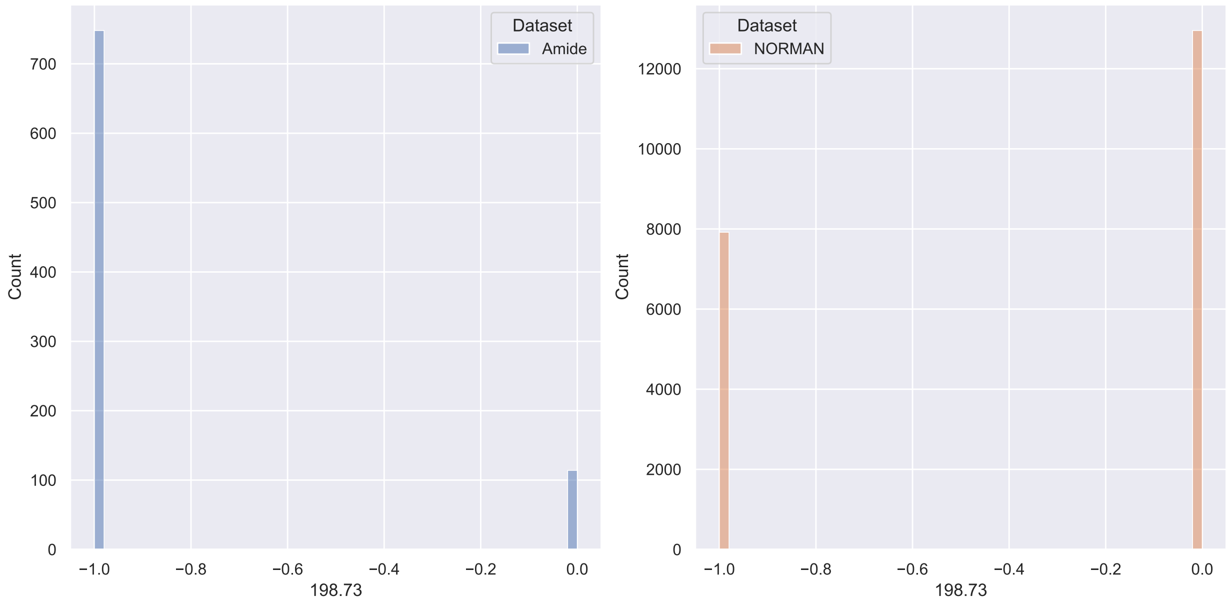

Figure 83: Distribution of neutral loss 198.73 for Amide dataset (left) and Norman dataset (right).

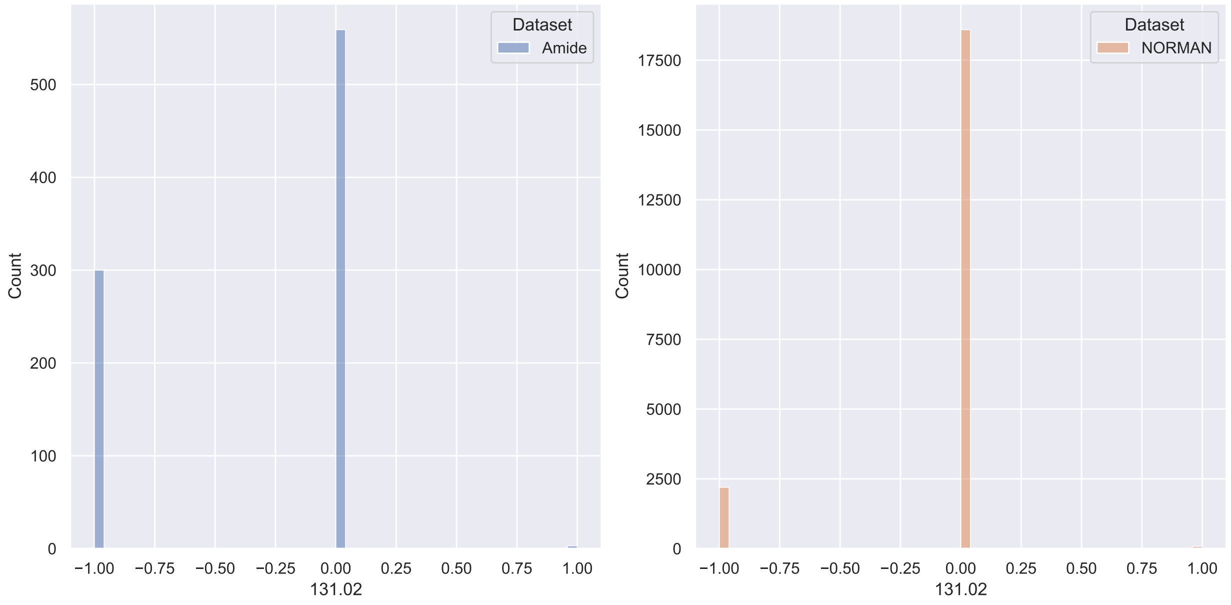

Figure 84: Distribution of neutral loss 131.02 for Amide dataset (left) and Norman dataset (right).

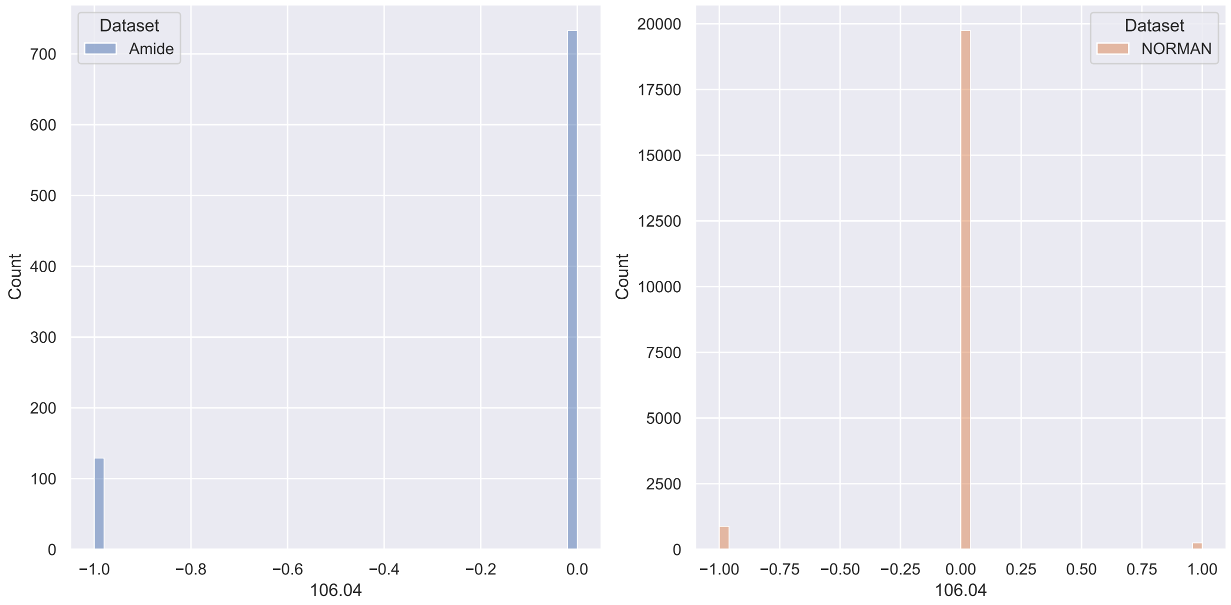

Figure 85: Distribution of neutral loss 106.04 for Amide dataset (left) and Norman dataset (right).

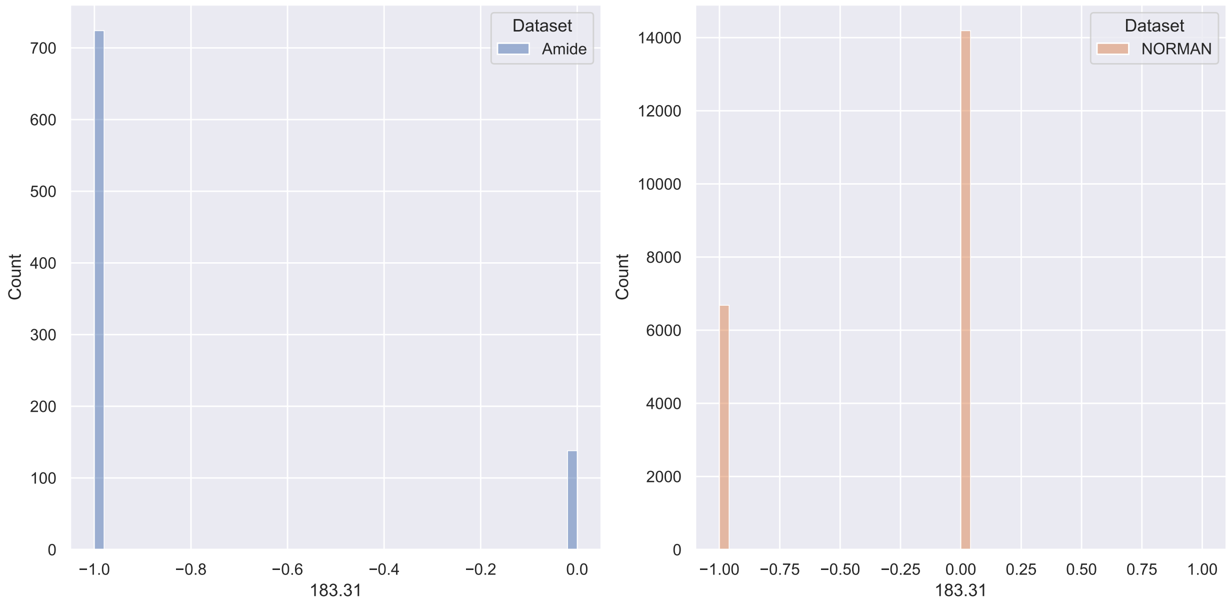

Figure 86: Distribution of neutral loss 183.31 for Amide dataset (left) and Norman dataset (right).

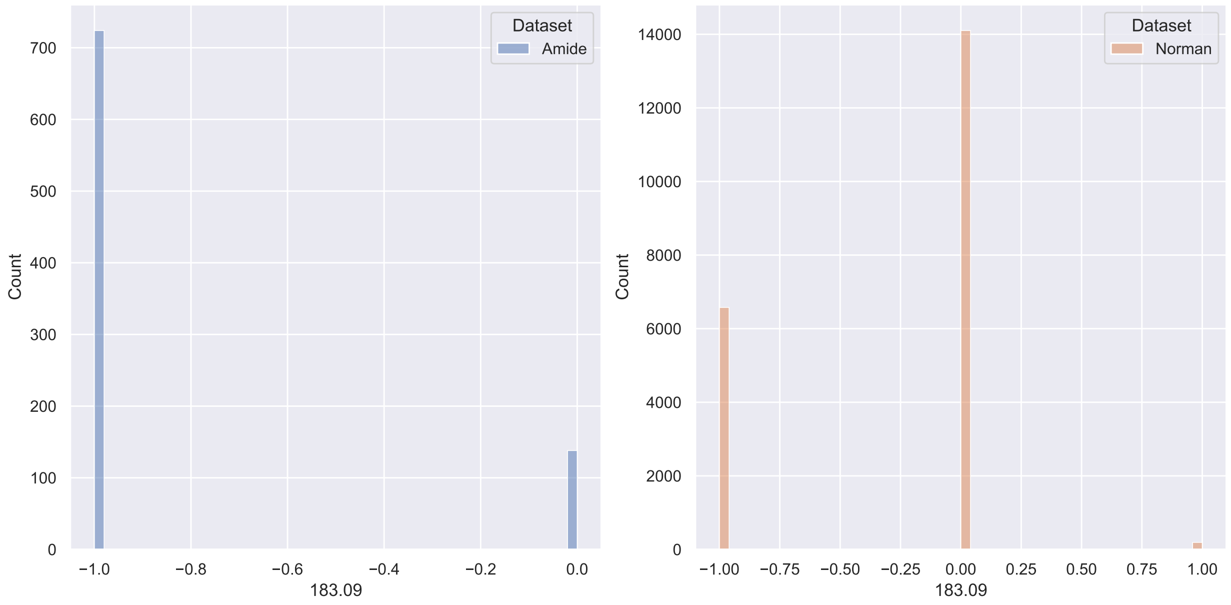

Figure 87: Distribution of neutral loss 183.09 for Amide dataset (left) and Norman dataset (right).

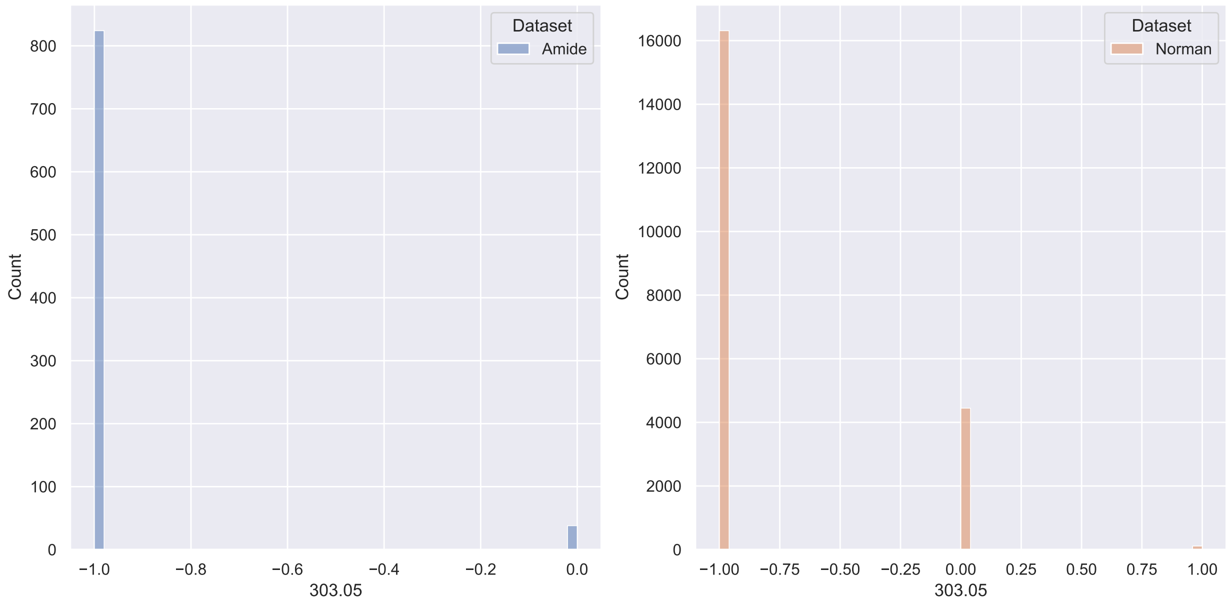

Figure 88: Distribution of neutral loss 303.05 for Amide dataset (left) and Norman dataset (right).

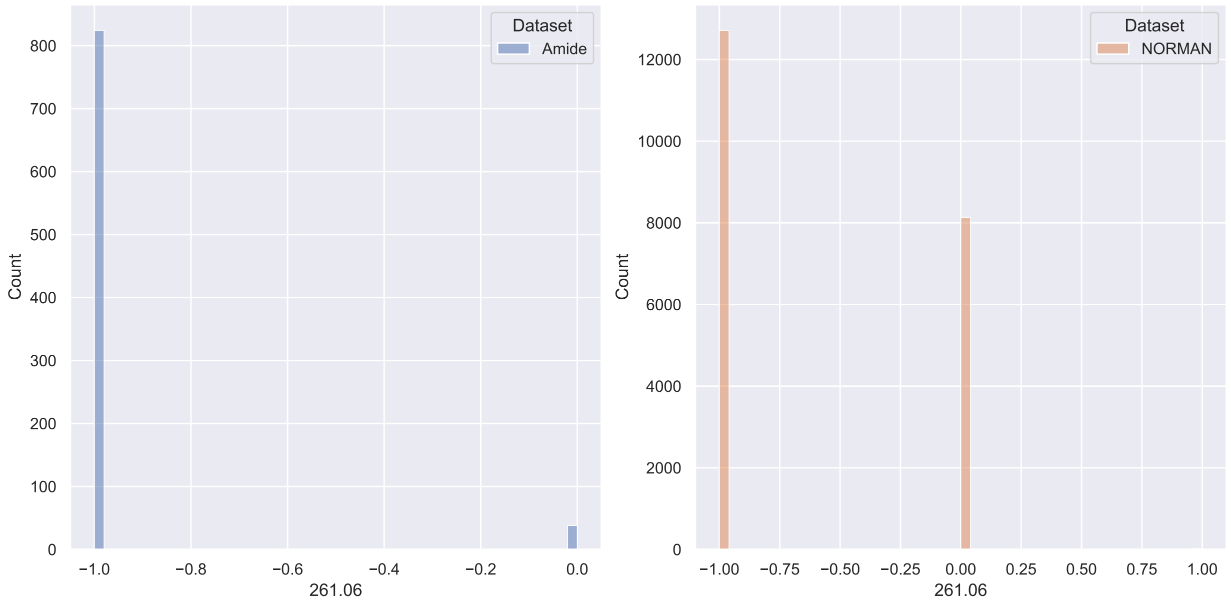

Figure 89: Distribution of neutral loss 261.06 for Amide dataset (left) and Norman dataset (right).

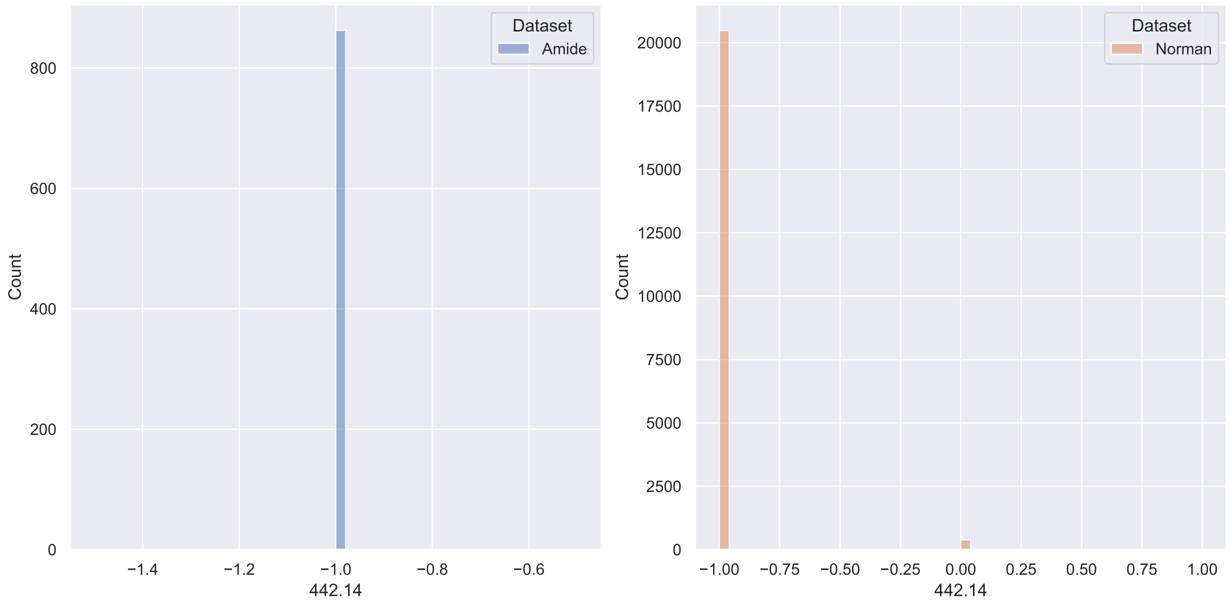

Figure 90: Distribution of neutral loss 442.14 for Amide dataset (left) and Norman dataset (right).

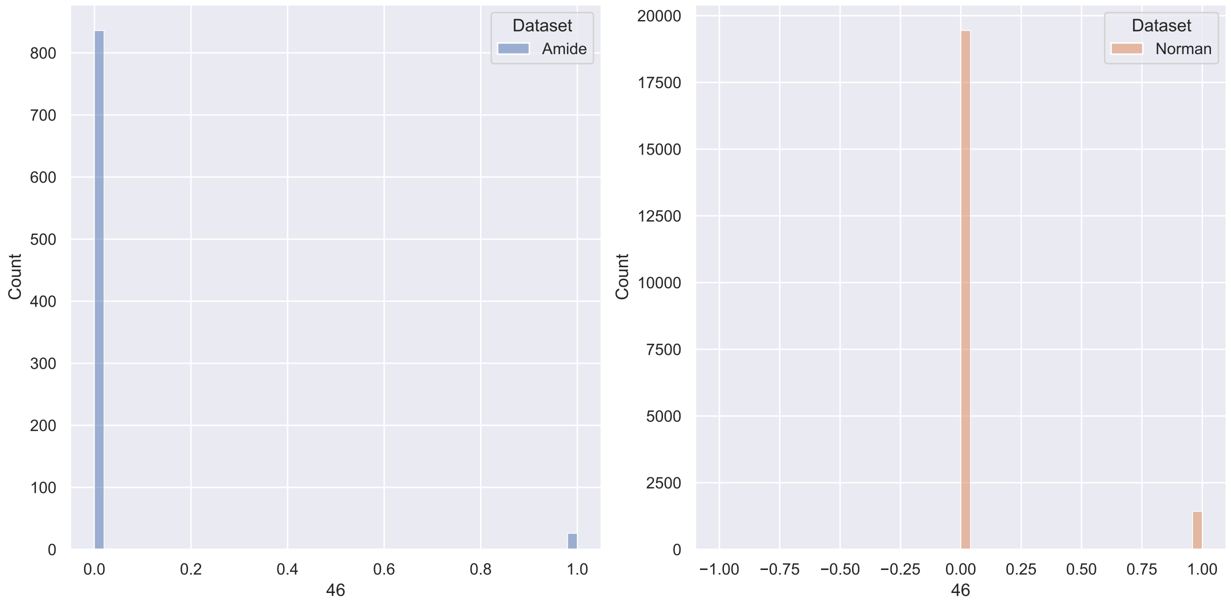

Figure 91: Distribution of neutral loss 46 for Amide dataset (left) and Norman dataset (right).

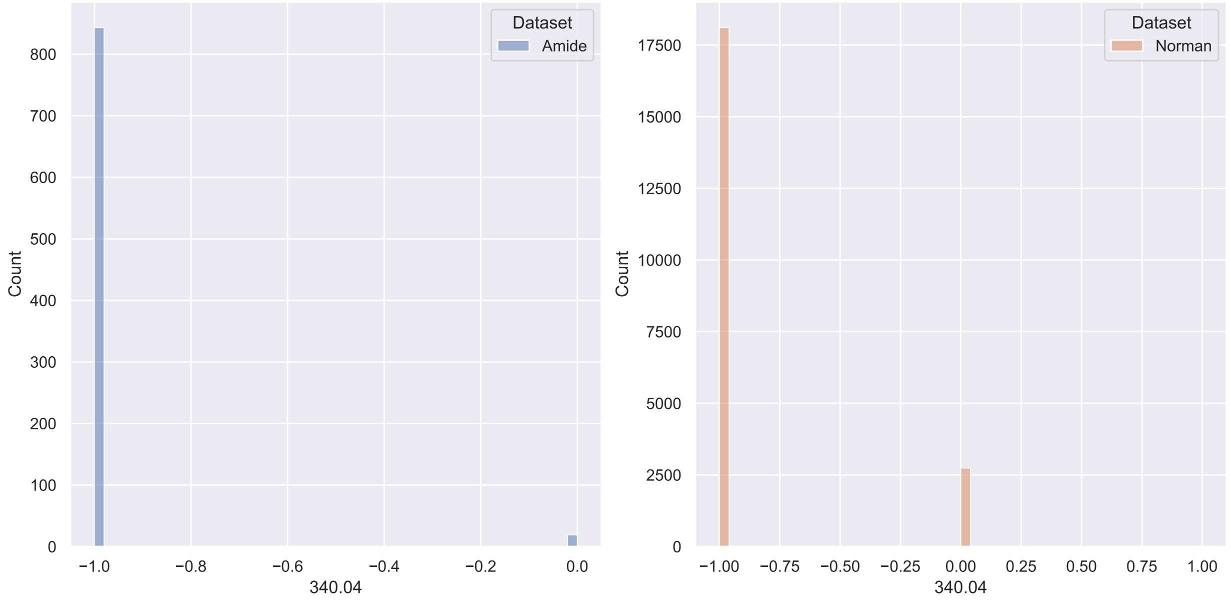

Figure 92: Distribution of neutral loss 340.04 for Amide dataset (left) and Norman dataset (right).

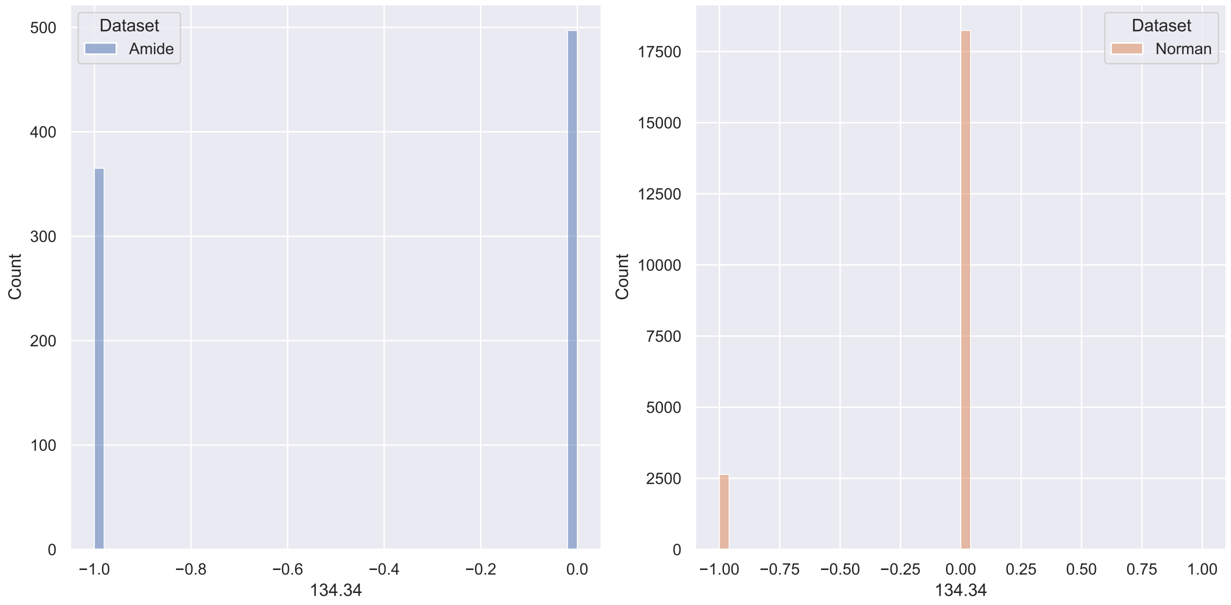

Figure 93: Distribution of neutral loss 134.34 for Amide dataset (left) and Norman dataset (right).

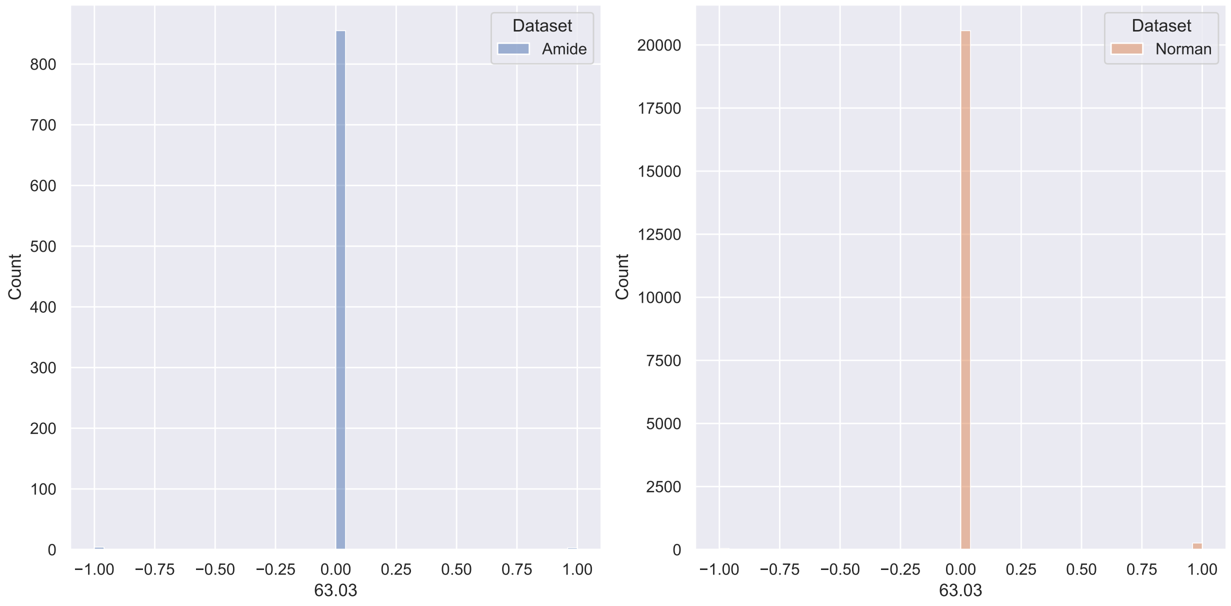

Figure 94: Distribution of neutral loss 63.03 for Amide dataset (left) and Norman dataset (right).

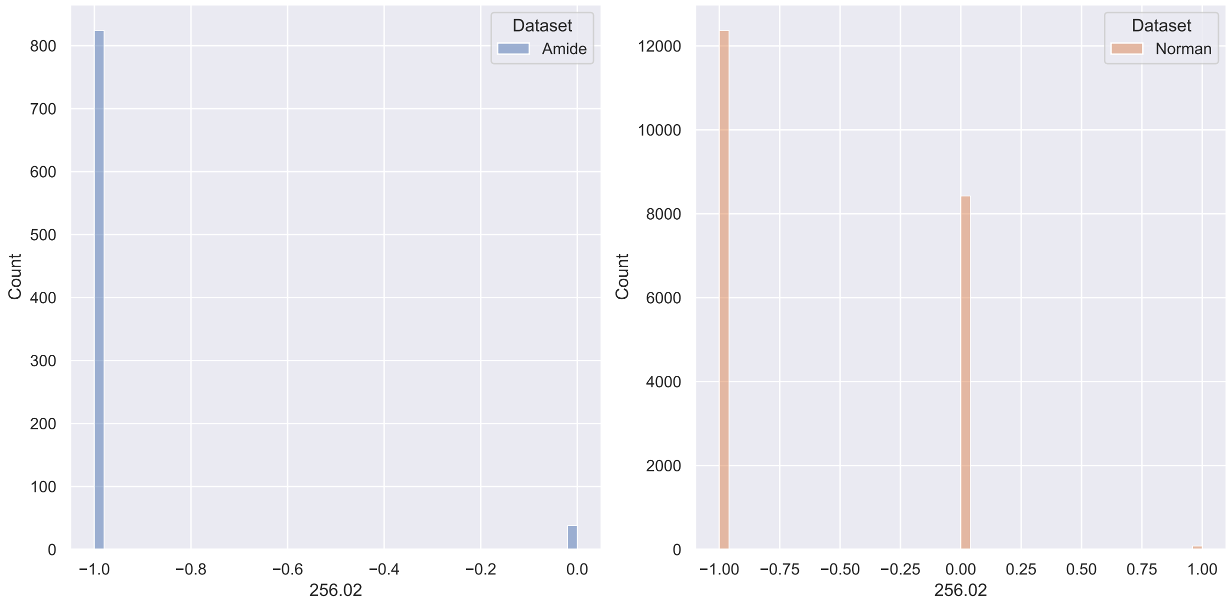

Figure 95: Distribution of neutral loss 256.02 for Amide dataset (left) and Norman dataset (right).

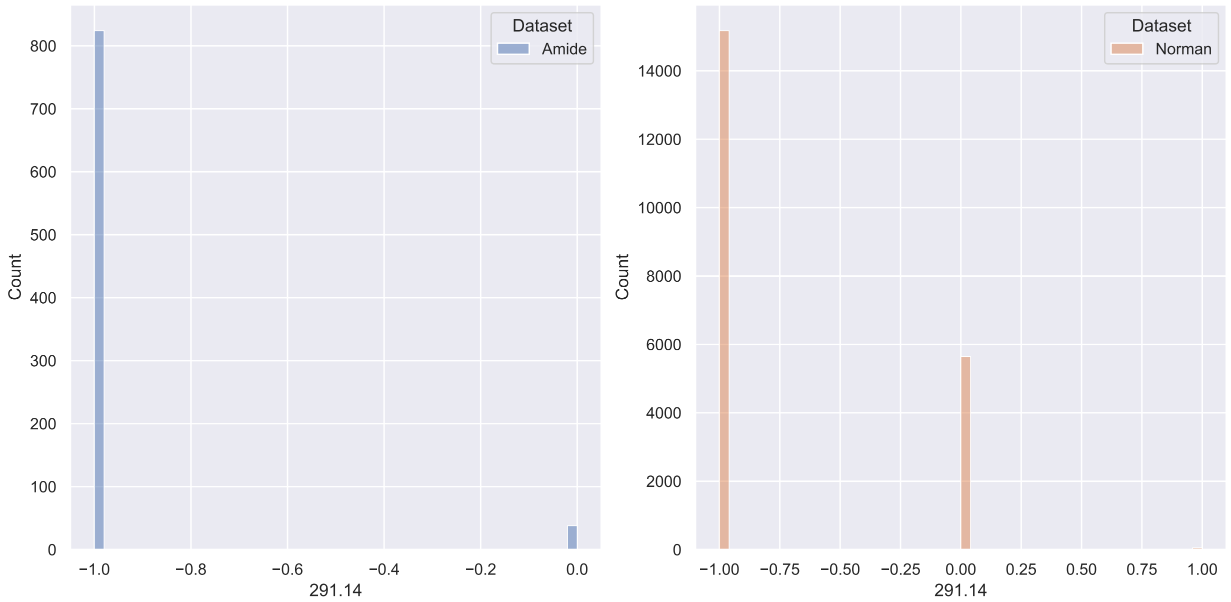

Figure 96: Distribution of neutral loss 291.14 for Amide dataset (left) and Norman dataset (right).

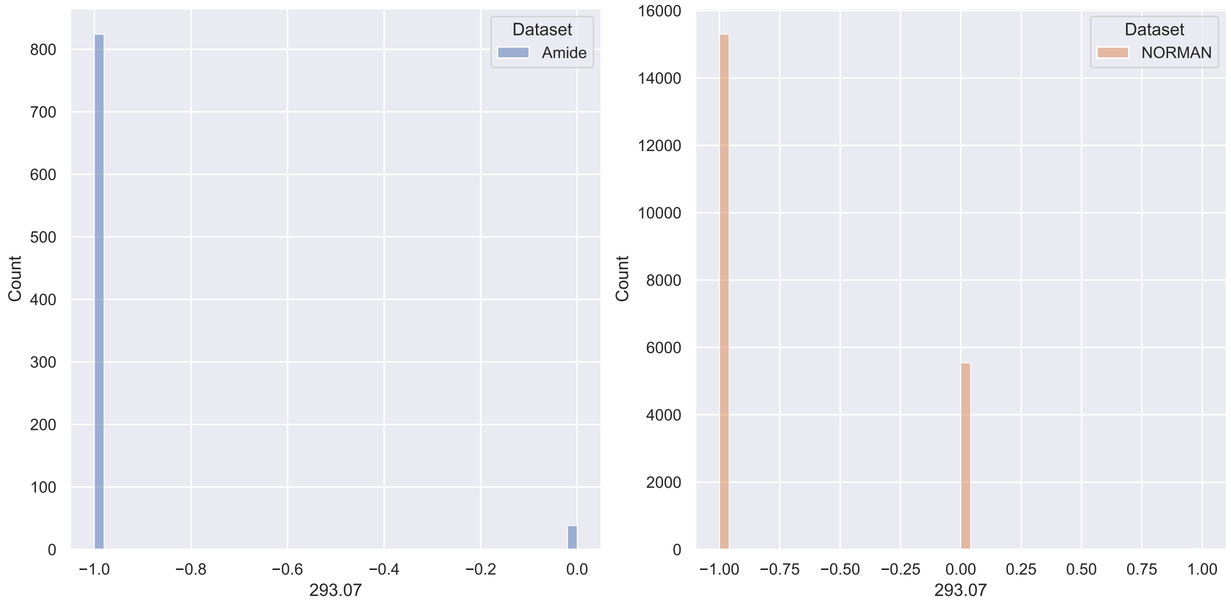

Figure 97: Distribution of neutral loss 293.07 for Amide dataset (left) and Norman dataset (right).

S5. Interpretation of Selected Descriptors for Descriptor Based model

This section shows the feature importances of the features used in the descriptor based CatBoost model. The features where computed using the PredictionValuesChange method in CatBoost [4]. In this method, for each feature, the average change in the prediction, if the feature value changes is computed. The bigger the value of the feature importance, the bigger on average its change is to the prediction value, if this feature is changed. Feature importances are normalized, so that the sum of feature importances adds up to 100.

[4] https://catboost.ai/en/docs/concepts/fstr#regular-feature-importance

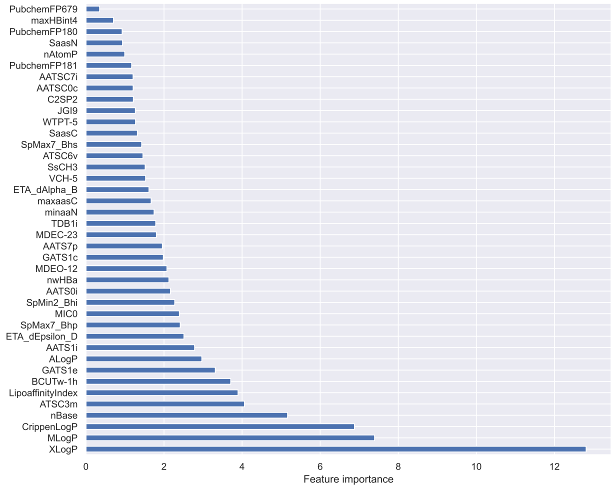

Figure 98: Feature importance plot of descriptor CatBoost model

S6. Interpretation of Selected CNLs for CNL Based model

This section shows the feature importances of the features used in the CNL based CatBoost model. The features where computed using the PredictionValuesChange method in CatBoost [5]. In this method, for each feature, the average change in the prediction, if the feature value changes is computed. The bigger the value of the feature importance, the bigger on average its change is to the prediction value, if this feature is changed. Feature importances are normalized, so that the sum of feature importances adds up to 100. Only the 50 most importance features are shown. We note that all feature importances are stored in the models (in the model object) that can be found on our GitHub page [6].

[5] https://catboost.ai/en/docs/concepts/fstr#regular-feature-importance
[6] https://github.com/Jimbo994/NL2RI

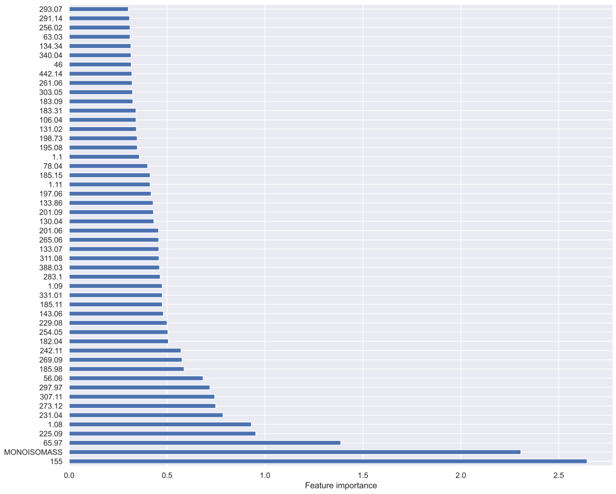

Figure 99: Feature importance plot of CNL CatBoost model
